# A single-cell level comparison of human inner ear organoids with the human cochlea and vestibular organs

**DOI:** 10.1016/j.celrep.2023.112623

**Published:** 2023-06-07

**Authors:** Wouter H. van der Valk, Edward S.A. van Beelen, Matthew R. Steinhart, Carl Nist-Lund, Daniel Osorio, John C.M.J de Groot, Liang Sun, Peter Paul G. van Benthem, Karl R. Koehler, Heiko Locher

**Affiliations:** 1 OtoBiology Leiden, Department of Otorhinolaryngology and Head & Neck Surgery, Leiden University Medical Center, 2333 ZA Leiden, the Netherlands; 2 The Novo Nordisk Foundation Center for Stem Cell Medicine (reNEW), Leiden University Medical Center, 2333 ZA Leiden, the Netherlands; 3 Department of Otolaryngology, Boston Children’s Hospital, Boston, MA 02115, USA; 4 F.M. Kirby Neurobiology Center, Boston Children’s Hospital, Boston, MA 02115, USA; 5 Department of Otolaryngology-Head and Neck Surgery, Harvard Medical School, Boston, MA 02115, USA; 6 Department of Otolaryngology-Head and Neck Surgery, Indiana University School of Medicine, Indianapolis, IN 46202, USA; 7 Medical Neuroscience Graduate Program, Indiana University School of Medicine, Indianapolis, IN 46202, USA; 8 Program in Neuroscience, Harvard Medical School, Boston, MA 02115, USA; 9 Research Computing, Department of Information Technology, Boston Children’s Hospital, Boston, MA 02115, USA; 10 Department of Plastic and Oral Surgery, Boston Children’s Hospital, Boston, MA 02115, USA; 11 Senior author; 12 Lead contact

## Abstract

Inner ear disorders are among the most common congenital abnormalities; however, current tissue culture models lack the cell type diversity to study these disorders and normal otic development. Here, we demonstrate the robustness of human pluripotent stem cell-derived inner ear organoids (IEOs) and evaluate cell type heterogeneity by single-cell transcriptomics. To validate our findings, we construct a single-cell atlas of human fetal and adult inner ear tissue. Our study identifies various cell types in the IEOs including periotic mesenchyme, type I and type II vestibular hair cells, and developing vestibular and cochlear epithelium. Many genes linked to congenital inner ear dysfunction are confirmed to be expressed in these cell types. Additional cell-cell communication analysis within IEOs and fetal tissue highlights the role of endothelial cells on the developing sensory epithelium. These findings provide insights into this organoid model and its potential applications in studying inner ear development and disorders.

## In brief

Van der Valk et al. highlight the potential of human stem cell-derived inner ear organoids for studying disorders. Single-cell transcriptomics identified various cell types linked to inner ear dysfunction, which are shared between organoids and the human inner ear. They offer an atlas for studying genetic inner ear disorders with organoids.

## Graphical Abstract

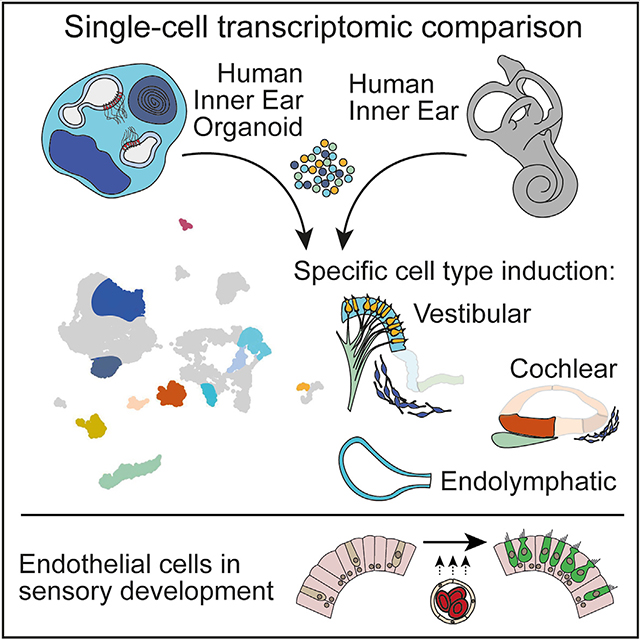

## INTRODUCTION

The inner ear comprises the cochlea and vestibular organs, which mediate sound perception and balance. Functional impairment of the inner ear is common, affecting more than 5% of the general population with hearing loss or balance disorders.^1,2^ The etiology of these inner ear disorders varies greatly depending on age, genetics, and environmental factors. Sensorineural hearing loss (SNHL) is one of the most common congenital disorders and affects more than 1 in 1,000 newborns.^3,4^ Hereditary SNHL can be attributed to either syndromic or nonsyndromic genetic causes.^5,6^ On the basis of the partial overlap of gene expression between the cochlea and the vestibular organs, it is not surprising that mutations in these deafness genes may also cause vestibular dysfunction, as seen in patients with DNFA9, DFNA11, DFNA15, and Usher syndrome.^7^

Despite research efforts to understand the mechanisms of genetic SNHL and to identify therapies, a significant challenge is the heterogeneity of SNHL.^8^ Current animal models are limited to modeling the effect of specific gene mutations. Culture models of differentiated human inner ear cells provide a valuable alternative and offer a way to assess the impact of SNHL genes on the development and function of the human inner ear, as discussed in recent reviews.^9–16^ Recent progress has been made on human inner ear organoids (IEOs), which can be derived from human pluripotent stem cells (hPSCs), either embryonic (human embryonic stem cells [hESCs]) or induced (human induced pluripotent stem cells [hiPSCs]), using differentiation strategies that result in cultures containing inner ear-like cells.^9^ In addition, several SNHL gene mutations have been modeled in mouse and hPSC-derived otic culture systems.^17^ However, most reports focus primarily on hair cells, and it remains unclear to what extend PSC-based systems can be used to model the full range of congenital SNHL disorders.^17^

A promising system lies in the hPSC-derived IEOs.^18^ Reports using this model are limited to the use of one hESC and one hiPSC line, and their findings are largely restricted to hair cells and neurons.^18–20^ However, it is possible that the cellular heterogeneity within these IEOs is more extensive, as recent and ongoing work on the temporal cellular diversity of early IEO aggregates reveals its multilineage character.^21^ Our goal in the current study was to validate the protocol across multiple hPSC lines and determine the cellular variety and maturity of late-stage hPSC-derived IEOs (differentiation day 75 [D75] and later), focusing on the inner ear cell types. To this end, we provide a tool to determine early differentiation efficiency. We demonstrate on-target induction of inner ear cell types but likewise identify off-target induction of cranial skeletal myocytes, ependymal cells, and vascular endothelial cells. For comparison, we generated an age-matched single-cell transcriptomic atlas of the fetal human inner ear that captures the diversity of the whole membranous labyrinth. In addition, we include single-cell data from adult human vestibular organs to show the extent to which IEOs contain mature cell types of the human inner ear. Our comparative analysis reveals that organoids contain specific sensory and nonsensory vestibular epithelial types, including type I and type II vestibular hair cells, and periotic mesenchyme (POM). Surprisingly, we could identify a subset of cells resembling cochlear nonsensory epithelial cells. By analyzing the cell-cell communication within the organoid model, we found evidence for signaling pathway interactions between hair cells and endothelial cells that could be validated in the human inner ear data. Furthermore, we assigned the expression of known SNHL and balance disorder-associated genes and proteins. These results offer significant understanding into the IEO model system and its possibility for exploring development and disorders of the inner ear.

## RESULTS

### BMP signaling underlies variability in IEO induction across hPSC lines

**Figure 1.**
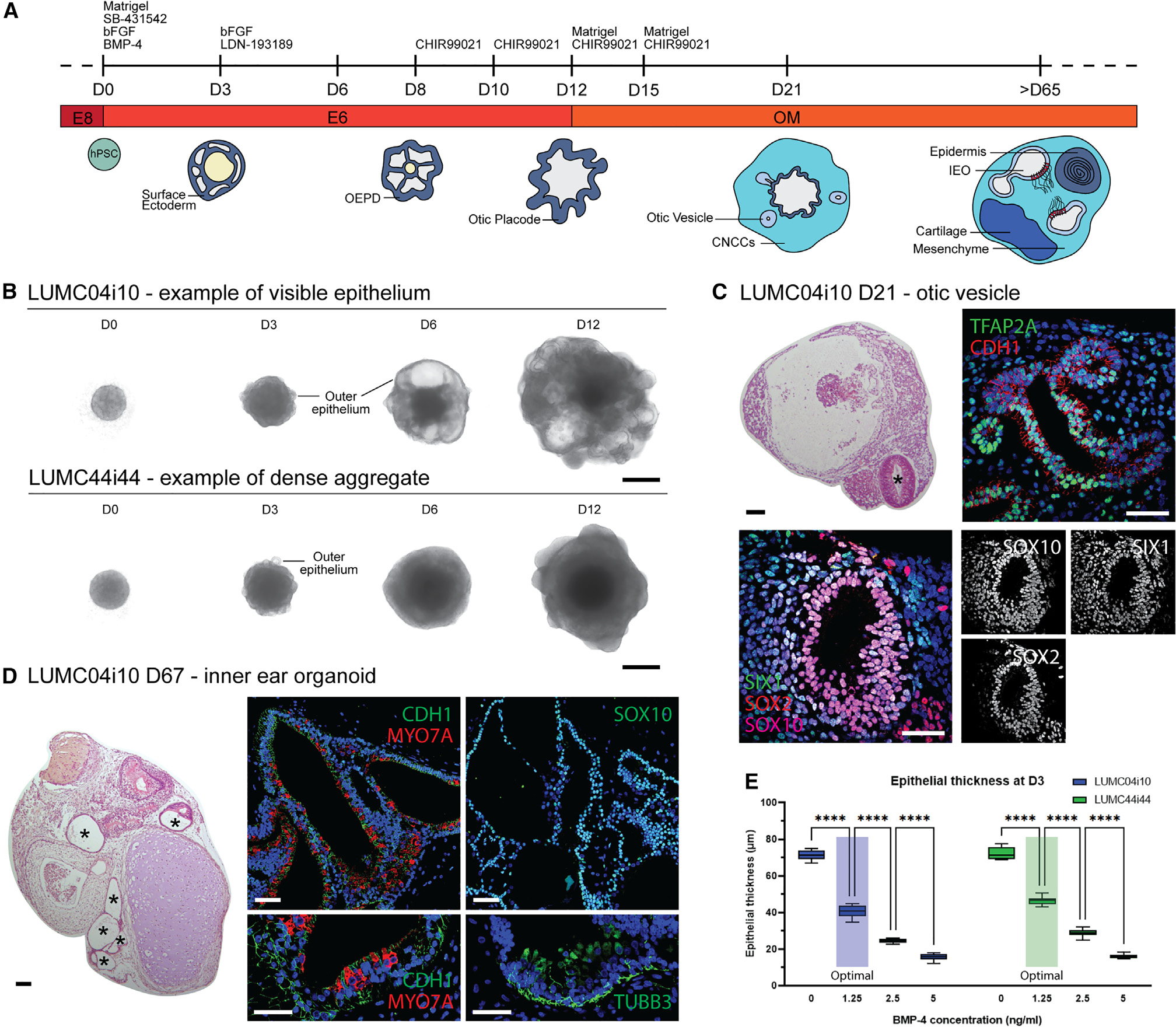
Otic induction efficiency depends on BMP-4 concentration (A) Overview of inner ear organoid differentiation. (B) Bright-field images of cell aggregates at different time points for two hiPSC lines showing morphological differences. Representative images from ≥8 aggregates (≥3 experiments). Scale bars, 500 μm. (C) Representative H&E and IHC images of D21 aggregates containing otic vesicles (asterisk) expressing TFAP2A, CDH1, SOX10, SIX1, and SOX2. Scale bars: H&E, 100 μm; IHC, 50 μm. (D) H&E and IHC images of D67 aggregates showing an IEO with epithelial vesicles (asterisks, CDH1^+^, SOX10^+^), containing hair cells (MYO7A^+^) and neurons (TUBB3^+^). Scale bars: H&E, 100 μm; IHC, 50 μm. (E) Epithelial thickness measurements of D3 aggregates treated with different BMP-4 concentrations at D0 for two cell lines. Efficient IEO induction achieved at 1.25 ng/mL BMP-4 (“Optimal”). Statistical significance analyzed using 2-way ANOVA with Šidák correction. Data considered significant if p < 0.05. n = 10 per datapoint shown in a min-max boxplot. Representative graph from two experiments. CNCCs, cranial neural crest cells; D, differentiation day; IEO, inner ear organoid; OEPD, otic-epibranchial placode domain. ****p ≤ 0.0001. H&E and IHC images (C and D) are representative of ≥6 aggregates (≥2 experiments). See also Figure S1.

We aimed to evaluate the robustness of differentiating inner ear epithelium from multiple hPSCs using a streamlined method for IEO induction (Figure 1A).^18,21^ Eight human healthy-control hPSC lines were used: one hESC line (WA01) and seven hiPSC lines (WTC-SOX2, WTC-GCaMP, LUMC04i10, LUMC44i44, GON0515–03, GON0926–02, and SAH0047–02). In adapting the protocol to a broader cohort of cell lines, we noted that the BMP-4 treatment at D0 was a critical determinant of successful IEO production. In our experience, BMP-4 activity differs between vendors and lots, hampering consistent differentiation efficiency. We sought to determine which morphological features during early differentiation could be used to evaluate IEO induction efficiency objectively. To this end, we treated the cell lines with BMP-4 concentrations ranging from 0 to 20 ng/mL, and we followed morphologic features up to D12 (Figure 1B). The most efficient BMP-4 concentration was determined by the presence of otic vesicles at D21 or the presence of more mature IEOs at D65 and later, using H&E staining or immunohistochemistry (IHC) (Figures 1C and 1D). The otic identity of inner ear vesicles at D21 could be confirmed by the expression of CDH1, TFAP2A, and SOX10,^22^ with additional SOX2 and SIX1 expression (Figure 1C). The IEOs of D65 and later were CDH1^+^ SOX10^+^, and contained MYO7A^+^ hair cells with TUBB3^+^ neurons that protrude toward hair cells (Figure 1D). The highest differentiation efficiency was reached at a BMP-4 concentration of 1.25 ng/mL for this set of experiments using BMP-4 from the same lot, with either otic vesicles or epithelial IEOs forming in all eight hPSC lines. We limited the measurement of morphological features following BMP-4 treatment to D3, as during the first 12 days, we noticed a variation in the morphology between cell lines (Figure 1B). LUMC04i10, WTC-SOX2, WTC-GCaMP, and SAH0047–02 aggregates contained an outer epithelial layer, the developing ectoderm,^18^ which could be clearly monitored over time until D12 (Figure 1B). LUMC44i44, WA01, GON0515–03, and GON0926–02 aggregates, however, became denser after D3, and the outer epithelial layer could not be followed over time (Figure 1B). We measured epithelial thickness, circularity, and area of the D3 aggregates *in vitro* (Figure S1A). From these morphological features, only epithelial thickness significantly differed between BMP-4 concentrations over all hPSC lines (Figures 1E and S1B–S1C). Thickness decreased with increasing BMP-4 concentrations, with efficient IEO differentiation being achieved with an outer epithelium layer that was on average 35.9 μm thick (SD = 6.2 μm, n = 10 aggregates per cell line, 8 cell lines). Size and circularity of the aggregate were also influenced by the BMP-4 concentration, however less consistent over all cell lines (Figure S1C). Depending on the BMP-4 lot or vendor, however, concentrations up to 20 ng/mL had to be used to reach the same epithelial thickness and subsequent efficient induction of otic vesicles and IEOs (data not shown). In conclusion, optimal BMP-4 concentration at D0 can be inferred from morphological changes as early as D3 and not necessarily by the absolute concentration of BMP-4. A range of 1.25–20 mg/mL is acceptable depending on the vendor, handling, and storage (see STAR Methods). Using aggregates with the optimal outer epithelium thickness, we efficiently induced otic vesicles in all hPSC lines tested.

### Single-cell and single-nucleus RNA sequencing unravel the cellular diversity of IEOs

To comprehensively characterize the cell types that arise in the efficiently differentiated aggregates, we performed single-cell RNA sequencing (scRNA-seq) as well as single-nucleus RNA sequencing (snRNA-seq) on D75–D110 aggregates derived from four hPSC lines (WA01, WTC-SOX2, WTC-GCaMP, and LUMC04i10). The WTC-SOX2 and WTC-GCaMP lines are commonly used for our experiments and therefore included, although their reporter functions are beyond the scope of this paper. We decided to include snRNA-seq in addition to scRNA-seq as certain cell types, such as epithelial cells, might be better presented by the single nucleus approach.^23,24^ More important, both methods allow similar discrimination of cell types when intronic sequences are included in snRNA-seq analyses.^25,26^ In addition to dissociation of whole aggregates into single cells, we used another approach by which aggregates were first cut in slices using a vibratome. This approach allowed us to select sections that contain IEO-like vesicles by bright-field evaluation before dissociation into single cells.

Using differentially expressed genes and cell type-specific marker genes for annotation, we captured a similar cell type heterogeneity in the scRNA-seq (Figure S2) and snRNA-seq data (Figure S3). The vibratome approach, however, did not result in a relatively larger number of otic cell types (Figure S2B). We identified some variation between the scRNA-seq and snRNA-seq approach: using scRNA-seq of the WTC-SOX2 and WTC-GCaMP hiPSC lines, clusters of cycling cells and cells with a high ribosomal content were identified that could not be identified using the snRNA-seq approach in the WA01 and LUMC04i10 hiPSC lines. Similarly, adipocytes could not be identified using scRNA-seq of the WTC-SOX2 and WTC-GCaMP hiPSC lines but were seen in the snRNA-seq approach.

**Figure 2.**
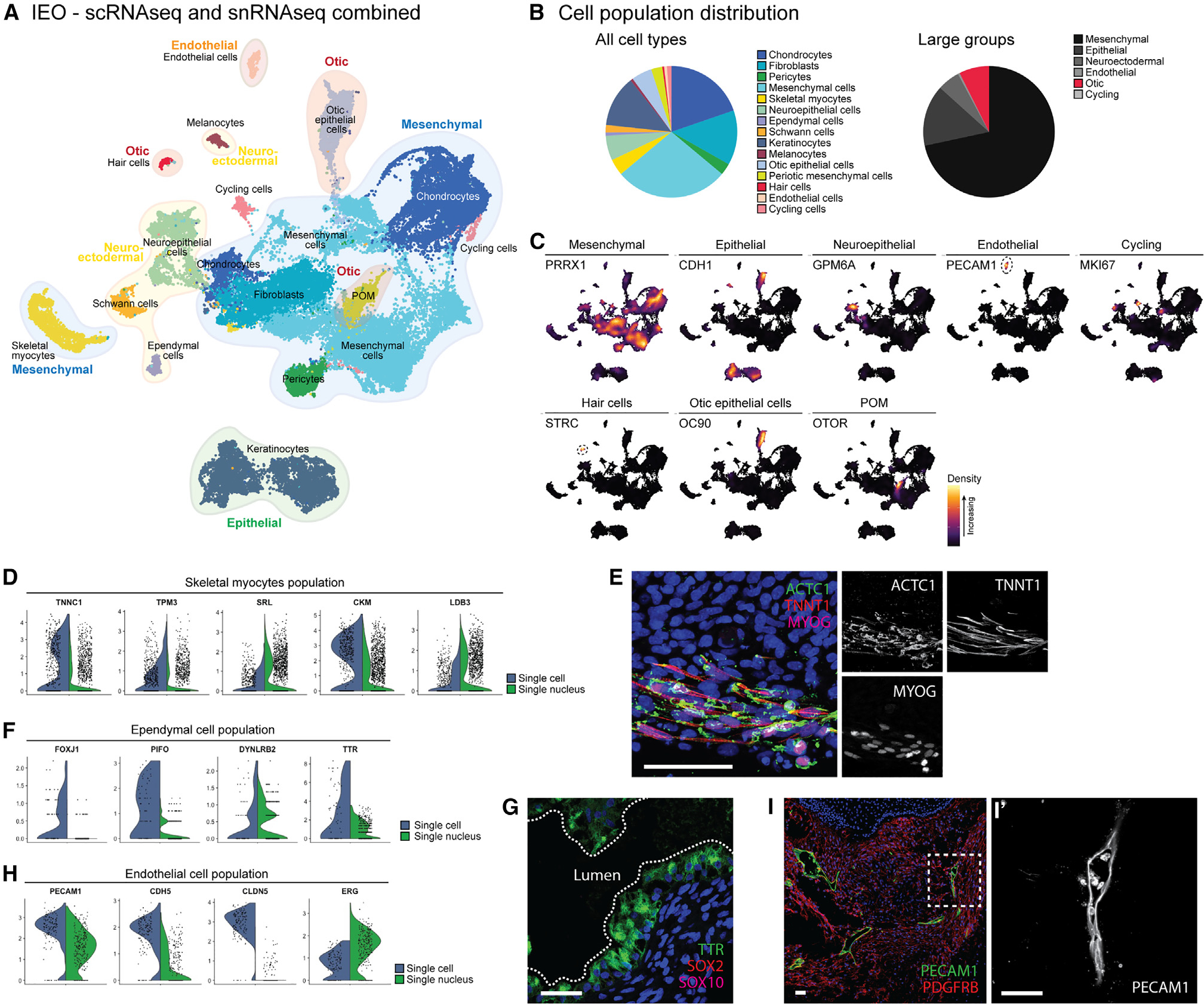
Single-cell transcriptomic analysis reveals IEO cellular diversity (A) Uniform manifold approximation and projection (UMAP) plot showing scRNA-seq and snRNA-seq data of D75-D110 IEOs with cell type annotations. (B) Relative contribution of cell types and large groups. (C) Marker genes for cell type annotation. (D) Skeletal myocyte cluster highlighted by skeletal myocyte marker genes. (E) ACTC1^+^ TNNT1^+^ MYOG^+^ skeletal myocytes in a D75 aggregate. Scale bar, 50 μm. (F) Ependymal cell cluster with marker gene expression. (G) TTR^+^ SOX2^−^ SOX10^−^ ependymal cells in a D75 aggregate. Scale bar, 50 μm. (H) Endothelial cluster with marker genes. (I) PECAM1^+^ endothelial cells forming luminal-like structures in a D75 aggregate (I′). Scale bars, 50 μm. POM, periotic mesenchyme. IHC images (E, G, and I) are representative of n ≥ 6 aggregates (≥2 experiments). See also Figures S2–S5 and S8.

As we were able to identify similar cell types with both approaches, we integrated the data to a combined dataset of sequenced cells and nuclei for further analyses (Figures 2A–2C and S4). Of the cell types, 8.3% (3.7%–11.1% between cell lines) assigned to an otic identity that includes otic epithelium, hair cells, or POM (Figure 2C). The unique transcriptomic signature of hair cells in the otic epithelium is demonstrated by their distinct clustering from the epithelial cells, similar to single-cell studies of the mouse and human inner ear.^27,28^ We wondered if the other populations contained cells reminiscent of the inner ear spatial environment, thus we set out to analyze these populations. Most of these cell types were of mesenchymal origin (68.0%; Figure 2B). This group contained a cluster of myocytes (*TNNC1*, *TPM3*, *SRL*, *CKM*, and *LDB3*)^29^ (Figure 2D). The presence of these myocytes corresponds with our observations of irregular contractions in later stage aggregates *in vitro* (data not shown). This mesenchymal population expressed skeletal myocyte-specific genes (*TNNT1*, *MYBPC1*, *MYOT*, *RYR1*, and *JPH1*) (Figure S5A),^29^ with a marked lack of expression of marker genes for either smooth muscle cells (*ACTA2*, *MYOCD*, and *MYH11*)^30^ or cardiomyocytes (*MYH6*, *MYL7*, *TNNI3*, *MYBPC3*, and *CACNA1C*).^29^ In line with the fetal skeletal myocyte gene expression of *MYL4*, *ACTC1*, and *TNNT2* (Figure S5A),^31–33^ we could identify different transcriptional stages of skeletal myogenic differentiation within this population (Figure S5B): myocyte precursors (*PAX7*), myoblasts/myocytes (*MYOD1*, *MYOG*), and myotubes (*MYL2*, *MYH2*, *MYH7*, *ITBG1*).^34^ Interestingly, when identifying upstream genetic regulators that control cranial myogenesis, we observed *TBX1* and *PITX2* gene expression, with a relative absence of *PAX3* (Figure S5C), limiting the possible identity to that of cranial skeletal myocytes, as reviewed by A. Grimaldi and colleague.^35^ Moreover, moderate expression of *NKX2–5* could be seen in this cluster (data not shown). Although *NKX2–5* expression is well established in cardiomyocyte development, expression on the protein and mRNA level has been described in developing cranial skeletal myocytes.^36^ Using IHC, we confirmed the presence of ACTC1^+^ TNNT1^+^ MYOG^+^ skeletal myocytes in our aggregates (Figure 2E). The mesenchymal population consists also of a chondrocyte population (*MATN4, COL9A1, SOX6, SOX9, COL2A1*) which contains an early mature signature (*CNMD, EPYC, FRZB*) (Figure S5D) with more mature genes such as *ISG15*, *IFI6*, and *MX1* expressed only in a fraction of the cells^37^ (Figure S5E). Additionally, we identified fibroblasts (*COL1A1, PDGFRA, TWIST2, APCDD1, DPT*) and pericytes (*CSPG4, PDGFRB, RGS5, KCNJ8, ABCC9*) forming distinct clusters within the mesenchymal cell types (Figures S5F and S5G).

The second largest group within the aggregate are the epithelial cells consisting of keratinocytes and otic epithelial cells (12.6%). The keratinocytes within the aggregate showed a similar cell type-diversity to the skin organoids.^38^ Within this population expressing *CDH1, KRT5*, and *KRT19*, we could identify basal (*CXCL14*), intermediate (*KRT1*), and peridermal (*KRT4*) keratinocytes (Figure S5H).

Neuroectodermal cell types are involved in certain types of SNHL, such as Waardenburg syndrome affecting the melanocyte lineage, but also in neurofibromatosis type II, which is characterized by neoplastic Schwann cells.^39^ To get a better understanding of these populations, we set out to determine how mature these cell types are in our IEOs. The neuroectodermal group (9.4%) consisted of neuroepithelial cells, ependymal cells as well as Schwann cells and melanocytes. The neuroepithelial population could be divided into a group of neuroepithelial progenitor cells (*SOX2* and *SNHG11*) and more mature central (*GPM6A, MATK*, and *RASFRF1*) and peripheral (*PRPH* and *PIRT*) neurons (Figure S5I).^40^ These latter expressed genes for ion channels known to be enriched in the peripheral nervous system, specifically in sensory neurons (*P2RX3, SCN7A*, and *SCN10A*).^41^ When analyzing semi-thin sections, we identified occasional groups of neurons in the stroma surrounding the IEOs. They contain a distinct cell body with a large, round nucleus and a single dendritic process, suggestive for neurons (Figure S5J). The dendritic processes were surrounded by cells with elongated, flat nuclei with morphologies similar to Schwann cells. Using transmission electron microscopy (TEM), we observed evidence of Schwann cells associated with these neurons near the otic vesicles (Figure S5K). The presence of these Schwann cells was confirmed by the transcriptomics data, with a Schwann cell population expressing marker genes (*SOX10, ERBB3, S100B*, and *PLP1*), as well as genes for immature (*NGFR, NCAM1, L1CAM*, and *CDH2*) and (pro)-myelinating Schwann cells (*POU3F1, CDKN1C, MPZ, PTN*, and *CRYAB*) (Figure S5L).^42^ We also discovered a population of ependymal cells that expressed *FOXJ1, PIFO, DYNLRB2*, and *TTR* (Figure 2F).^43^ We confirmed the presence of TTR^+^ SOX2^−^ SOX10^−^ ependymal cells forming vesicle-like structures (Figure 2G), in contrast to the TTR^−^ SOX2^+^ SOX10^+^ otic vesicles. The last neuroectodermal population was composed of melanocytes, which expressed genes of various stages of melanocyte differentiation, including marker genes for melanoblasts (*SOX10, EDNRB, DCT*, and *MITF*), melanocytes (*KIT, PAX3*, and *EDNRB*) and that of differentiated fully pigmented melanocytes (*TYRP1, TYR*, and *GPR143*) (Figure S5M).^44,45^ Although melanocytes are generally known for their role in skin pigmentation,^38^ a subset of melanocytes incorporates into the ionic regulatory epithelium in the inner ear^46,47^; hence, we sought to locate this population in IEOs. Interestingly, melanocytes (MLANA^+^) were found within or closely related to the SOX10^+^ IEOs (Figure S5N), similar to the developing human fetal vestibular organs (around fetal week 10) and cochlea (around fetal week 11).^46,47^

We discovered a population of endothelial cells in the IEOs using the transcriptomic data that could be reminiscent of the blood-labyrinth barrier of the inner ear. This population (0.7%) expressed known endothelial markers *PECAM1*, *CDH5*, *CLDN5*, and *ERG* (Figure 2H),^48^ with concomitant expression of vascular marker genes (*FLT1, VWF, NOTCH4,* and *NRP1*)^48,49^ and relative absence of lymphatic marker genes (*PROX1* and *PDPN*) (Figure S5O).^50^ *LYVE1* and *FLT4*, both expressed in this population, are known lymphatic-specific genes, although these genes have been observed during fetal vascular endothelial development.^51,52^ We could confirm the presence of PECAM1^+^ endothelial cells within the aggregate forming lumen-containing structures throughout the aggregate (Figure 2I).

In summary, using different hPSC lines, we could robustly induce multilineage cell types within the IEOs. The cell types we identified are known to be expressed in the environment of the inner ear, although we could not grant all of them with an “inner ear identity.” In addition to the putative cranial identity of the skeletal myocytes, we identified gene expression profiles that match embryonic or fetal development, for instance for the skeletal myocytes, chondrocytes, and vascular endothelial cells. WTC-GCaMP and WTC-SOX2 aggregates (which share a parental genome) showed a similar cell type distribution based on scRNA-seq data (Figure S4B). However, this distribution differed from the LUMC04i10 and WA01 cell line that are used for snRNA-seq. This could be due to variability in dissociation method (either single cell or single nucleus), or cell line variation. The latter is supported by the difference we observe between the WA01 and LUMC04i10 cell line, notably the large neuroepithelial cell type cluster found in the WA01 cell line and a relatively smaller mesenchymal cluster. In this study, we did not perform thorough batch-to-batch variation tests to rule out the role of cell lines or the transcriptomics method used.

### Generation of a single-cell atlas of fetal and adult human inner ear tissues

**Figure 3.**
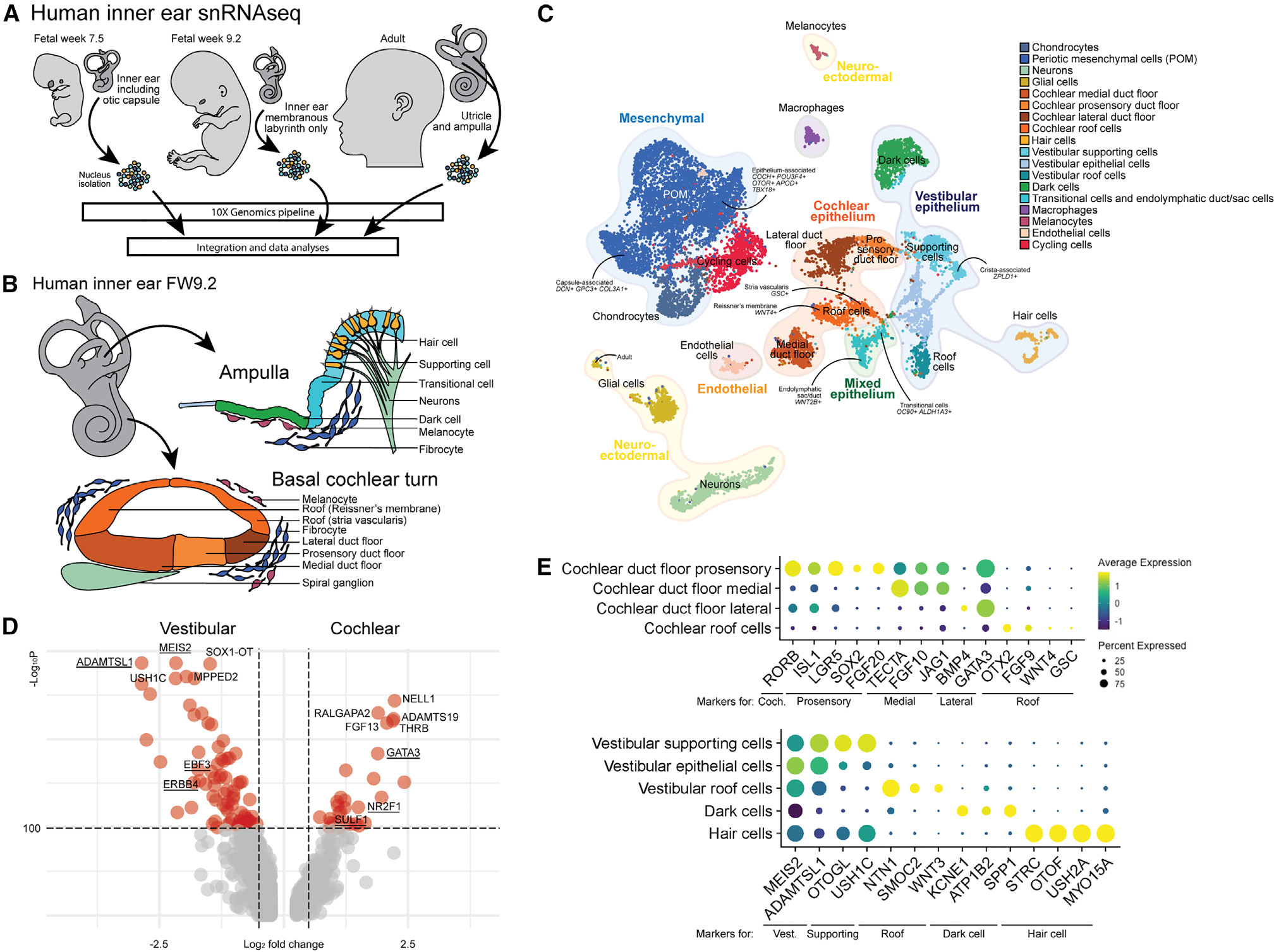
Generation of the human fetal and adult inner ear single-cell atlas (A) Experimental overview of fresh human inner ear tissue collection from FW7.5 (n = 1), FW9.2 (n = 1), and adult donor (n = 1). (B) The human inner ear at FW9.2, illustrating cellular diversity in the vestibular ampulla and basal turn of the cochlea. (C) UMAP plot of combined human inner ear datasets with cell type annotations. (D) Volcano plot showing differentially expressed genes between vestibular supporting cells and cochlear duct floor. Underlined genes are described to be differentially expressed between vestibular and cochlear cell types. (E) Dot plot displaying standardized expression of known marker genes in cochlear and vestibular cell types. Coch., cochlear; Vest., vestibular. See also Figure S6.

To compare cell type diversity within IEOs to those found in the native human inner ear, we carried out snRNA-seq on fresh inner ear tissue collected at different stages of human inner ear development. We collected one fetal inner ear of fetal age week 7.5 (FW7.5), one fetal inner ear of FW9.2, and an ampulla and utricle from a 47-year-old donor (Figure 3A). At FW9.2, we would expect that the mature cochlea is still in development and the epithelial populations could be separated out in different domains (Figure 3B). The cochlear duct floor at this stage divides in a medial, prosensory, and lateral domain, giving rise to the future Kölliker’s organ, organ of Corti, and outer sulcus, respectively. In addition, the roof of the cochlear duct will give rise to Reissner’s membrane and the stria vascularis, as reviewed by E.C. Driver and colleague.^53^ Vestibular development, which precedes that of the cochlea, has a more mature phenotype with distinct epithelial cell types at FW9.2.^46,54^

We used differentially expressed genes and marker genes (Figures S6A–S6D) as well as recently published single-cell datasets of the mouse inner ear^24,27,28,55,56^ to annotate the populations of the integrated inner ear dataset (Figure 3C). We identified a mesenchymal population, various epithelial populations of both cochlear and vestibular identity, hair cells, neurons, glial cells, cycling cells, endothelial cells, macrophages, and melanocytes (Figures 3C and S6). To confirm the vestibular versus the cochlear identity of the epithelial cell population, we analyzed the differential gene expression between the putative cochlear duct floor and vestibular supporting cells (Figure 3D). *ADAMTSL1, MEIS2, EBF3,* and *ERBB4* were genes highly expressed within vestibular populations, in contrast to *GATA3, NR2F1*, and *SULF1* which are present in cochlear populations, as has been shown on a single-cell level for the developing mouse inner ear.^27,56^ In addition, the cochlear-specific RORB is expressed in the cochlear duct floor (Figure 3E).^56,57^ The cochlear cell types could be separated (Figure 3E) into a medial (*TECTA, FGF10*, and *JAG1*), prosensory (*ISL1, LGR5, SOX2*, and *FGF20*) and lateral domain of the duct floor (BMP4 and GATA3) as well as the cochlear roof (*OTX2*, *FGF9*, *WNT4*, and *GSC*), based on data from the developing mouse cochlea.^56,58–63^ The vestibular population is composed of putative supporting cells (*OTOGL* and *USH1C*) with a crista-associated *ZPLD1*^+^ supporting cell population, roof cells (*NTN1*, *SMOC2*, and *WNT3*) and dark cells (*KCNE1, ATP1B2*, and *SPP1*) expressing marker genes that have been described for the developing mouse vestibular organs.^27,55^ Moreover, our data unveiled the presence of a distinct cluster of epithelial cells exhibiting a vestibular signature. Despite analyses, we were unable to ascertain their precise identity and thus designated them as “epithelial cells.” It is plausible that this population could comprise canal epithelium, which lacks a well-defined signature in existing literature. The remaining epithelial cluster was composed of a mixed population of endolymphatic duct/sac cells (*WNT2B*) and vestibular transitional cells (*OC90* and *ALDH1A3*).^27,64^ We validated a subset of differentially expressed genes and marker genes in an FW9 human fetal inner ear (Figure S6E). In the utricle, we confirmed spotty expression of LGR6 specifically in the vestibular sensory domain, which also contains MYO7A^+^ hair cells. Additionally, we observed OC90^+^ DACH1^−^ transitional cells and OC90^+^ DACH1^+^ dark cell epithelium. In the cochlea, we confirmed the presence of the GATA3^+^ SOX2^+^ prosensory domain, as well as TECTA^+^ expression in the tectorial membrane and apical expression in the medial domain of the duct floor. We also identified GATA3^+^ SOX2^−^ expression in the lateral duct floor, and OC90^+^ OTX2^+^ expression in the roof. Taken together, our IHC findings validate the expression of differentially expressed genes and marker genes in the human fetal inner ear, further supporting the accuracy of our annotations.

Within the mesenchymal population, chondrocytes and a large cluster of POM cells were identified. The chondrocytes were mostly identified in the FW7.5 inner ear, as during preparation of this sample the otic capsule was not removed. Similar to the developing mouse vestibular organs, different cell populations could be identified in the remaining mesenchymal population, with a transcriptomic difference between epithelium-associated mesenchyme and capsule-associated mesenchyme.^27^ We observed capsule-associated POM (*DCN, GPC3*, and *COL3A1*) clustering separate from the epithelium-associated POM (*COCH, POU3F4, OTOR, APOD*, and *TBX18*). We were not able to distinguish between the development of vestibular and cochlear mesenchyme, because of the unavailability of reference data. Nonetheless, our dataset represents a single-cell level analysis of early-stage inner ear development in combination with human adult vestibular organs. As such, it is a valuable resource not only for organoid comparative analysis, but also for the broader scientific community seeking to understand the complex mechanisms underlying inner ear development.

### POM within the IEOs is associated with the sensory epithelium

To assess how IEOs resemble the human inner ear, we used Symphony, a tool for single-cell reference mapping.^65^ Using this R package, IEO-derived cell types were mapped as a query to the reference atlas of the human inner ear. We performed these analyses separately for the otic mesenchymal, epithelial, and hair cell populations.

**Figure 4.**
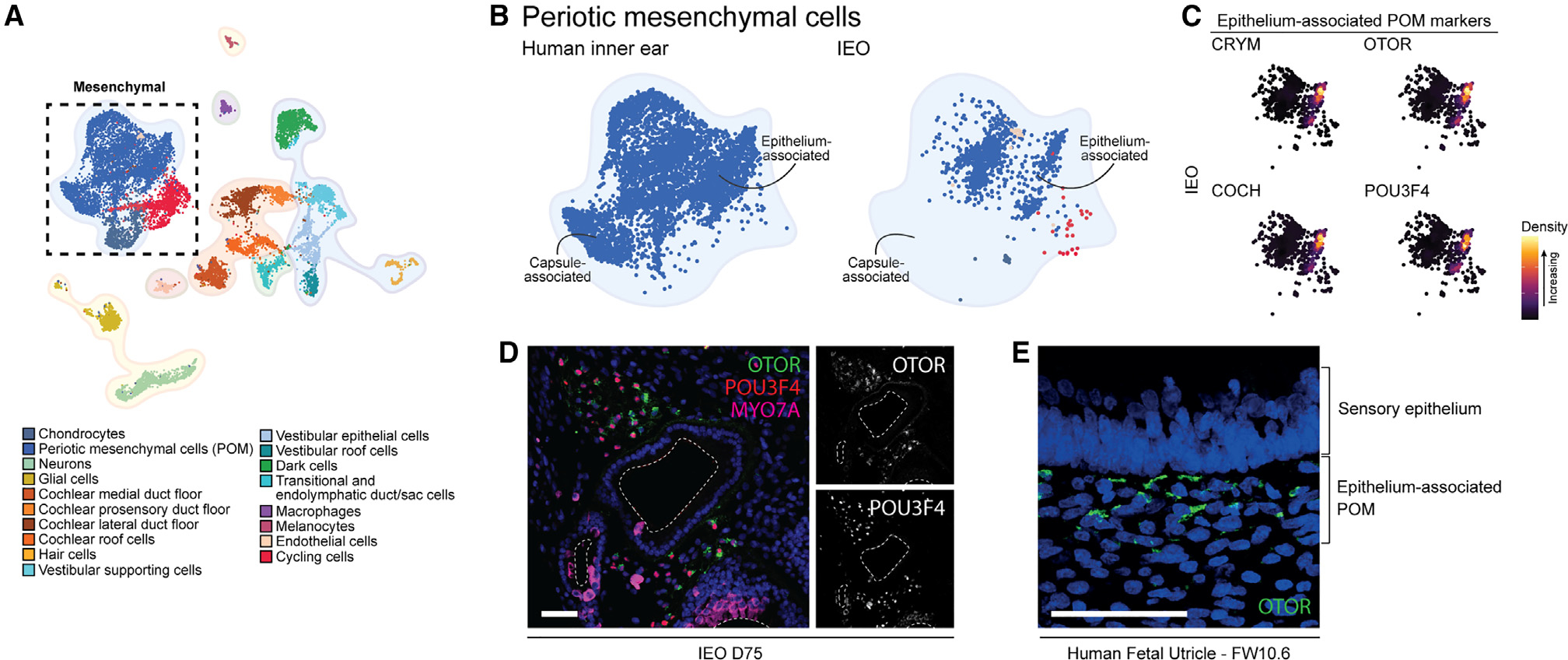
IEO-derived POM resembles human fetal epithelium-associated POM (A) UMAP plot of annotated human inner ear atlas highlighting mesenchymal cell types. (B) UMAP plot comparing reference POM (left) and mapped IEO-derived POM population (right) using Symphony. (C) Density plots showing expression of epithelium-associated markers in the IEO-derived POM population. (D) OTOR^+^ POU3F4^+^ mesenchymal cells surrounding IEO-vesicles containing MYO7A^+^ hair cells at D75. Scale bar, 50 μm. (E) OTOR^+^ mesenchymal cells beneath fetal utricle sensory epithelium (FW10.6). Scale bar, 20 μm. POM, periotic mesenchyme. IHC images are representative of n ≥ 6 aggregates (≥2 experiments) (D) or n ≥ 2 human inner ear tissues of matching fetal age (E).

We mapped the putative POM cluster present in the IEO dataset to the human inner ear atlas reference (Figures 4A and 4B). A large overlap was present with the reference POM cluster, more specifically with the epithelium-associated POM. Expression of *CRYM, OTOR, COCH*, and *POU3F4* within the IEO-derived POM confirmed the epithelium-associated identity (Figure 4C). The other region overlapping was composed of POM that was found in the human fetal inner ears (Figure S6B). We did not observe any overlap with the capsule-associated POM from the reference human inner ear atlas. To confirm the presence of the epithelium-associated POM, we performed additional IHC analyses of D75 IEOs. Surrounding inner ear vesicles containing MYO7A^+^ hair cells, we noticed the presence of OTOR^+^ POU3F4^+^ POM (Figure 4D). These OTOR-expressing cells resembled the OTOR^+^ mesenchymal cells *in vivo* that directly underlie the fetal vestibular sensory epithelium (Figure 4E).

### IEOs contain immature type I and type II vestibular hair cells

**Figure 5.**
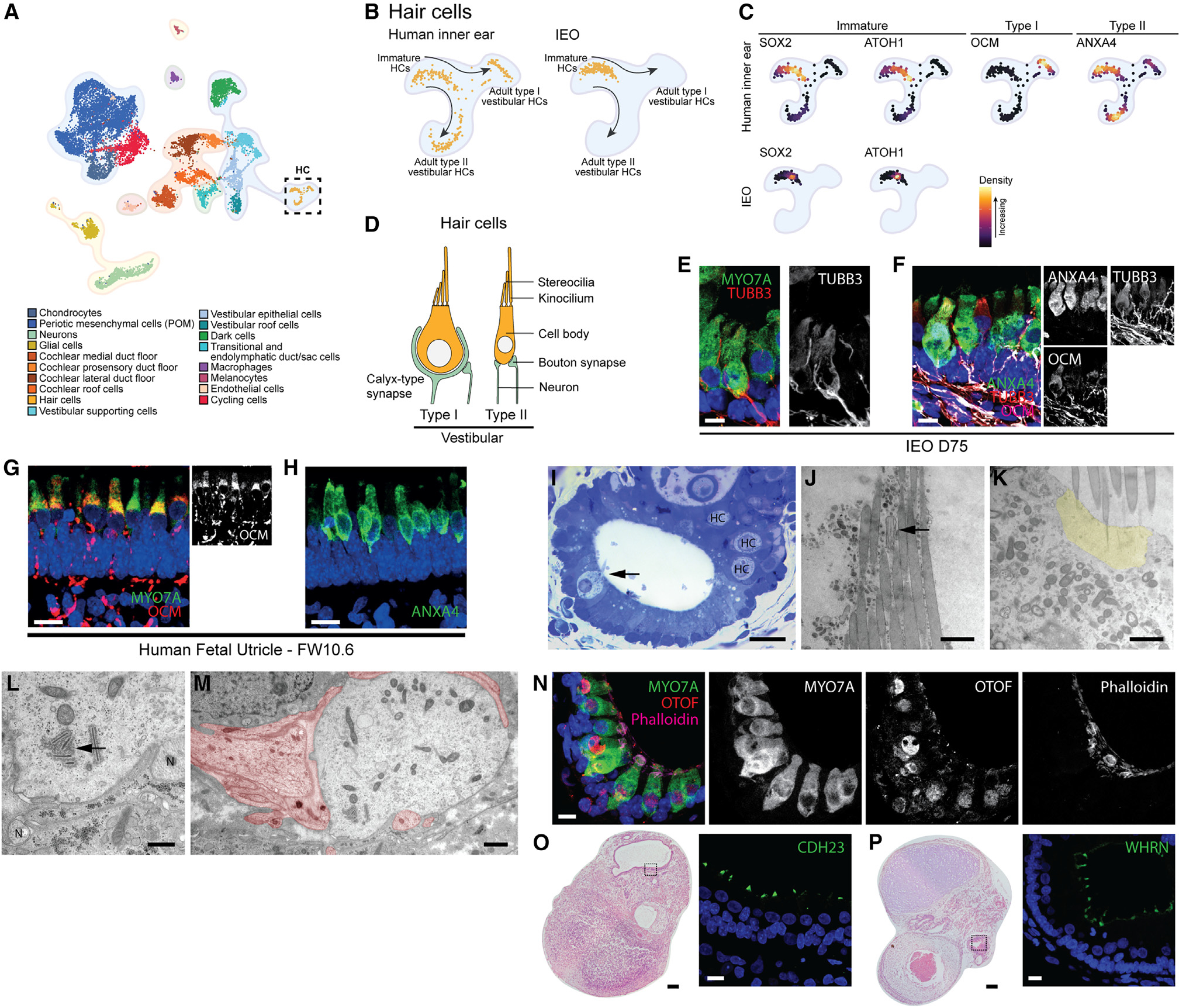
Immature type I and type II vestibular hair cells in IEOs (A) UMAP plot of annotated human inner ear atlas highlighting hair cells (HCs). (B) UMAP plot comparing reference hair cell population (left) and mapped IEO-derived hair cell population (right) using Symphony. (C) Density plots of reference hair cell population (left) for immature, type I, and type II vestibular markers, and density plots of mapped IEO-derived hair cells showing expression of immature markers. (D) Schematic depicting morphological differences between type I and type II vestibular hair cells. (E) Calyx-type synapse (TUBB3^+^) surrounding a hair cell (MYO7A^+^). Scale bar, 10 μm. (F) Expression of ANXA4 and OCM in hair cells of a D75 IEO, showing subsets with none, either, or both. Scale bar, 10 μm. (G) OCM expression in hair cells of human fetal utricle (FW10.6). Scale bar, 10 μm. (H) ANXA4 expression in human fetal utricle (FW10.6). Scale bar, 10 μm. (I) Methylene blue-azure II staining of a D74 IEO-vesicle containing hair cells (arrow and “HC”). Scale bar, 20 μm. (J) Hair bundle with stereocilia and kinocilium cross-section (arrow). Scale bar, 1 μm. (K) Apical region of a hair cell with cuticular plate (yellow) and stereociliary rootlets. Scale bar, 1 μm. (L) Basal region of a hair cell showing synaptic ribbons (arrow) and bouton-like synapses (“N”). Scale bar, 500 nm. (M) Transection of type I vestibular hair cell almost entirely surrounded by calyx-type synapse (in red). Scale bar, 500 nm. (N) OTOF expression in MYO7A^+^ hair cells. Scale bar, 10 μm. (O) H&E overview with CDH23 in stereocilia by IHC. Scale bars: H&E, 100 μm; IHC, 20 μm. (P) H&E overview with WHRN in stereocilia by IHC. Scale bars: H&E, 100 μm; IHC, 20 μm. H&E and IHC images are representative of n ≥ 6 aggregates (≥2 experiments) (E, F, and N–P) or n ≥ 2 human inner ear tissues of matching fetal age (G and H). TEM images are representative of n R 3.

**Figure 6.**
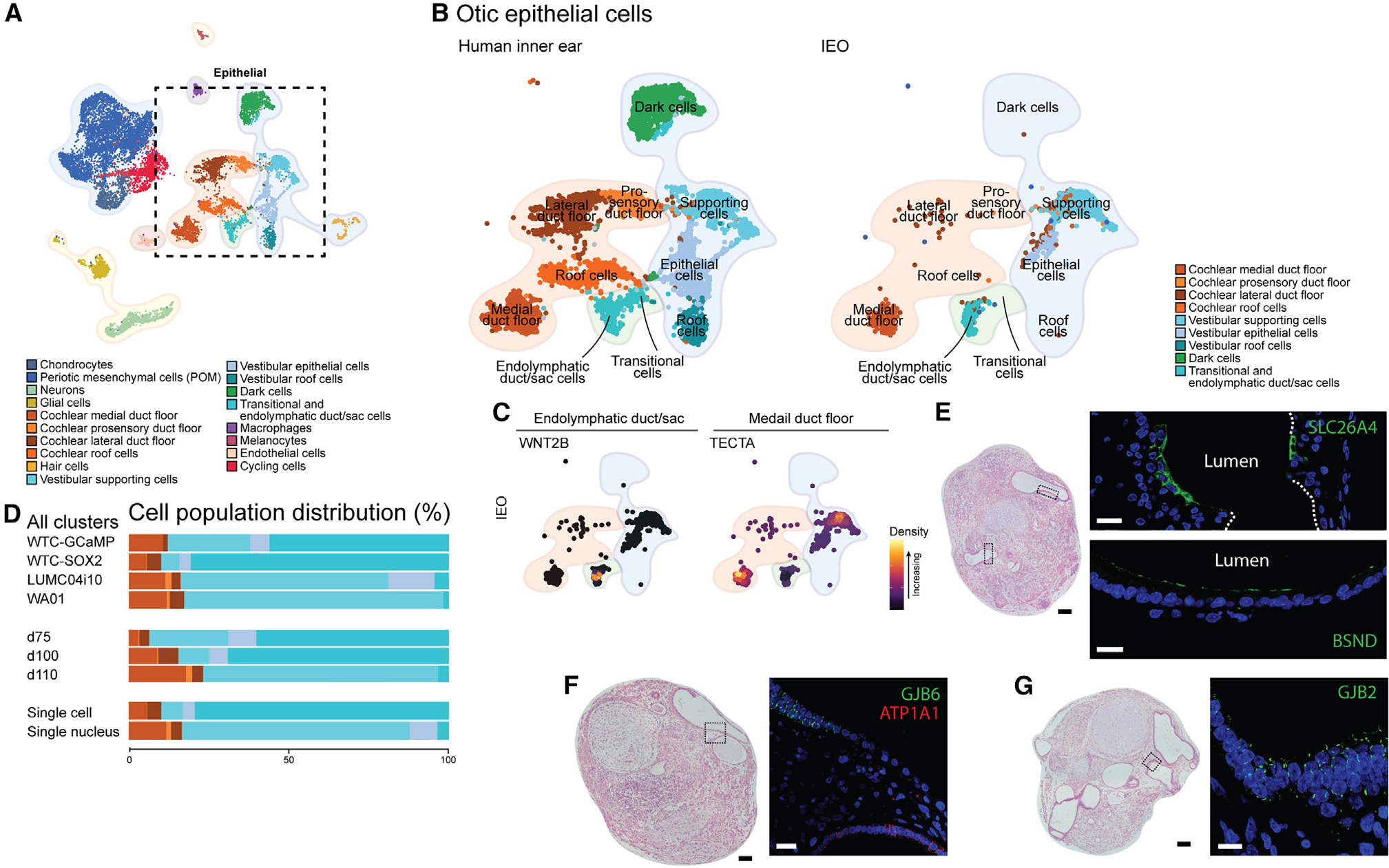
Endolymphatic duct/sac, cochlear epithelia, and vestibular supporting cells in IEOs (A) UMAP plot of annotated human inner ear atlas highlighting epithelial cell types. (B) UMAP plot comparing reference epithelial cell populations (left) and mapped IEO-derived otic epithelium population (right) using Symphony. (C) Density plots of IEO-derived epithelial populations showing expression of *WNT2B* (endolymphatic duct/sac marker) and *TECTA* (medial duct floor marker). (D) Relative contribution of cell types per cell line, time point, and technique used. (E) H&E overview with expression of SLC26A4 and BSND in the highlighted area (D75). Scale bars: H&E, 100 μm; IHC, 20 μm. (F) H&E overview and localized expression of GJB6 and ATP1A1 within the highlighted area (D75 IEO). Scale bars: H&E, 100 μm; IHC, 20 μm. (G) H&E overview and expression of GJB2 within the highlighted epithelium (D75 IEO). Scale bars: H&E, 100 μm; IHC, 20 μm. H&E and IHC images are representative of n ≥ 6 aggregates (≥2 experiments) (E–G).

IEO-derived hair cells were mapped to hair cells of the human inner ear atlas reference using Symphony (Figures 5A and 5B). We do not expect that any cochlear hair cells were captured in the human inner ear atlas, as these arise only by FW10 of fetal development.^66,67^ Within the reference hair cells, fetal developing hair cells clustered separately from the adult hair cells (Figures 5B and 6B). Immature fetal hair cells expressed *SOX2, ATOH1*, and *ANXA4* (Figure 5C). Additionally, two distinct adult hair cell populations were present, showing specific expression for markers of type I and type II vestibular hair cells, *OCM*^68^ and *ANXA4*,^69^ respectively (Figure 5C). The IEO-derived hair cells overlapped with the developing immature hair cells and expressed *SOX2* and *ATOH1* (Figures 5B and 5C).

Although the fetal immature vestibular hair cells cluster together (Figure 5B and S6B), a distinction can be made between developing type I and type II vestibular hair cells at FW9.^54^ Type I and type II vestibular hair cells can be distinguished by their shape and the type of synapse (Figure 5D). We identified calyx-type synapses (TUBB3^+^) in D75 IEOs (Figure 5E), which are typically associated with type I vestibular hair cells. Although we observed these presumptive type I hair cells in IEOs, they did not exhibit the characteristic “neck” structure, which is a hallmark of more mature type I hair cells and develops after birth in mice.^70^ It is possible that the IEO-derived hair cells were still immature at the time of our analysis. Additionally, within IEOs we could identify ANXA4^+^ hair cells, a known marker for developing hair cells and type II vestibular hair cells, with some of these hair cells also expressing OCM, a marker for type I vestibular hair cells (Figure 5F). This expression is similar to the human fetal utricle (Figures 5G and 5H). Additionally, we performed light-microscopical and ultrastructural analyses of the hair cells present in IEOs. The IEOs consisted of an open lumen lined by a layer of columnar cells with small, densely stained nuclei in their basal part (Figure 5I). Interspersed between these epithelial cells are flask-shaped cells with a large, round nucleus and from which a large hair bundle protrudes into the lumen. The morphology of these cells closely resembles that of vestibular hair cells. Using TEM, the flask-like cells contain a large hair bundle, in which the individual stereocilia are of varying length (Figure 5J). These stereocilia are inserted into the cuticular plate in the apical part of the hair cell as is evident by the presence of stereociliary rootlets (Figure 5K). At the opposite pole of these hair cells, frequently seen features were synaptic ribbons which are oriented opposite the bouton-type synapses associated with the basal membranes of the hair cells (Figure 5L). As these synapses did not contain any vesicles it may be surmised that these are afferent neurons. The observation that these cells were associated with bouton-type synapses seemed to indicate that they were type II vestibular hair cells.^18^ Other hair cells, however, resembled the calyx-type synapse seen with type I vestibular hair cells (Figure 5M).

Further analyses revealed that these immature vestibular hair cells express proteins of genes linked to SNHL. In addition to MYO7A (DFNA11, DFNB2, and Usher syndrome type I), OTOF (DFNB9), CDH23 (DFNB12 and Usher syndrome type I), and WHRN (DFNB31) are present within the hair cell or its stereocilia.

### Cochlear and endolymphatic duct/sac epithelium induced in IEOs

On the basis of previous work, the IEOs are assumed to have a vestibular sensory epithelium containing supporting cells.^18^ In line with this hypothesis, most IEO-epithelial populations were mapped to the vestibular supporting cells and epithelial cells, with a total lack of vestibular dark cells and roof cells (Figures 6A and 6B). However, IEO-derived epithelial populations also overlapped with the endolymphatic duct/sac cells, expressing *WNT2B* (Figure 6C). Furthermore, a large overlap is seen with the cochlear medial duct floor, to a lesser degree for the cochlear lateral duct floor, where it is almost absent for the prosensory domain and cochlear roof cells. Marker gene expression of *TECTA* confirmed the medial duct floor-like population within the IEOs (Figure 6C). *TECTA* expression in the vestibular supporting cells may be explained by its temporal expression in the vestibular organs.^71^ The presence of these populations was consistent over cell lines and time points, although in varying degrees (Figure 6D).

Using IHC, we were able to show the presence of SLC26A4 and BSND, similar to the developing human inner ear (Figures 6E and S6E). Mutations in *SLC26A4* may cause SNHL in DFNB4 and Pendred syndrome, and mutations in *BSND* can lead to DFNB73. Within the IEO epithelia we were able to show additional expression of ATP1A1, GJB6, and GJB2 (Figures 6F and 6G). Mutations in *GJB2* are the most common cause of nonsyndromic SNHL causing DFNA3A and DFNB1A. Within epithelia these connexin-coding genes were expressed together with GJB6 that causes DFNA3B. These proteins are *in vivo* expressed in a spatiotemporal manner in the inner ear epithelia, including the human medial cochlear duct floor^47^ and sensory vestibular epithelia.^72^ Moreover, ATP1A1, implicated in age-related hearing loss,^73^ has a spatiotemporal expression during the development of the inner ear,^46,66^ and shows epithelial expression within IEOs (Figure 6F).

### Cell-cell communication analysis highlights interactions between endothelial cells and hair cells

**Figure 7.**
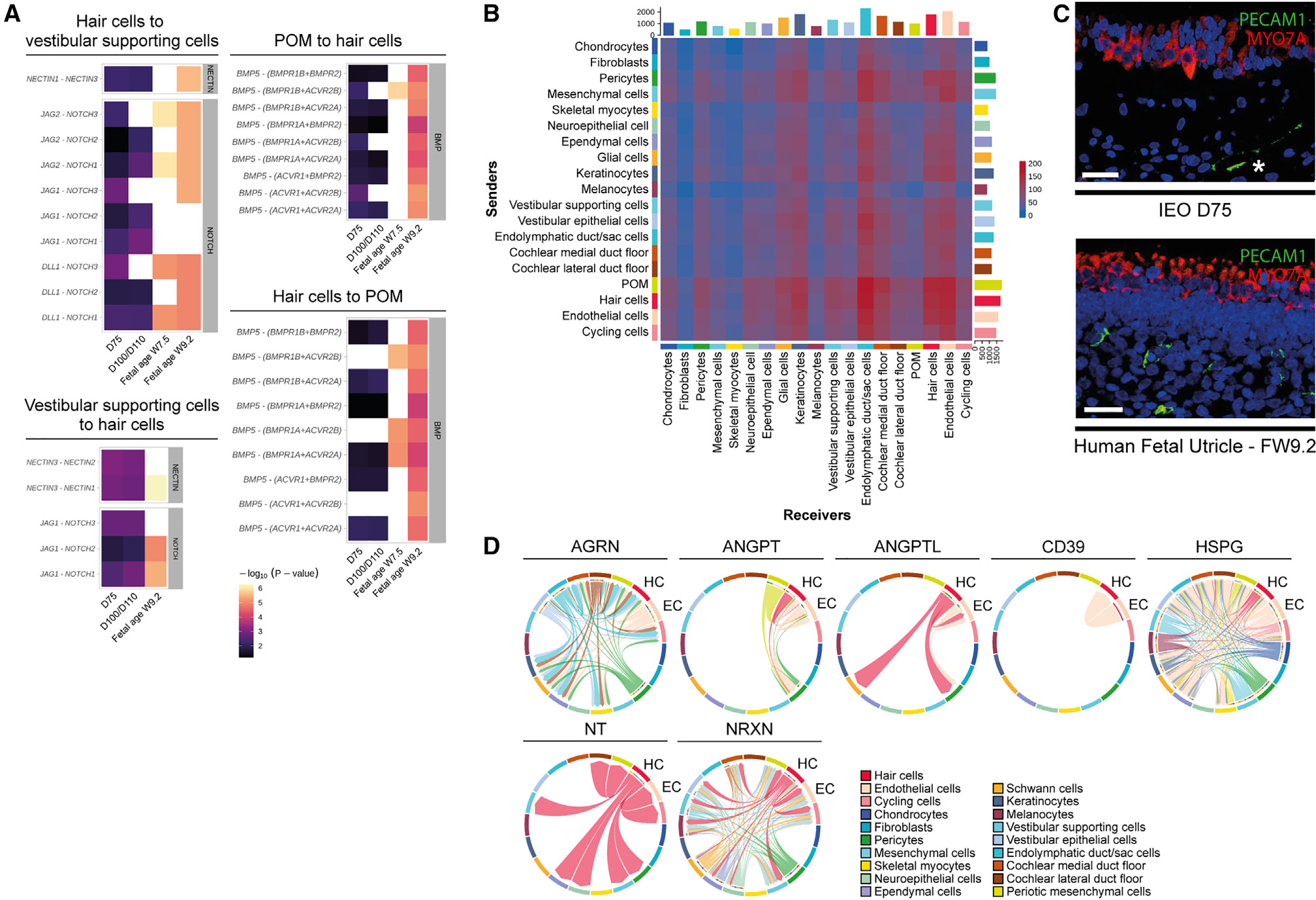
Cell-cell communication analysis of IEOs focusing on hair cells and endothelial cells (A) Ligand-receptor interactions in NECTIN, NOTCH, and BMP signaling pathways. Validated in human inner ear atlas (FW7.5 and/or FW9.2). (B) Heatmap showing total interactions between cell types. x axis, receivers (with receptors); y axis, senders (with ligands). (C) PECAM1^+^ endothelial cells underlying sensory epithelium with MYO7A^+^ hair cells in D75 IEO (upper) and FW9.2 fetal utricle (lower). Images are representative of n ≥ 6 aggregates (≥2 experiments) or n ≥ 2 human inner ear tissue with matching fetal age. Scale bars, 50 μm. (D) Chord diagrams displaying interactions between cell types in IEOs. Arrows indicate sender to receiver cell types. POM, periotic mesenchyme. See also Figure S7 and Table S1.

To better understand interactions between cell types in the IEO, we performed a cell-to-cell communication analysis using Cell-Chat.^74^ This R package computes signaling pathway interactions between cell types using a database of known interactions among ligands, receptors, and cofactors. A total of 223 signaling pathways were analyzed, of which CellChat determined 85 pathways significantly engaged within the IEOs (Table S1). These include signaling pathways that are known to be essential for the inner ear and neurosensory development: BMP, FGF, NOTCH, TGFβ and WNT signaling (Figure S7A).^75–78^ By delving deeper, we identified known ligand-receptor interactions within the IEOs (Figure 7A). For instance, the interaction of *NECTIN3* with *NECTIN1* and/or *NECTIN2* between supporting cells and hair cells contributes to the arrangement of the sensory epithelium.^79^ Also the interactions between *JAG1*, *JAG2*, or *DLL1* with *NOTCH1*, *NOTCH2*, or *NOTCH3* between these same cell types are known mechanisms by which NOTCH signaling contributes to hair cell formation.^80–83^ Additionally, the POM, a crucial component for hair cell differentiation,^84^ demonstrates communication with hair cells and supportive cells, highlighting the role of BMP signaling, in interacting to known receptors within the sensory epithelium.^85^ These ligand-receptor interactions of the NECTIN, NOTCH, and BMP pathways could be validated in the fetal inner ear data (Figure 7A). Within these interactions, we observed consistently higher p values in the fetal inner ear data compared with the IEO data. We hypothesize that this observation may be attributed to the difference in experimental modalities, as the former uses snRNA-seq data while the latter combines snRNA-seq and scRNA-seq. As the CellChat analysis revealed these expected interactions in the IEOs, we were confident that this approach could be used to identify novel interactions. We noted that the vascular endothelial cells showed a high number of interactions with other cell types, including hair cells and the other otic cell types (Figure 7B). By IHC, we found endothelial cells (PECAM1^+^) throughout the IEO aggregates, including locations near in the mesenchyme adjacent to hair cell-containing (MYO7A^+^) epithelia, similar to the developing fetal sensory epithelium (Figure 7C). To evaluate the interactions that might contribute to hair cell development, we focused on the interactions between endothelial cells and hair cells and found 52 relevant signaling pathways (Figure S7B). We subsequently analyzed the ligand-receptor interactions within these signaling pathways and validated these by using the ligand-receptor interactions in the fetal data of the human inner ear atlas (Figures S7C and S7D). These validated interactions include interactions in the AGRN, ANGPT, ANGPTL, CD39, HSPG, NT, and NRXN signaling pathways, suggesting that these play a role in formation of specific cell types in the IEOs, including endothelial cells and hair cells (Figure 7D). The close proximity of endothelial cells to hair cells, the number of interactions between these cell types, as well as the validated ligand-receptor interactions, are indicative for a strong association in proper sensory development.

### IEOs express SNHL genes and proteins

IEOs are valuable for disease modeling, including syndromic and nonsyndromic SNHL which has been associated with more than 150 genes. We examined whether these genes are expressed in the appropriate cell types of the IEO model. Most SNHL genes are linked to specific inner ear cell types.^86,87^ We created a comprehensive summary table of gene/protein expression for 158 genes (Table S2). Using heatmaps and protein expression, we developed a scoring system to determine the suitability of studying a gene of interest using IEOs (Table S2; Figure S8). Sixty-seven of 158 genes showed expression in IEOs in the correct cell populations. Furthermore, because the IEOs display predominantly vestibular-like features, we identified whether a given gene had been linked to vestibular phenotypes. With modified induction techniques, the list of target genes in IEOs is expected to expand in future studies, including cochlear supporting cells and hair cells. Together, our data provide a powerful resource for the community to design and plan SNHL modeling studies using IEOs.

## DISCUSSION

In this study, we differentiate multiple hPSC lines to induce inner ear cell types and used single-cell transcriptomics to evaluate aggregates up to 110 days in culture. We also created a human inner ear single-cell atlas containing data from fetal and adult inner ear tissue. Our data show that differentiation efficiency of IEOs is largely dependent on the initial BMP-4 concentration and can be determined by epithelial thickness at D3. The change of this epithelial thickness following BMP-4 treatment resembles the ectoderm thickness at the gastrula stage which is controlled by BMP activity (Figure S1B).^76,88,89^ Using efficiently differentiated aggregates, we discovered the presences of additional off-target cell types (cranial skeletal myocytes, ependymal cells, vascular endothelial cells) and show that most of the cell types have fetal gene expression signatures. These cell types could potentially be used by adaption of the current protocol, similar to the generation of hair-bearing skin organoids.^38^ Likewise, the on-target formation of otic epithelium and POM within the IEOs show overlap with the fetal transcriptomic data.

Using the human inner ear atlas, we determined that IEOs primarily consist of vestibular hair cells, vestibular supporting and epithelial cells, and a relatively smaller contribution of endolymphatic duct/sac cells and cochlear cell types. Interestingly, other nonsensory and cochlear prosensory cells are lacking, indicating that specific inner ear cell types are induced. The vestibular epithelia and endolymphatic duct/sac cells are derived from the dorsal otic vesicle, with the formation of vestibulocochlear ganglion-like neurons and cochlear epithelium from the anterior-ventral part of the otic vesicle.^76,90,91^ Although cochlear-like epithelium is present in the IEOs, the prosensory development is lacking, possibly because of insufficient BMP activity^63^ which is not required for proper vestibular sensory formation.^60^ Manipulating BMP and retinoic acid, to induce nonsensory epithelia,^92^ may lead to more patterned IEOs with a clinically relevant cell type heterogeneity. Additionally, the IEOs up to D110 resemble the human inner ear at approximately FW9: displaying immature vestibular hair cells, development of specific cochlear domains and vestibular cell types, and the presence of melanocytes surrounding the otic epithelia.^46^ Using the endothelial population and media flow could enhance maturation similar to kidney organoids.^93^ Alternatively, prolonged (>D110) cell cultures in which off-target induction is addressed, could result in a more mature phenotype. Currently, off-target cell types pose a challenge for their use in pre-clinical studies, as it hampers prolonged culture and inhibits inner ear epithelia maintenance.

In addition to the IEO single-cell data, we present the human inner ear atlas containing vestibular and cochlear tissue from the first trimester as well as adult vestibular tissue. No single-cell reference of the human inner ear was available, as recent work using the transcriptomics approaches is focused on mouse inner ears^24,27,55,94,95^ or limited to human cochlear tissues.^28^ Future research efforts aim to expand on the human inner ear atlas in order to generate additional resources for understanding inner ear development and interrogating development in the context of genetic SNHL. Current knowledge of these pathophysiological mechanisms is often missing, biased toward cochlear neurosensory epithelium, and disregards the spatiotemporal expression of genes during inner ear development. Additionally, there is increasing awareness for vestibular dysfunction associated with SNHL.^96^ This paper motivates the importance of human inner ear atlases containing the whole inner ear throughout different stages of development.

Using both the IEO and human inner ear single-cell data, we observed known cell-cell interactions within the IEOs that contribute to neurosensory development. Additional computed interactions highlighted the potential role of endothelial cells in hair cell development. Endothelial cells have been implicated in neurosensory development in zebrafish^97^ and influence organoid formation of kidney and intestine.^93,98,99^ We identified signaling pathways, such as AGRN and NT signaling, that are involved in otic vesicle development^100–103^ and hair cell development,^104,105^ respectively. However, their interaction with endothelial cells in the inner ear has not been previously described. For other signaling pathways (ANGPT, ANGPTL, CD39, HSPG, and NRXN), limited literature exists on their involvement in hair cells,^106–110^ but these have functions in endothelial homeostasis, blood-brain barrier function, or renal glomerular integrity.^111–117^ One might surmise their interaction in inner ear endothelial integrity with the interaction between hair cells and endothelial cells being important for their development and subsequent function. These results show how IEOs are a promising model system to further investigate developmental inner ear signaling pathways.

Although diversity or maturity of the human inner ear is not fully captured, IEOs could potentially be used for disease modeling. Based on protein and gene expression, a promising SNHL cause to model is, for instance, congenital Usher syndrome in which the function of hair cells, in both the cochlea and vestibular organs is affected. Within the epithelium also DFNA3A and DFNB1A (*GJB2*) as well as DFNA3B (*GJB6*) can be modeled. Additionally, DFNA9 (*COCH*), DFNA40 (*CRYM*), and DFNX2 (*POU3F4*), all of which affect the function of POM, can potentially be modeled within the IEOs. These are examples of numerous other gene targets that could be evaluated in IEOs. The ability to use the IEOs for disease modeling provides a broadly applicable framework to develop cell or gene therapies for genetic syndromic and nonsyndromic SNHL.

### Limitations of the study

There are several limitations to our study. First, we could not perform a thorough analysis of cell line differences or batch-to-batch analysis, because of the methods used to analyze the IEOs (scRNA-seq or snRNA-seq). Second, minor cell populations might not be detected in samples of <10,000 cells or nuclei captured per experiment. Third, merging the single-cell and single-nucleus data required correction for mitochondrial and ribosomal content between methods. Furthermore, our human inner ear atlas is based on a limited number of samples, and further developmental stages need to be included to create a more comprehensive and accurate atlas. However, our integration approach can be used as new datasets are published. Additionally, different expression patterns between the single-cell and single-nucleus data were observed when analyzing gene expression. Last, because of shallow sequencing depth, we could not provide a thorough insight into transcription factors as cell identity drivers. Future datasets combining single-cell transcriptomics with spatial biology or chromatin state would allow more insight into inner ear development. We encourage fellow researchers to use our dataset and human inner ear atlas for additional discoveries.

## STAR★METHODS

### RESOURCE AVAILABILITY

#### Lead contact

Further information and requests for resources and reagents should be directed to and will be fulfilled by the lead contact, Wouter H. van der Valk (w.h.van_der_valk@lumc.nl).

#### Materials availability

This study did not generate new unique reagents.

#### Data and code availability

- Single-cell and single-nucleus RNA-seq data have been deposited the Gene Expression Omnibus and are publicly available as of the date of publication. Accession numbers are listed in the key resources table. The data can also be accessed through a data exploration platform hosted by the Expression Analysis Resource,^124^ available at https://umgear.org/p?l=hIEOandInnerEar.
- This paper does not report original code. Scripts used for scRNAseq and snRNAseq of IEOs analysis are available at https://github.com/Koehler-Lab/, scripts used for the human inner ear atlas are available at https://github.com/OtoBiologyLeiden/.
- Any additional information required to reanalyze data reported in this paper is available from the lead contact upon request.

### EXPERIMENTAL MODEL AND STUDY PARTICIPANT DETAILS

#### Pluripotent stem cell lines

Experiments were performed with eight apparently healthy hPSC lines. The WA01 human embryonic stem cell line (male, ethnicity unknown, passages 47–52) was purchased from WiCell Research Institute, provided by Dr. James Thomson at the University of Wisconsin. The two commercially available hiPSC lines that were used are from the same parental male background line (ethnicity Japanese, GM25256 from the Coriell Institute): AICS-0074 with the SRY-Box Transcription Factor 2-mEGFP (SOX2-GFP) reporter (hereafter WTC-SOX2, passages 34–46) acquired from the Allen Institute and WTC-GCaMP with a version of the genetically engineered calcium indicator (passages 36–44) provided by Bruce Conklin at the Gladstone Institutes/UCSF.^125^ We used additional hiPSC lines that were generated in-house. Generated by the Human iPSC Hotel (Leiden University Medical Center) are LUMC0004iCtrl10 (hereafter LUMC04i10, male, ethnicity unknown, passages 15–36)^126^ and LUMC0044iCtrl44 (hereafter LUMC44i44, female, ethnicity unknown, passages 20–43).^127^ Generated by the Human Neuron Core (Boston Children’s Hospital) are SAH0047–02 (female, ethnicity unknown, passages 23–25),^128^ GON0515–03 (male, ethnicity unknown, passages 22–24)^129^ and GON0926–02 (female, ethnicity unknown, passages 19–21). Cell lines were evaluated for pluripotency, normal karyotype, and tested negative for mycoplasma prior to experimentation. Cells were cultured on 6-well plates coated with vitronectin recombinant human protein (A14700, Gibco, concentration 0.5 μg/cm) and maintained in Essential 8 Flex medium (A2858501, Gibco) with Normocin (ant-nr-1, Invivogen, concentration 100 μg/mL), hereafter E8 medium. The E8 medium was replenished every other day or every day depending on cell confluency. At approximately 80% confluence (every 4–5 days on average), WTC-SOX2 cells were passaged as single cells using Stempro Accutase Cell Dissociation Reagent (hereafter Accutase; A1110501, Gibco) using 10 mM Y27632 (04–0012-02, Stemgent). The Y27632 was removed within 24 h after passaging by a medium replenishment. All other lines were passaged at approximately 80% confluency (every 4–5 days on average) as tiny clusters (3–5 cells on average) using 0.5 mM EDTA (15575020, Gibco, concentration 10 mM).

#### Human inner ear tissue

Collection of human fetal inner ear tissue was performed according to the Dutch legislation (Fetal Tissue Act, 2001) and the WMA Declaration of Helsinki guidelines. For this project, we obtained approval from the Medical Research Ethics Committee of Leiden University Medical Center (protocol registration number B19.070) as well as written informed consent of the donor following the Guidelines on the Provision of Fetal Tissue set by the Dutch Ministry of Health, Welfare, and Sport (revised version, 2018). Apparently healthy inner ear tissue was collected after elective termination of pregnancy by vacuum aspiration as previously described.^130^ Embryonic or fetal age was determined by obstetric ultrasonography prior to termination, i.e., gestational age minus two weeks, with a standard error of two days. No other characteristics were documented in line with legislation and medical ethical approval. Intact inner ears were either stored in RNAlater (AM7020, Invitrogen) when processed for snRNAseq experiments or processed as previously described.^66^ In summary, inner ears were obtained from vacuum-aspirated tissue collected in phosphate-buffered saline (PBS) with a pH of 7.4. The tissue was then transferred to 4% formaldehyde, prepared from paraformaldehyde, in 0.1 M Na+/K + -phosphate buffer with a pH of 7.4, and fixed for at least one night at 4°C.

Adult vestibular tissue from a 47-year-old male was collected during translabyrinthine vestibular schwannoma surgery in PBS with a pH of 7.4 similar to previous work by our group.^130^ No other characteristics were documented in line with legislation and medical ethical approval. Ampullae and the utricle were obtained separately and evaluated under a dissection microscope before being experimentally processed. This project was approved by the Medical Research Ethics Committee of Leiden University Medical Center (protocol registration number B18.028) and tissue was obtained after written informed consent of the donor.

### METHOD DETAILS

#### BMP-4 preparation

BMP-4 from different vendors were used for these experiments. BMP-4 from Peprotech (120–05) was reconstituted in sterile 5 mM citric acid containing 0.2% (wt/vol) of human serum albumin to a concentration of 100 μg/mL. BMP-4 from Stemgent (03–0007) and R&D Systems (314-BPE) were reconstituted in sterile 4 mM HCl to a concentration of 100 μg/mL. BMP-4 from R&D Systems (314-BP) was reconstituted in sterile 4 mM HCl containing 0.1% (wt/vol) human serum albumin to a concentration of 100 μg/mL. Reconstituted BMP-4 was stored as 2 μL aliquots at −80°C and replaced every six months.

#### Differentiation of hPSCs

Inner ear organoid induction followed the protocol recently published with minor alterations.^18,21^ In brief, colonies of human pluripotent stem cells were detached and dissociated using Accutase and collected as a single-cell suspension in E8 medium containing 20 μM Y27632. 3,500 cells in 100 μL suspension were distributed per well of 96-well U-bottom plates with a super-low cell attachment surface (174925, Thermo Scientific). After centrifugation at 110×*g* for 6 min to aid aggregation, the cell aggregates were incubated in a 37°C incubator under 5% CO_2_ for 48 h. After 24 h, 100 μL of fresh E8 medium was added per well to dilute out Y27632.

At differentiation day 0 (D0), all cell aggregates were collected and individually transferred to a new 96-well U-bottom plate with a super-low cell attachment surface in 100 μL of E6 medium (A1516401, Gibco) with Normocin (concentration 100 μg/mL), hereafter E6 medium, containing 2% Matrigel Growth Factor Reduced (hereafter Matrigel; 354230, Corning), 10 μM SB431542 (04–0010-05, Stemgent), 4 ng/mL basic FGF (hereafter bFGF; 100–18B, PeproTech) and 0–40 ng/mL BMP-4 (listed in the key resources table). On D3, 25 μL of E6 per well was added in with an end-concentration of 200 ng/mL LDN (04–0074-02, Stemgent) and 50 μg/mL bFGF, making the total volume 125 μL per well. This treatment induces cranial neural crest formation. On D6, the total volume is increased to 200 μL by adding 75 μL of fresh E6 medium. On D8, 100 μL of the medium was changed for E6 containing 6 μM CHIR99021 (hereafter CHIR; 04–0004-02, Stemgent) to induce otic placode formation. On D10, 100 μL of the medium was changed for fresh E6 containing 3 μM CHIR.

On D12, the aggregates were transferred to Organoid Maturation Medium (OMM) containing 1% Matrigel and 3 μM CHIR in a 24-well plate with a super-low cell attachment surface (174930, Thermo Scientific) on an in-incubator orbital shaker. OMM is a 1:1 mixture of advanced DMEM:F12 (12634010, Gibco) and Neurobasal Medium (21103049, Gibco), supplemented with 0.5x B27 without vitamin A (12587010, Gibco), 0.5x N2 Supplement (17502048, Gibco), 1x Glutamax (35050061, Gibco), 0.1 mM β-mercaptoethanol (21985023, Gibco), and Normocin. On D15, a half medium change was performed with OMM containing 1% Matrigel and 3 μM CHIR. On D18, a half medium change with OMM was performed, diluting out the Matrigel and CHIR. On D21, a full medium change with OMM was performed. Half of the medium was replenished every 3 days, with a full medium change every 9 days. The volume was gradually increased starting from 500 μL at D12, to 1 mL on approximately D45, to 1.5 mL on approximately D60 and later.

#### *In vitro* measurements of aggregate morphology

Aggregates were monitored during the differentiation period with a Nikon TS2R microscope or an EVOS M5000 microscope using either bright-field or phase-contrast visualization. Images collected were analyzed using ImageJ measuring epithelial thickness at three different locations, circularity, and area.

#### Vibratome-slicing of aggregates

Aggregates were embedded in low 2% low melting point agarose (16520050, Invitrogen) and 200 mm thick slices were obtained using a motorized vibratome (VT1200, Leica Microsystems). Sections were obtained in ice-cold PBS (14190144, Gibco) and bright-field evaluation was performed using a Nikon TS2R microscope. Selected sections were processed for scRNAseq as described below.

#### Aggregate dissociation to single cells for scRNAseq

Vibratome sections of randomly selected aggregates were pooled at specified time points. Three to four number of aggregates were placed in warm TrypLE solution (12605036, Gibco) and incubated in a 37°C incubator under 5% CO_2_ on an orbital shaker set to 65 rpm. The mixture was triturated with a pipette using wide bore p1000 tips every 10 min. Dissociation to single cells was complete after 60 min and confirmed by bright-field visualization using a Nikon TS2R. TrypLE activity was stopped by adding cold 3% bovine serum albumin (BSA; A7030, Sigma-Aldrich) in PBS. To remove residual cell aggregates and debris, the suspension was filtered with a 40 μm Flowmi cell strainer (H13680–0040, Bel-Art). After washing three times with 3% BSA in PBS, cells were resuspended in ice-cold 3% BSA in PBS and filtered again with a 40 μm Flowmi cell strainer. Cell viability was determined by using Trypan Blue (15250061, Gibco) and an automated cell counter (Countess II, Thermo Fisher). Cell suspensions were diluted with cold 3% BSA in PBS to reach a final concentration of 1,000 cells/μl.

#### Aggregate dissociation to single nuclei for snRNAseq

Two to four randomly selected aggregates were processed at specified time points. Aggregates were pooled, washed once with ice-cold PBS, and resuspended in lysis buffer that contained 10 mM Tris-HCl (T2194, Millipore-Sigma), 10 mM NaCl (59222C, Millipore-Sigma), 3 mM MgCl_2_ (M1028, Millipore-Sigma), 0.1% Nonidet P40 Substitute (74385, Millipore-Sigma) in DEPC-treated water (750024, Invitrogen). The suspension was transferred to a Dounce tissue grinder (885300–0002, Kimble) and ground every 5 min. After 20 min, dissociation to single nuclei was confirmed by bright-field visualization (EVOS M5000, Thermo Scientific). After trituration using p1000 and p200 tips, the suspension was filtered through a 70 mm MACS SmartStrainer (130–098-462, Miltenyi Biotec), centrifuged at 500×*g* for 5 min at 4°C and resuspended in 1% BSA in PBS (AM2616, Invitrogen) containing 200 U/μl RNase inhibitor (3335402001, Millipore-Sigma). After filtration using a 40 μm Flowmi cell strainer and centrifugation at 500g for 5 min at 4°C, the nuclei suspension was resuspended to a final concentration of 1,000 cells/μL.

For one experiment of D110 aggregates generated using the LUMC04i10 hiPSC line, we performed nuclear hashtagging of two separate aggregates using single nuclei hashing antibodies (MAb414, Merck). To this end, the nuclei suspension was blocked using Fc-blocking reagent (422302, Biolegend) for 5 min on ice followed by incubation with the hashing antibody (1 μg per 100 μL) for 10 min on ice. Subsequently, nuclei were washed three times using 1% BSA in PBS containing 200 U/μl RNase inhibitor, pooled together 1:1, and resuspended to a final concentration of 1,000 cells/μl.

#### Human inner ear tissue dissociation for snRNAseq

Human fetal inner ear tissue was stored in RNAlater at 4°C and processed to a single nuclei suspension within 16 h after collection. The adult human tissue, comprising one ampulla (from the anterior or lateral semicircular canal) and one utricle, was processed within 20 min of collection. The inner ear tissue was assessed under a dissection microscope (M205C, Leica). As for the fetal inner ears, residual tissue was removed using the same microscope. The FW7.5 fetal inner ear was processed with the otic capsule intact, while the otic capsule was manually removed for the FW9.2 fetal inner ear tissue. For the FW9.2 specimen, our dissection strategy resulted in isolating the membranous labyrinth of the cochlea and vestibular system, which was then processed for nuclei isolation. The human inner ear tissue followed a similar protocol of the aggregate dissociation to single nuclei as described above. Minor alterations include the lysis buffer that contains 0.005% Nonidet P40 substitute and additional filtration steps following the 40 μm Flowmi cell strainer: the suspension was filtered through a 20 μm and 10 μm Pluristrainer (43–10020-40 and 43–10010-40, PluriSelect) to remove debris.

#### RNA-seq cDNA library preparation and sequencing

Single cell and nucleus gene expression libraries were generated on the 10x Genomics Chromium platform using the Chromium Next GEM Single Cell 3′ Library & Gel Bead Kit v2 (scRNAseq) or v3.1 (snRNAseq) and Chromium Next GEM Chip G Single Cell Kit (10x Genomics) according to the manufacturer’s protocol. Hashtag libraries were amplified and barcoded as in Stoeckius et al.^131^ Gene expression and hashtag libraries were sequenced on a NextSeq500 or NovaSeq 6000 S4 flow cell using v1 Chemistry (Illumina) and FASTQ files were generated using Cell Ranger mkfastq (10x Genomics).

#### Analysis of scRNAseq and snRNAseq data

The 10x Genomics Cell Ranger 6.1.0 pipeline (http://support.10xgenomics.com/) was used to generate demultiplexed FASTQ files from the raw sequencing reads. The reads were aligned to the human reference GRCh38 genome. For snRNAseq reads, reads were mapped to introns as wells as exons, resulting in a greater number of genes detected per nucleus. Levels of gene expression were analyzed using the number of UMIs (unique molecular indices) detected in each cell. Cell Ranger was used to generate filtered gene expression matrices.

Low-quality cells were removed by quality control on UMI number, number of genes, and percentage of transcripts aligned to mitochondrial genes as well as ribosomal genes. The parameters used were different between the single-cell and single-nucleus data. Cell line-unique single nucleotide polymorphisms (SNPs) were used to demultiplex the single-nucleus data, by which we could assign a cell line identity to 97.3% of the nuclei. Using Seurat,^118^ and after normalization and scaling of the data, identification of variable features was performed using SCTransform^121^ regressing out the effect of cell cycle, mitochondrial content and/or ribosomal content. We computed PCA using selected number of highly variable genes. Harmony correction was performed to integrate the datasets into a uniform low-dimensional representation.^132^ These integrated datasets were used to visualize clustering by Uniform Manifold Approximation and Projection (UMAP) plotting techniques. Cell identity assignation was performed using differential expression analysis and manually by using cell type-specific marker genes.

R packages ggplot2^119^ and Nebulosa^120^ were used to plot quantitative gene expression and specific gene expression patterns. Symphony was used to map the integrated IEO scRNAseq and snRNAseq data to the human inner ear atlas.^65^ Cell-to-cell communication analyses were performed through CellChat (version 1.4.0) and the provided human ligands and receptor pairs database. We computed the probability of communication at the ligand-receptor pair and pathway levels as described in the original manuscript.^74^ Communication between cell types observed in less than 10 cells and with adjusted p-values greater than 0.1 were removed.

#### Aggregate processing and immunohistochemistry

Samples were washed twice with PBS and fixed using 4% formaldehyde (1.04005.1000, Merck) in 0.1 M Na^+^/K^+^-phosphate buffer (pH 7.4) overnight at 4°C. Fixed samples were either paraffin embedded or processed for cryosectioning. For paraffin embedding, aggregates were dehydrated in an ascending ethanol (70%–99%; 84050068.2500, Boom) series, cleared in xylene (534056, Honeywell), and embedded in paraffin wax (2079, Klinipath). Sections of 4–5 μm were cut using a rotary microtome (HM355S, Thermo Scientific). Sections were deparaffinized in xylene and rehydrated in a descending series of ethanol (96%–50%) and several rinses in deionized water. At approximately every 10 sections, one section was selected for routine staining with hematoxylin (40859001, Klinipath) and eosin (40829002, Klinipath). Before immunostaining, antigen unmasking was performed either in 10 mM sodium citrate buffer (pH 6.0; S1804–500G, Sigma-Aldrich) for 12 min at 97°C. For cryosectioning, aggregates were cryoprotected with graded treatments of 15% and 30% sucrose solutions in PBS and embedded in tissue-freezing medium (1518313, General Data Healthcare) using cryomolds (25608, Andwin Scientific). Cryosections of 12 μm were cut using a cryostat (CM-1860, Leica Microsystems).

For immunostaining, sections were rinsed in washing buffer consisting of 0.05% Tween-20 (H5152, Promega) and incubated for 30 min with blocking solution consisting of 5% bovine serum albumin (BSA; A7030, Sigma-Aldrich) and 0.05% Tween-20 in PBS, followed by overnight incubation at 4°C with the primary antibodies listed in the key resources table. Next, sections were incubated at ambient temperature (RT) with the Alexa Fluor-conjugated secondary antibodies for 2 h, listed in the key resources table. Nuclei were stained with 4′,6-diamidino-2-phenylindole (DAPI; 1:1,000; D1306, Invitrogen). Sections were mounted with ProLong Gold Antifade Mountant (P36934, Invitrogen). Negative controls were carried out by matching isotype controls and/or omitting primary antibodies. Positive controls were carried out by staining sections of known positive human tissue samples based upon their protein expression profiles, including human inner ear tissue. At least two separate immunostaining experiments were performed with each primary antibody.

#### Human fetal inner ear tissue processing and immunohistochemistry

Fixed fetal inner ear tissue was dehydrated in an ascending series of ethanol (70%–99%), cleared in xylene, and embedded in paraffin wax. After sectioning, deparaffinization, and rehydration as previously outlined, HE staining was conducted on approximately every 10th slide for quality assessment and identification of inner ear morphology and specific inner ear structures.

#### Sample processing for transmission electron microscopy

At D74, LUMC04i10 IEOs (n = 6) were collected and fixed overnight with 2.5% glutaraldehyde in 0.1 M sodium cacodylate buffer (pH 7.4) at 4°C. Fixed specimens were rinsed (3 × 20 min) in 0.1 M sodium cacodylate buffer (pH 7.4) at RT prior to post-fixation in a solution containing 1% OsO_4_ and 1% K_4_Ru(CN)_6_ (potassium hexacyanoruthenate (II) hydrate) in 0.1 M sodium cacodylate buffer (pH 7.4) for 2 h at 4°C followed by several washes in distilled water. Dehydration was performed in an ascending ethanol series (70%−100) and 100% 1,2-propylene oxide followed by infiltration with a mixture of Spurr’s low-viscosity resin and 1,2-propylene oxide (1:1) for 1 h and fresh pure resin under continuous agitation for 2 h at RT. Finally, each specimen was separately embedded in fresh pure resin in a polyethylene embedding mold and polymerized overnight at 70°C.

#### Light microscopy

For light-microscopical assessment, semi-thin (1 μm) plastic sections were cut with a diamond knife (Histo, DiATOME) on a microtome (RM2265, Leica Microsystems), collected on aminoalkylsilane-coated glass slides, stained with a warm aqueous solution containing 1% methylene blue, 1% azure II and 1% sodium tetraborate, and mounted in Entellan (1079610100, Merck) under a glass coverslip.

#### Image acquisition and processing

Sections stained with either HE or methylene blue-azure II were examined with a Leica DM5500B microscope equipped with a Leica DFC450C digital color camera. Digital images were acquired and stored using Leica Application Suite software (LAS version 4.5; Leica Microsystems GmbH). Images were adjusted for optimal contrast and brightness and assembled into figures using Adobe Illustrator 2021 or Adobe Photoshop 2021 (Adobe Systems Software Ltd).

Immunostained sections were acquired with a Leica SP8 confocal laser scanning microscope using Leica objectives (20×/0.7 dry HC PL Apo, 40×/1.3 oil HC PL Apo CS2, 63×/1.4 oil HC PL Apo or 100×/1.3 oil HC PL Fluotar), operating under Leica Application Suite X microscope software (LAS X, Leica Microsystems). Maximal projections were obtained from image stacks with optimized z-step size. Brightness and contrast adjustments were performed with Fiji (ImageJ version 1.52p).

#### Transmission electron microscopy

For ultrastructural examination, representative or interesting areas were selected based on light-microscopical assessment. Ultrathin (60–90 nm) sections were cut with a diamond knife (Ultra 45°, DiATOME) on an Ultrotome III (LKB) and collected upon Pioloform-coated, single-slot copper grids. The sections were contrast-stained with 7% uranyl acetate in 70% methanol and Reynolds’ lead citrate solution and examined in a FEI Tecnai 12 transmission electron microscope (operating at 80 kV) equipped with a Veleta 4-megapixel side-mount CCD camera (Olympus Soft Imaging Solutions GmbH).

Digital images were acquired with TIA software (TEM Imaging & Analysis, version 4.7 SP3; FEI). Alternatively, sets of serial digital images with 10% overlap (at an original magnification of ×30,000) were acquired with the automated EM data acquisition software package SerialEM^133^ (https://bio3d.colorado.edu/SerialEM). Final alignment (stitching) of the sets of overlapping images and montage blending were done using the reconstruction and modeling software package IMOD^123^ (https://bio3d.colorado.edu/imod) operating under the Unix toolkit Cygwin. Montages or single images were finally saved as TIFF and imported into Fiji ImageJ for further image processing, examination and selection of interesting areas. Images were assembled into figures using Adobe Photoshop.

### QUANTIFICATION AND STATISTICAL ANALYSIS

For analyzing the *in vitro* measurements of aggregate morphology, statistical significance was analyzed with two-way ANOVA with a Sidak correction for multiple comparisons. Data was considered statistically significant if p < 0.05.

The immunohistochemical images are representative for inner ear tissue obtained from individual samples of the same fetal week, limited to a minimum of 2 replicates due to the paucity of the tissue. Immunohistochemical images of the inner ear organoids are representative for at least 2 individual experiments with n = 6–10 aggregates per experiment. Electron microscopical images are representative for at least n = 3 aggregates.

## Supplementary Material

1

2

3

## Figures and Tables

**KEY RESOURCES TABLE:** 

REAGENT or RESOURCE	SOURCE	IDENTIFIER

Antibodies		

Mouse monoclonal anti-ACTC1 (1:50)	R&D systems	Cat#: MAB9308-100
Mouse monoclonal anti-ANXA4 (1:200)	Sigma-Aldrich	Cat#: SAB4200121; RRID: AB_10621934
Mouse monoclonal anti-ATP1A1 (1:200)	Novus Biologicals	Cat#: NB300-146; RRID: AB_2060981
Rabbit polyclonal anti-BSND (1:750)	Novus Biologicals	Cat#: NBP2-49101
Mouse monoclonal anti-CDH1 (1:50)	BD Transduction Laboratories	Cat#: 610182; RRID: AB_397581
Rabbit polyclonal anti-CDH23 (1:50)	Invitrogen	Cat#: PA5-53564; RRID: AB_2639592
Rabbit polyclonal anit-DACH1 (1:50)	Invitrogen	Cat#: PA5-52968; RRID: AB_2640365
Mouse monoclonal anti-GATA3 (1:500)	BD Transduction Laboratories	Cat#: 558686; RRID: AB_2108590
Rabbit polyclonal anti-GJB2 (1:100)	Alomone Labs	Cat#: ACC-212; RRID: AB_11124274
Rabbit polyclonal anti-GJB6 (1:100)	Invitrogen	Cat#: PA5-11640; RRID: AB_2111053
Rabbit monoclonal anti-LGR6 (1:100)	Abcam	Cat#: ab126747; RRID: AB_11132458
Mouse monoclonal anti-MLANA (1:50)	Abcam	Cat#: ab731; RRID: AB_305836
Rabbit polyclonal anti-MYO7A (1:100)	Proteus Biosciences	Cat#: 25-6790; RRID: AB_10015251
Rabbit monoclonal anti-MYO7A (1:50)	Novus Biologicals	Cat#: NBP1-84266; RRID: AB_11016173
Mouse monoclonal anti-MYO7A (1:30)	DSHB	Cat#: 138-1s; RRID: AB_2282417
Mouse monoclonal anti-MYOG (1:10)	DSHB	Cat#: F5D; RRID: AB_2146602
Rabbit polyclonal anti-OC90 (1:100)	Invitrogen	Cat#: PA5-71564; RRID: AB_2717418
Rabbit polyclonal anti-OCM (1:200)	Abcam	Cat#: ab150947
Rabbit polyclonal anti-OTOF (1:100)	Novus Biologicals	Cat#: NBP1-46574; RRID: AB_10009850
Rabbit polyclonal anti-OTOR (1:100)	Invitrogen	Cat#: PA5-55053; RRID: AB_2645111
Goat polyclonal anti-OTX2 (1:20)	R&D systems	Cat#: AF1979; RRID: AB_2157172
Goat polyclonal anti-PDGFRB (1:15)	R&D systems	Cat#: AF385; RRID: AB_355339
Mouse monoclonal anti-PECAM1 (1:50)	Dako	Cat#: M082329-2
Alexa Fluor^™^ 568 phalloidin	Invitrogen	Cat#: A12380
Chicken polyclonal anti-POU3F4 (1:10000)	Aves Labs	Cat#: AB_2814704; RRID: AB_2814704
Rabbit polyclonal anti-SIX1 (1:100)	Invitrogen	Cat#: PA5-51654; RRID: AB_2647319
Rabbit polyclonal anti-SLC26A4 (1:100)	Novus Biologicals	Cat#: NBP1-85237; RRID: AB_11032075
Mouse monoclonal anti-SOX2 (1:100)	BD Transduction Laboratories	Cat#: 561469; RRID: AB_10694256
Goat polyclonal anti-SOX10 (1:50)	Invitrogen	Cat#: PA5-47001; RRID: AB_2608449
Rabbit polyclonal anti-TECTA (1:200)	Invitrogen	Cat#: PA5-80102; RRID: AB_2747217
Rabbit monoclonal anti-TFAP2A (1:50)	Abcam	Cat#: ab108311; RRID: AB_10861200
Rabbit polyclonal anti-TNNT1 (1:500)	R&D systems	Cat#: NBP2-38855
Rabbit monoclonal anti-TTR (1:50)	Invitrogen	Cat#: MA5-32634; RRID: AB_2809911
Mouse monoclonal anti-TUBB3A (1:100)	Biolegend	Cat#: 801202; RRID: AB_10063408
Mouse monoclonal anti-TUBB3A (1:100)	R&D Systems	Cat#: MAB1195; RRID: AB_357520
Mouse monoclonal anti-WRHN (1:50)	Abnova	Cat#: H00025861-M05; RRID: AB_10629381
Donkey anti-mouse Alexa Fluor^™^ 488 (1:1000–2000)	Invitrogen	Cat#: A21202; RRID: AB_141607
Donkey anti-mouse Alexa Fluor^™^ 568 (1:1000–2000)	Invitrogen	Cat#: A10037; RRID: AB_2534013
Donkey anti-mouse Alexa Fluor^™^ 594 (1:1000–2000)	Invitrogen	Cat#: A21203; RRID: AB_141633
Donkey anti-mouse Alexa Fluor^™^ 647 (1:1000–2000)	Invitrogen	Cat#: A31571; RRID: AB_162542
Donkey anti-mouse Alexa Fluor^™^ 680 (1:1000–2000)	Invitrogen	Cat#: A10038; RRID: AB_2534014
Goat anti-mouse IgG1 Alexa Fluor^™^ 488 (1:2000)	Invitrogen	Cat#: A21121; RRID: AB_2535764
Goat anti-mouse IgG1 Alexa Fluor^™^ 568 (1:2000)	Invitrogen	Cat#: A21124; RRID: AB_2535766
Goat anti-mouse IgG1 Alexa Fluor^™^ 647 (1:2000)	Invitrogen	Cat#: A21240; RRID: AB_2535809
Goat anti-mouse IgG2a Alexa Fluor^™^ 488 (1:2000)	Invitrogen	Cat#: A21131; RRID: AB_2535771
Goat anti-mouse IgG2a Alexa Fluor^™^ 568 (1:2000)	Invitrogen	Cat#: A21134; RRID: AB_2535773
Goat anti-mouse IgG2a Alexa Fluor^™^ 647 (1:2000)	Invitrogen	Cat#: A21241; RRID: AB_2535810
Goat anti-mouse IgG2b Alexa Fluor^™^ 488 (1:2000)	Invitrogen	Cat#: A21141; RRID: AB_2535778
Goat anti-mouse IgG2b Alexa Fluor^™^ 568 (1:2000)	Invitrogen	Cat#: A21144; RRID: AB_2535780
Goat anti-mouse IgG2b Alexa Fluor^™^ 647 (1:2000)	Invitrogen	Cat#: A21242; RRID: AB_2535811
Donkey anti-rabbit Alexa Fluor^™^ 488 (1:1000)	Abcam	Cat#: ab150061; RRID: AB_2571722
Donkey anti-rabbit Alexa Fluor^™^ 488 (1:1000–2000)	Invitrogen	Cat#: A21206; RRID: AB_2535792
Donkey anti-rabbit Alexa Fluor^™^ 568 (1:1000–2000)	Invitrogen	Cat#: A10042; RRID: AB_2534017
Donkey anti-rabbit Alexa Fluor^™^ 594 (1:1000–2000)	Invitrogen	Cat#: A21207; RRID: AB_141637
Donkey anti-rabbit Alexa Fluor^™^ 647 (1:1000–2000)	Invitrogen	Cat#: A31573; RRID: AB_2536183
Donkey anti-rabbit Alexa Fluor^™^ 680 (1:1000–2000)	Invitrogen	Cat#: A10043; RRID: AB_2534018
Donkey anti-goat Alexa Fluor^™^ 488 (1:1000)	Abcam	Cat#: ab150133; RRID: AB_2832252
Goat anti-chicken Alexa Fluor^™^ 594 (1:1000)	Invitrogen	Cat#: A11042; RRID: AB_2534099

Chemicals, peptides, and recombinant proteins		

Stempro Accutase Cell Dissociation Reagent	Gibco	Cat#: A1110501
Y27632	Stemgent	Cat#: 04-0012-02
Essential 8 Flex medium	Gibco	Cat#: A2858501
Vitronectin	Gibco	Cat#: A14700
Normocin	Invivogen	Cat#: ant-nr-1
EDTA	Gibco	Cat#: 15575020
E6 medium	Gibco	Cat#: A1516401
Matrigel Growth Factor Reduced	Corning	Cat#: 354230
SB431542	Stemgent	Cat#: 04-0010-05
basic FGF	Peprotech	Cat#: 100-18B
BMP-4	Peprotech	Cat#: 120-05
BMP-4	Stemgent	Cat#: 03-0007
BMP-4	R&D	Cat#: 314-BP
BMP-4	R&D	Cat#: 314-BPE
LDN	Stemgent	Cat#: 04-0074-02
CHIR99021	Stemgent	Cat#: 04-0004-02
Advanced DMEM:F12 medium	Gibco	Cat#: 12634010
Neurobasal medium	Gibco	Cat#: 21103049
B27 Supplement without vitamin A	Gibco	Cat#: 12587010
N2 Supplement	Gibco	Cat#: 17502048
Glutamax	Gibco	Cat#: 35050061
β-Mercaptoethanol	Gibco	Cat#: 21985023
Low Melting Point Agarose	Invitrogen	Cat#: 16520050
PBS	Gibco	Cat#: 14190144
TrypLE	Gibco	Cat#: 12605036
Bovine Serum Albumin	Sigma-Aldrich	Cat#: A7030
Trypan Blue	Gibco	Cat#: 15250061
Tris-HCl	Millipore-Sigma	Cat#: T2194
NaCl	Millipore-Sigma	Cat#: 59222C
MgCl2	Millipore-Sigma	Cat#: M1028
Nonidet P40 Substitute	Millipore-Sigma	Cat#: 74385
DEPC-treated water	Invitrogen	Cat#: 750024
Rnase inhibitor	Millipore-Sigma	Cat#: 3335402001
RNAlater	Invitrogen	Cat#: AM7020

Critical commercial assays		

Chromium Next GEM Single Cell 3'	10X Genomics	Cat#: PN-1000128
Library & Gel Bead Kit v3.1		
Chromium Next GEM Single Cell 3'	10X Genomics	Cat#: PN-120267
Library & Gel Bead Kit v2		
Anti-Nuclear Pore Complex Proteins	Merck	Cat#: MAb414
Fc-blocking reagent	Biolegend	Cat#: 422302

Deposited data		

RNA sequencing data IEO	This manuscript	GEO: GSE214099
RNA sequencing data human inner ear	This manuscript	GEO: GSE213796

Experimental models: Cell lines		

WA01 ESC line	WiCell	N/A
AICS-0074 iPSC line (WTC-SOX2)	Allen Institute	N/A
WTC-GCaMP iPSC line	Bruce Conklin at the Gladstone Institutes/UCSF	N/A
LUMC0004iCtrl10 iPSC line (LUMC04i10)	LUMC iPSC Hotel	N/A
LUMC044iCtrl44 iPSC line (LUMC44i44)	LUMC iPSC Hotel	N/A
SAH0047-02 iPSC line	BCH Human Neuron Core	N/A
GON0515-03 iPSC line	BCH Human Neuron Core	N/A
GON0926-02 iPSC line	BCH Human Neuron Core	N/A

Software and algorithms		

CellChat 1.4.0	Jin et al.^74^	https://github.com/sqjin/CellChat
CellRanger 6.1.0	10× Genomics	https://www.10xgenomics.com/support
Rstudio version 1.4.1106	Rstudio	https://www.rstudio.com/
Seurat version 4.1.0	Satija et al.^118^	https://satijalab.org/seurat/
ggplot2 version 3.3.6	Wickham^119^	https://ggplot2.tidyverse.org/
Nebulosa version 1.0.2	Alquicira-Hernandez et al.^120^	www.github.com/powellgenomicslab/Nebulosa
Symphony version 0.1.0	Kang et al.^65^	https://github.com/immunogenomics/symphony
ImageJ-Fiji	ImageJ	https://imagej.net/software/fiji/
R version 4.0.5	R-project	https://www.r-project.org/
SCTransform version 0.3.3	Hafemeister et al.^121^	https://github.com/satijalab/sctransform
LAS X Life Science	Leica	https://www.leica-microsystems.com/
Adobe Illustrator 2022	Adobe	https://www.adobe.com/
Adoble Photoshop 2022	Adobe	https://www.adobe.com/
Leica Application Suite (LAS)	Leica	https://www.leica-microsystems.com/
SerialEM version 4.0	Mastronarde^122^	https://bio3d.colorado.edu/SerialEM/
IMOD version 4.11	Kremer et al.^123^	https://bio3d.colorado.edu/imod/

Other		

96-well U-bottom plates with a super-low cell attachment surface	Thermo Scientific	Cat#: 174925
24-well plates with a super-low cell attachment surface	Thermo Scientific	Cat#: 174930
Dounce tissue grinder	Kimble	Cat#: 885300-0002
70 μm MACS SmartStrainer	Miltenyi Biotec	Cat#: 130-098-462
40 μm Flowmi cell strainer	Bel-Art	Cat#: H13680-0040
10 μm Pluristrainer	PluriSelect	Cat#: 43-10020-40
20 μm Pluristrainer	PluriSelect	Cat#: 43-10010-40
Cryomolds	Andwin Scientific	Cat#: 25608
Ultra 45° diamond knife	Diatome	Cat#: Ultra 45°
Histo diamond knife	Diatome	Cat#: Histo
Single-slot copper grids	Veco	Cat#: 12563-CU
Aminoalkylsilane-coated glass slides	VWR	Cat#: 631-1165

## Highlights

- Targeted hiPSC differentiation efficiency is dependent on initial BMP signaling
- Transcriptomics defines periotic mesenchymal cells, vestibular hair cells, and more
- Genes linked to congenital inner ear dysfunction are expressed in the organoid model
- Cell communication analysis reveals a role of endothelium in sensory development
