# A single-cell level comparison of human inner ear organoids with the human cochlea and vestibular organs

**DOI:** 10.1016/j.celrep.2023.113527

**Published:** 2023-11-29

**Authors:** Wouter H. van der Valk, Edward S.A. van Beelen, Matthew R. Steinhart, Carl Nist-Lund, Daniel Osorio, John C.M.J. de Groot, Liang Sun, Peter Paul G. van Benthem, Karl R. Koehler, Heiko Locher

## Key resources table

- Original: Mouse monoclonal anti-WRHN (1:50)
- Correction: Mouse monoclonal anti-WHRN (1:50)
- Original: Mouse monoclonal anti-TUBB3A (1:100) Biolegend
- Correction: Mouse monoclonal anti-TUBB3 (1:100) Biolegend
- Original: Mouse monoclonal anti-TUBB3A (1:100) R&D Systems
- Correction: Mouse monoclonal anti-TUBB3 (1:100) R&D Systems

## Method details

- Original: 50 μg/mL bFGF
- Correction: 50 ng/mL bFGF

These errors arose from oversight during the proofreading process and were not identified until after publication. We sincerely apologize for any confusion they may have caused. The corrections aim to ensure the accuracy and clarity of the methods described in the STAR Methods.

The authors regret this error.

