# Supplementary material for: A single-cell level comparison of human inner ear organoids with the human cochlea and vestibular organs
Source: Cell Rep. Author manuscript; Available in PMC 2023 Oct 23. (PMC10592453; doi:10.1016/j.celrep.2023.112623)
Supplement: 1 [file NIHMS1912791-supplement-1.pdf]

Cell Reports, Volume *42*

Supplemental information

A single-cell level comparison of human inner ear organoids with the human cochlea and vestibular organs

Wouter H. van der Valk, Edward S.A. van Beelen, Matthew R. Steinhart, Carl Nist-Lund, Daniel Osorio, John C.M.J. de Groot, Liang Sun, Peter Paul G. van Benthem, Karl R. Koehler, and Heiko Locher

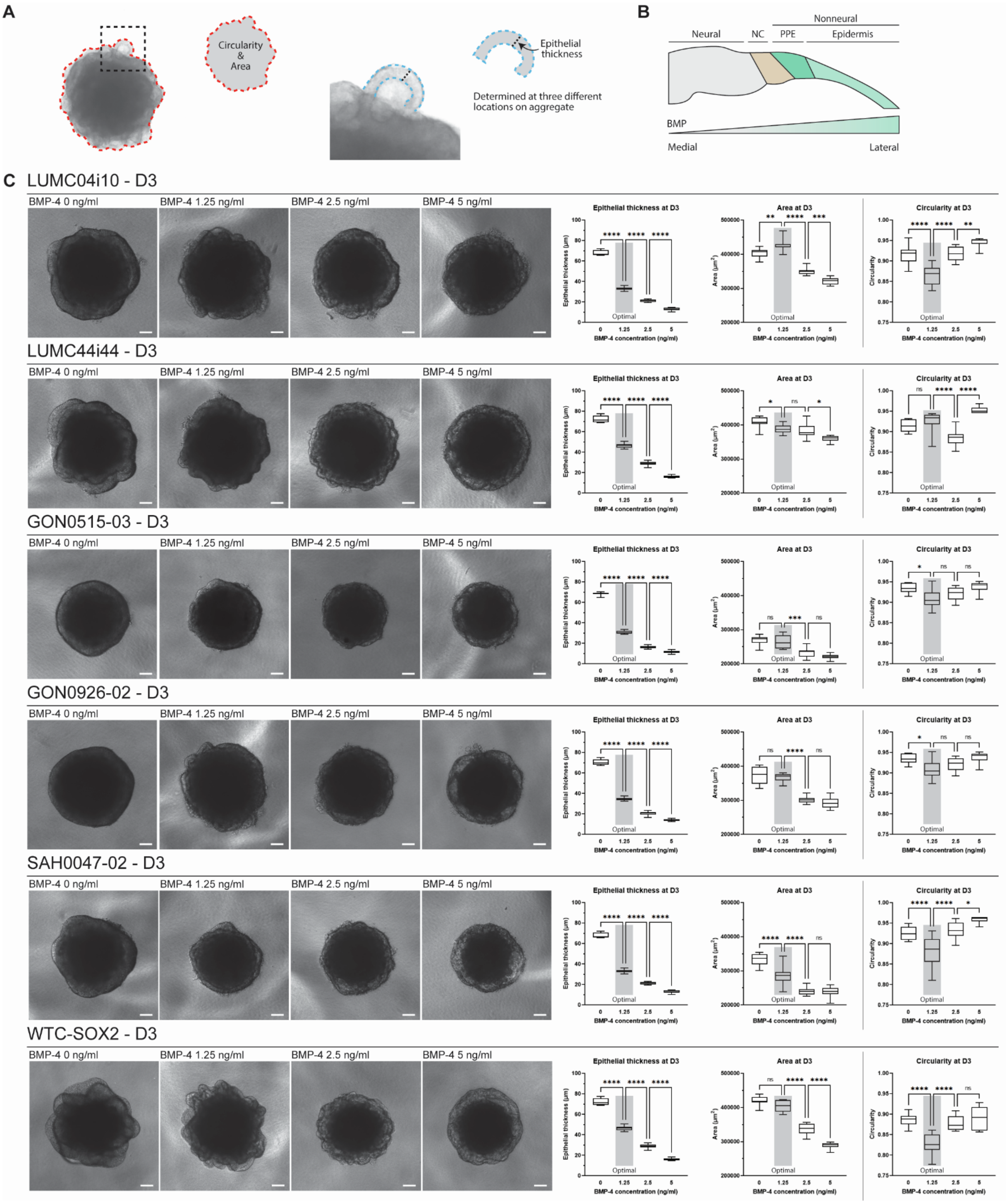

Figure S1. Analyses of differentiation efficiency using morphological characteristics of early IEO-differentiation. **Related to Figure 1. A.** Measurements of D3 aggregates using ImageJ: circularity, area, and epithelial thickness. Epithelial thickness was measured at three different locations in the aggregate. **B.** Illustration showing relative thickness difference during ectodermal specification at the gastrula stage which is achieved by a BMP gradient. **C.** Representative phase-contrast images of D3 aggregates treated with different concentrations of BMP-4 for all hiPSC lines. Results of circularity, area, and epithelial thickness are plotted per cell line. Statistical significance was analyzed with 2-way ANOVA with a Sidak correction for multiple comparisons. Data was considered statistically significant if $p < 0.05$. n=10 per datapoint was graphed in a min-max box plot. A representative graph of at least two individual experiments is shown. The optimal concentration for this set of experiments is highlighted. Scale bars, 100 µm. NC: neural crest; PPE: pre-placodal ectoderm. ns: $p > 0.05$; *: $p \leq 0.05$; **: $p \leq 0.01$; ***: $p \leq 0.001$; ****: $p \leq 0.0001$.

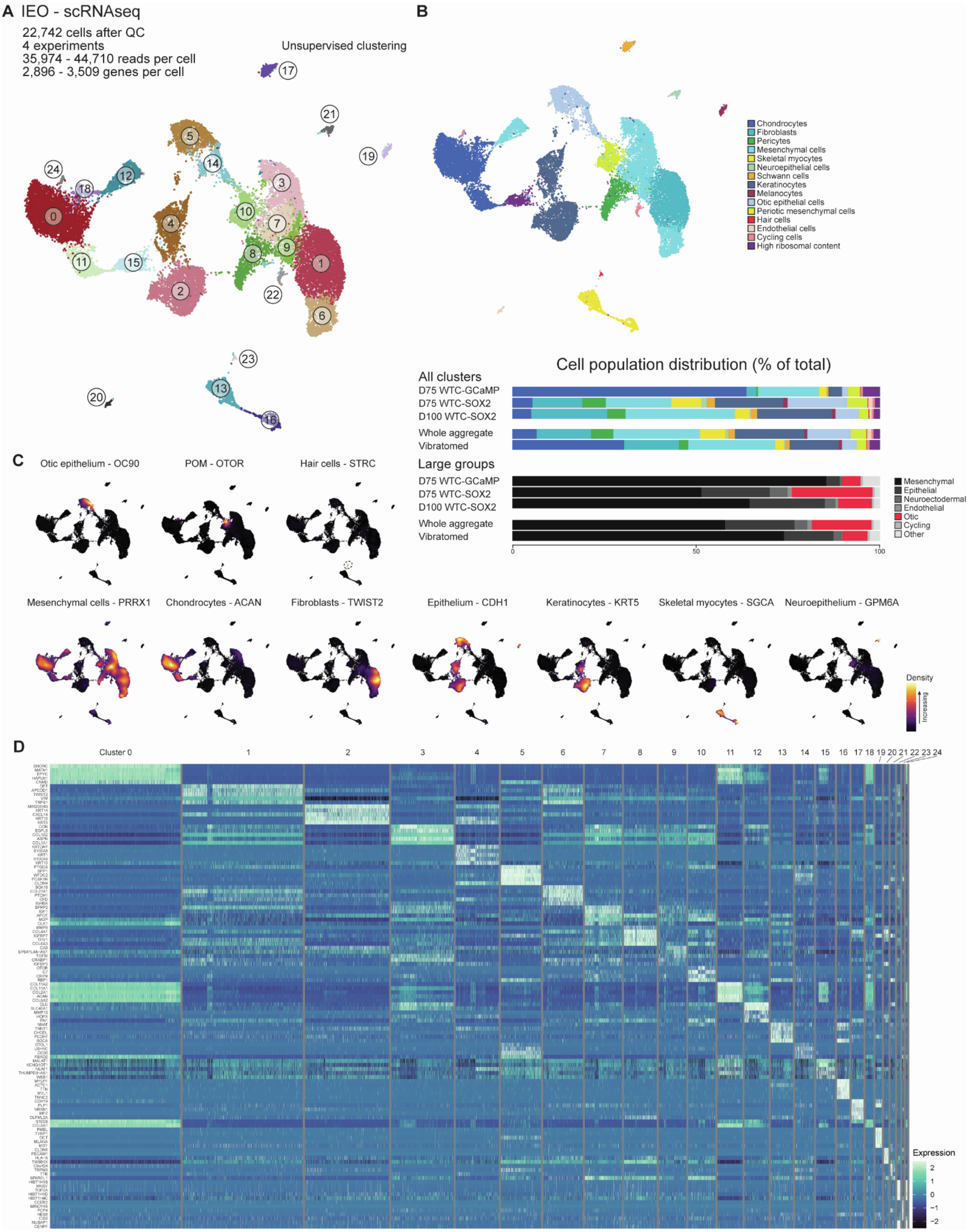

Figure S2. scRNAseq unravels the cell type diversity of D75-D100 IEOs. Related to Figure 2. A. Overview UMAP plot of D75 (WTC-GCaMP and WTC-SOX2) and D100 (WTC-SOX2) dataset of 22,742 cells. 2-4 pooled aggregates per cell line at D75 and/or D100, n=1-2 experimental replicates. **B.** Cell type annotation with relative cell population contribution of cell types and large groups. **C.** Marker genes involved in cell type annotation assignment. **D.** Heatmap showing expression patterns of the top 5 differentially expressed genes per unsupervised cluster.

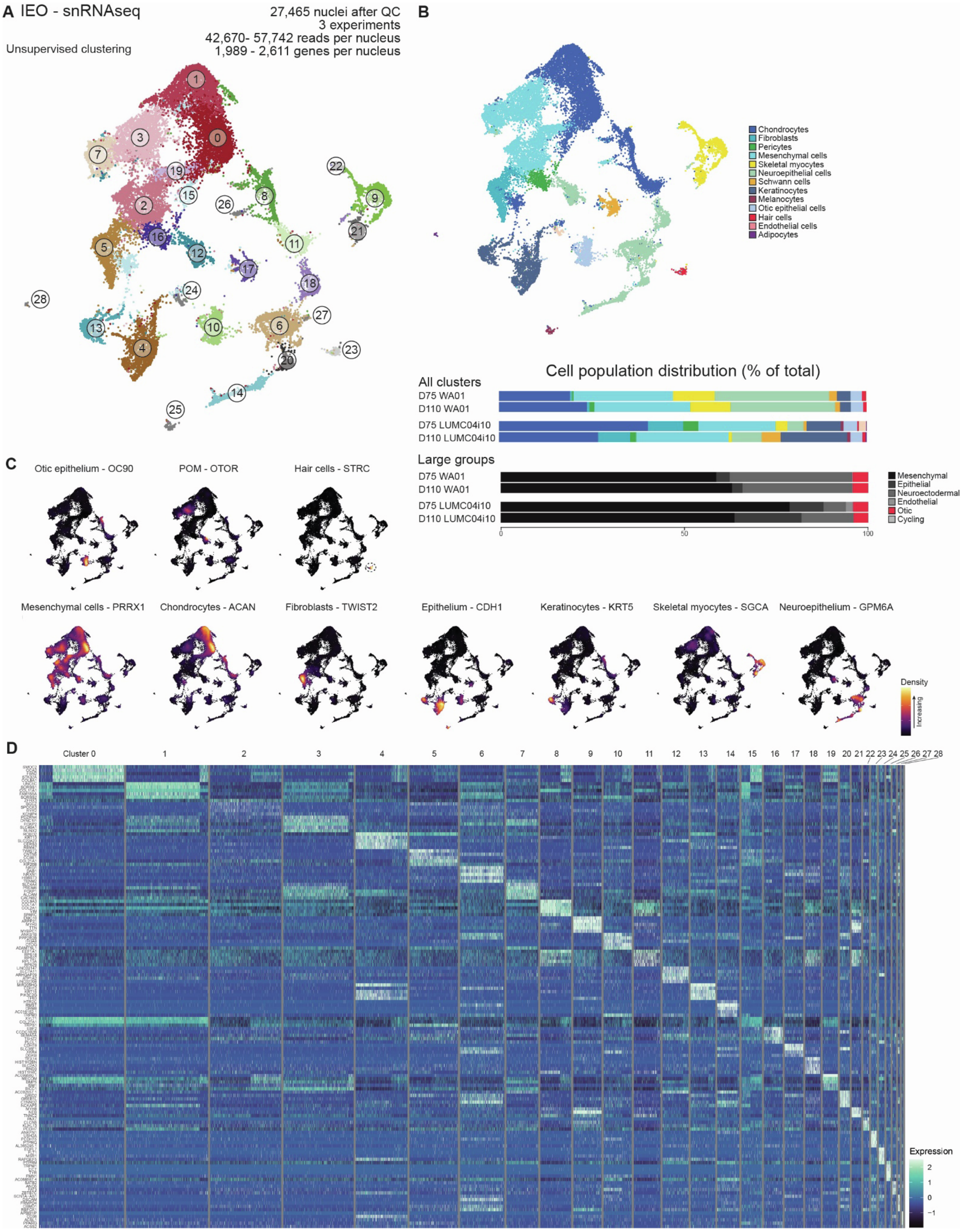

Figure S3. snRNAseq of D75-D110 aggregates shows a similar cell type diversity. Related to Figure 2. A. Overview UMAP plot of the D75 and D110 (WA01, LUMC04i10) dataset of 27,465 nuclei. 2-4 pooled aggregates per cell line at D75 and/or D110, n=1-2 experimental replicates. **B.** Cell type annotation with relative cell population contribution of cell types and large groups. **C.** Marker genes involved in cell type annotation assignment. **D.** Heatmap showing expression patterns of the top 5 differentially expressed genes per unsupervised cluster.

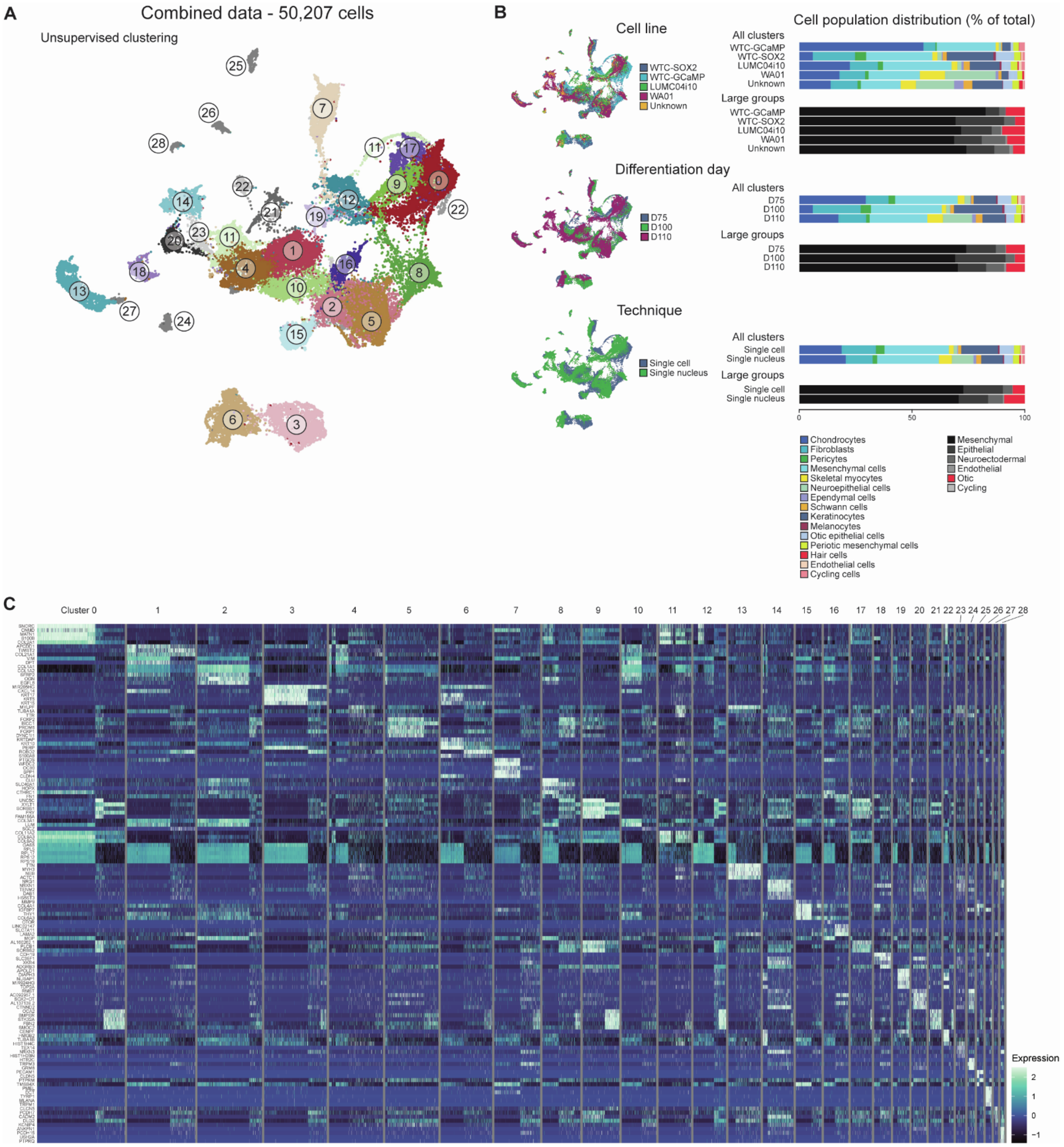

Figure S4. Integration of the scRNAseq and snRNAseq IEO datasets. Related to Figure 2. A. Overview UMAP plot of the integrated combined dataset composed of 50,207 cells and nuclei of D75 (WTC-GCaMP, WTC-SOX2, WA01, LUMC04i10), D100 (WTC-SOX2) and D110 (WA01 and LUMC04i10) aggregates. **B.** UMAP plots and relative cell population contribution showing overlap between the cell lines, differentiation days and techniques used. Cell line “Unknown” means that using SNP demultiplexing, no distinction could be made between LUMC04i10 or WA01. **D.** Heatmap showing expression patterns of the top 5 differentially expressed genes per unsupervised cluster.

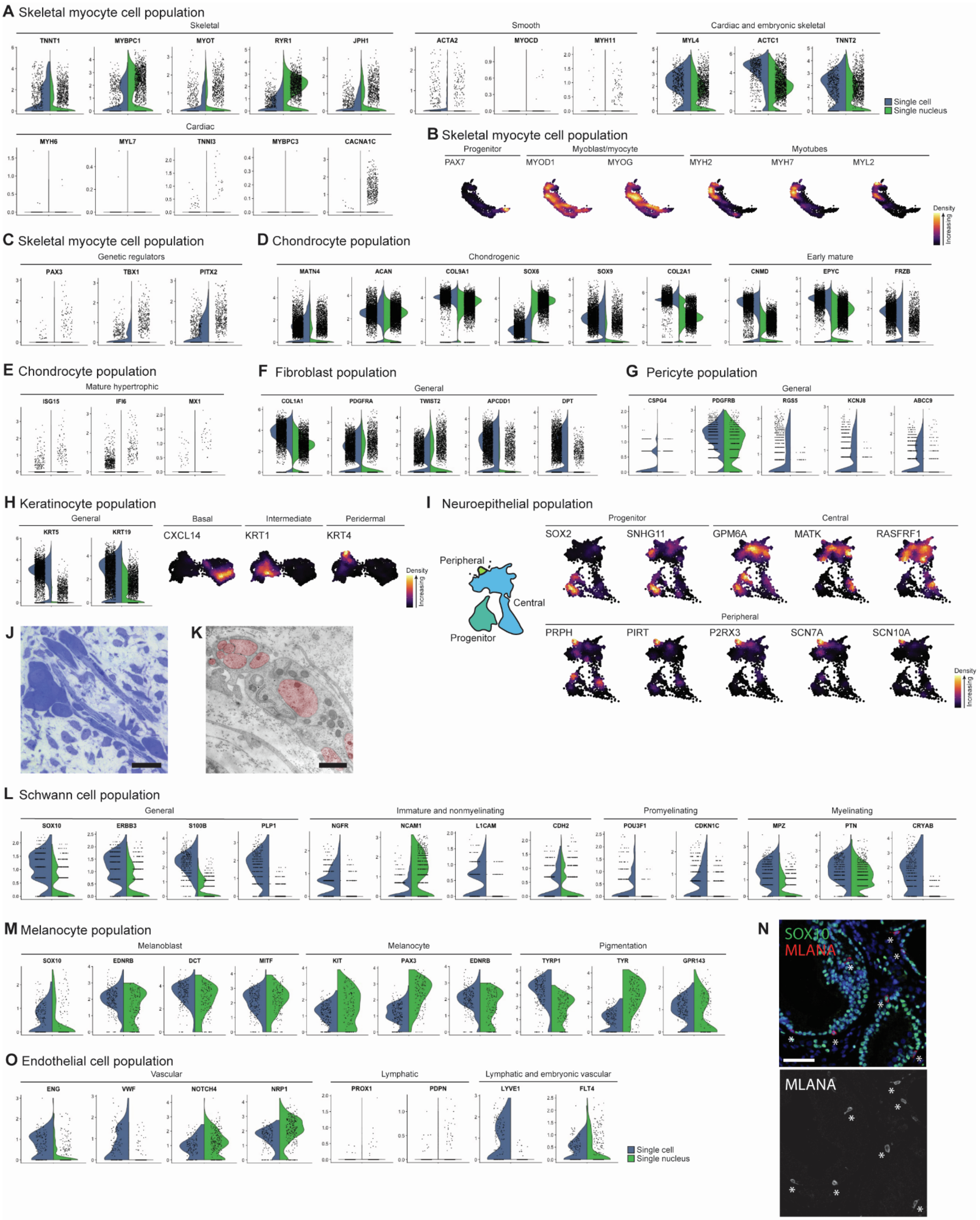

Figure S5. Cell type and development-specific marker gene expression of cell types in D75-D110 aggregates. Related to Figure 2. A. Marker gene expression within the skeletal myocyte population of skeletal myocytes, smooth muscle myocytes, cardiomyocytes, and of both cardiomyocytes and fetal skeletal myocytes. **B.** Density plots of the skeletal myocyte cell cluster, showing expression of progenitor, myoblast and myotube markers. **C.** Gene expression within the skeletal myocyte cell population of genetic regulators of caudal (*PAX3*) and cranial skeletal myocytes (*TBX1*, *PITX2*). **D.** Marker gene expression within the chondrocyte population consisting of chondrogenic and early mature chondrocytes. **E.** Marker gene expression within the chondrocyte population of mature hypertrophic markers. **F.** Marker gene expression within the fibroblast population. **G.** Marker gene expression within the pericyte population. **H.** Marker gene expression within the keratinocyte population and density

plots showing expression of basal, intermediate, and peridermal markers. **I.** Schematic dividing the neuroepithelial cluster into progenitor, central, and peripheral neuroepithelial cells based on density plots of the neuroepithelial cell cluster, showing expression of progenitor, central, and peripheral identities. **J.** Staining (methylene blue-azure II) depicting a group of neurons surrounded by glial (Schwann) cells lying freely in the mesenchymal stroma in close proximity to an IEO-vesicle within a D74 aggregate. Representative image of n≥3. Scale bar, 25 µm. **K.** TEM image showing neurons (in red) surrounded by a Schwann cell situated underneath IEO-vesicle within a D74 aggregate. Representative image of n=3. Scale bar, 1 µm. **L.** Marker gene expression within the Schwann cell population of immature, nonmyelinating, promyelinating, and myelinating markers. **M.** Marker gene expression within the melanocyte population containing melanoblast, melanocyte, and pigmented stages. **N.** $MLANA^+$ melanocytes in proximity of $SOX10^+$ IEO-vesicles in a D75 aggregate. Asterisks show location of melanocytes. Representative images of n≥6 aggregates of at least 2 individual experiments. Scale bar, 50 µm. **O.** Marker gene expression within the endothelial cell population of vascular, lymphatic, and combined lymphatic & fetal vascular genes.

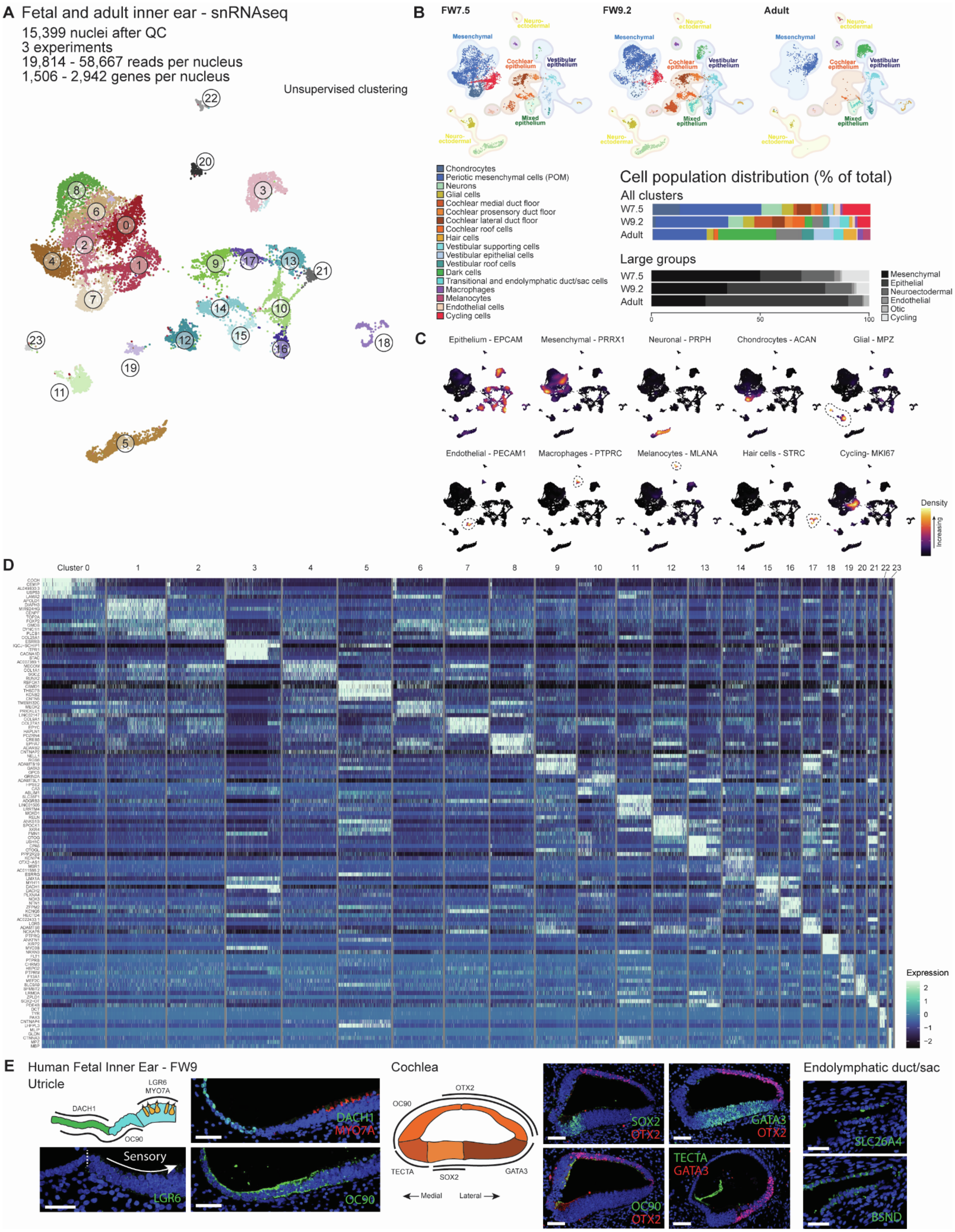

Figure S6. snRNAseq of fetal and adult human inner ear tissue. **Related to Figure 3. A.** Overview UMAP plot of the fetal stages (FW7.5, FW9.2) and adult inner ear tissue as a combined dataset of 15,399 cells. N=1 per developmental timepoint. **B.** Cell type annotated UMAP plots and relative cell population contribution per developmental timepoint. **C.** Marker genes involved in cell type annotation assignment. **D.** Heatmap showing expression patterns of the top 5 differentially expressed genes per unsupervised cluster. **E.** Validation of differentially expressed genes and marker genes used for cell type annotation. Representative images of n≥2 with matching fetal age. Scale bar, 50 µm.

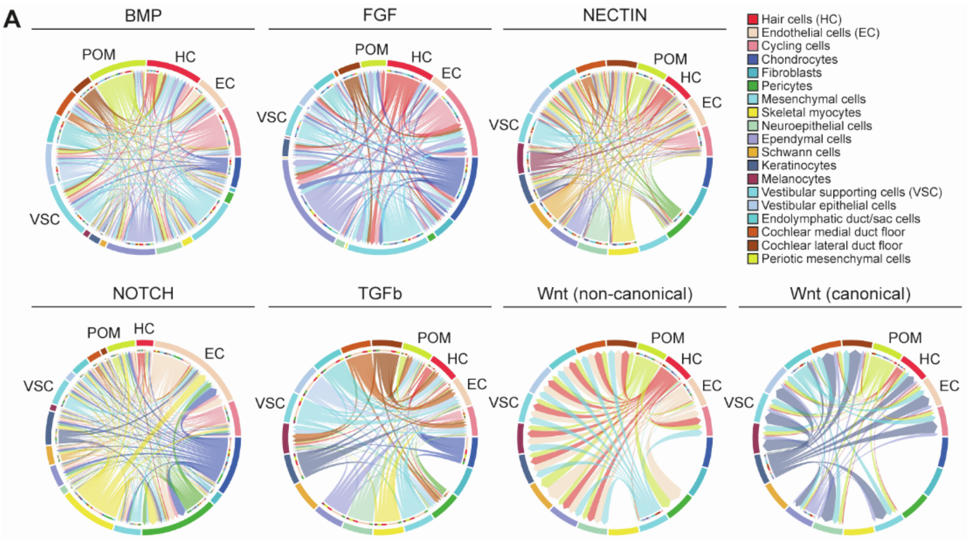

B

Signalling pathways			
Endothelial cells to hair cells		Hair cells to endothelial cells	
ACTIVIN	MK	ANGPT	MPZ
AGRN	MPZ	ANGPTL	NCAM
ANGPT	NCAM	BMP	NECTIN
ANGPTL	NECTIN	CADM	NEGR
BMP	NEGR	CALCR	NGL
CADM	NOTCH	CD46	NOTCH
CALCR	NRG	CD99	NRG
CD39	PDGF	CDH	NRXN
CD46	PROS	CDH1	NT
CD99	PTPRM	COLLAGEN	PDGF
CDH	SEMA3	CXCL	PERIOSTIN
COLLAGEN	SEMA4	EPHA	PTPRM
EGF	SEMA5	EPHB	SEMA3
EPHA	SEMA6	FGF	SEMA4
EPHB	SEMA7	FN1	SEMA5
FN1	TGFβ	GDF	TENASCIN
GDF	THBS	HSPG	TGFβ
GRN	VISFATIN	JAM	THBS
HSPG	VISTA	LAMININ	VEGF
JAM	WNT	MIF	VISFATIN
LAMININ		MK	Wnt

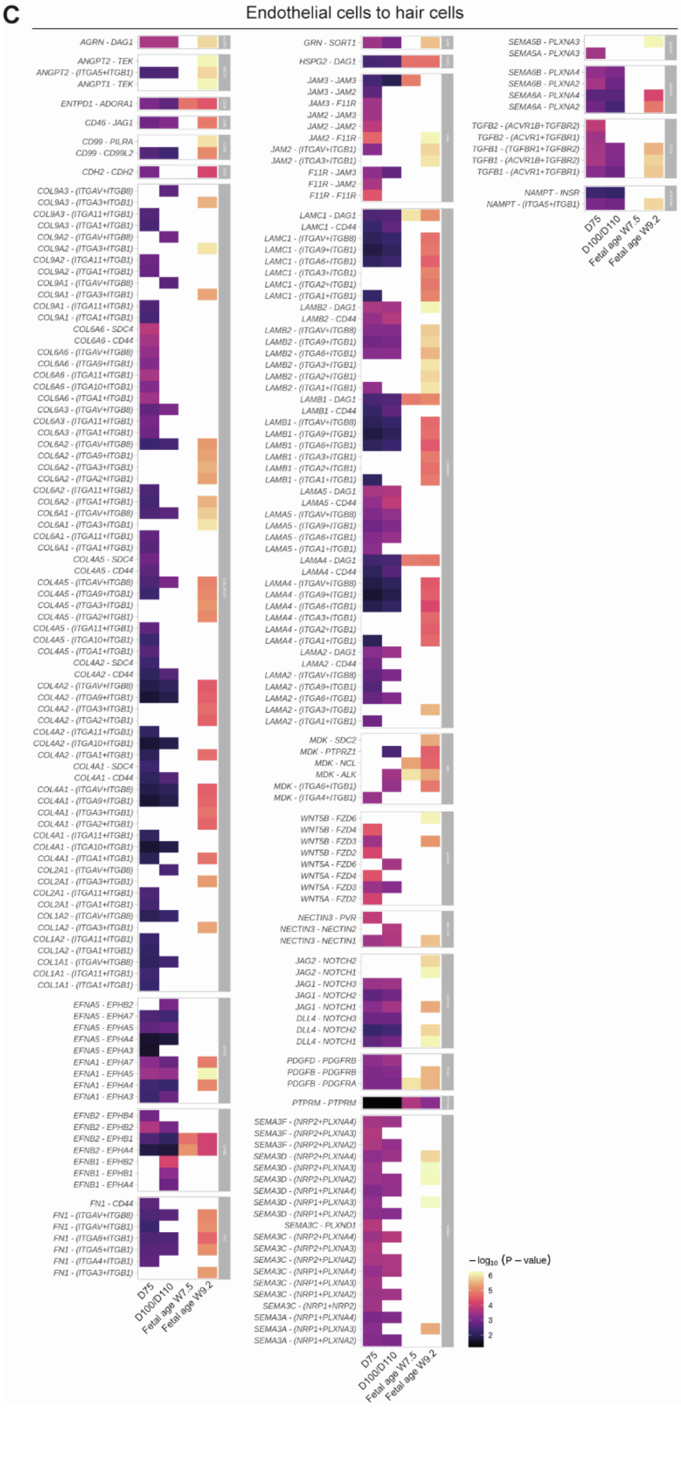

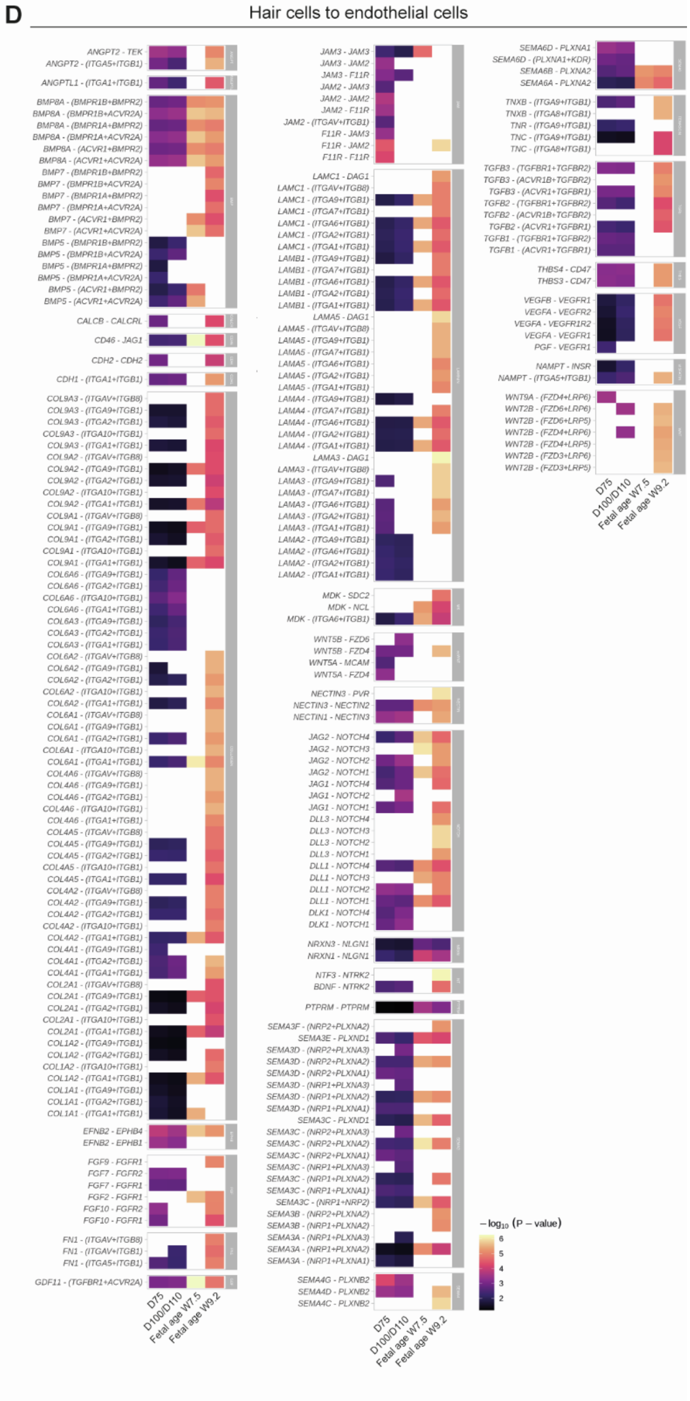

Figure S7. Cell-cell communication analyses. Related to Figure 7. A. Chord diagrams showing the interactions between cell types within a specific signaling pathway. Arrows point from the sender towards the receiver cell types. **B.** Table showing the signaling pathways that were computed to interact between endothelial cells and hair cells. **C.** Ligand-receptor interactions from endothelial cells to hair cells within the IEO (D75 and D100-110 showed in separate columns) that could be validated in the fetal data from the human inner ear atlas (FW7.5 and FW9.2 showed in separate columns). **D.** Ligand-receptor interactions from hair cells to endothelial cells within the IEO (D75 and D100-110 showed in separate columns) that could be validated in the fetal data from the human inner ear atlas (FW7.5 and FW9.2 showed in separate columns). EC: endothelial cells; HC: hair cells; POM: periotic mesenchyme; VSC: vestibular supporting cells.

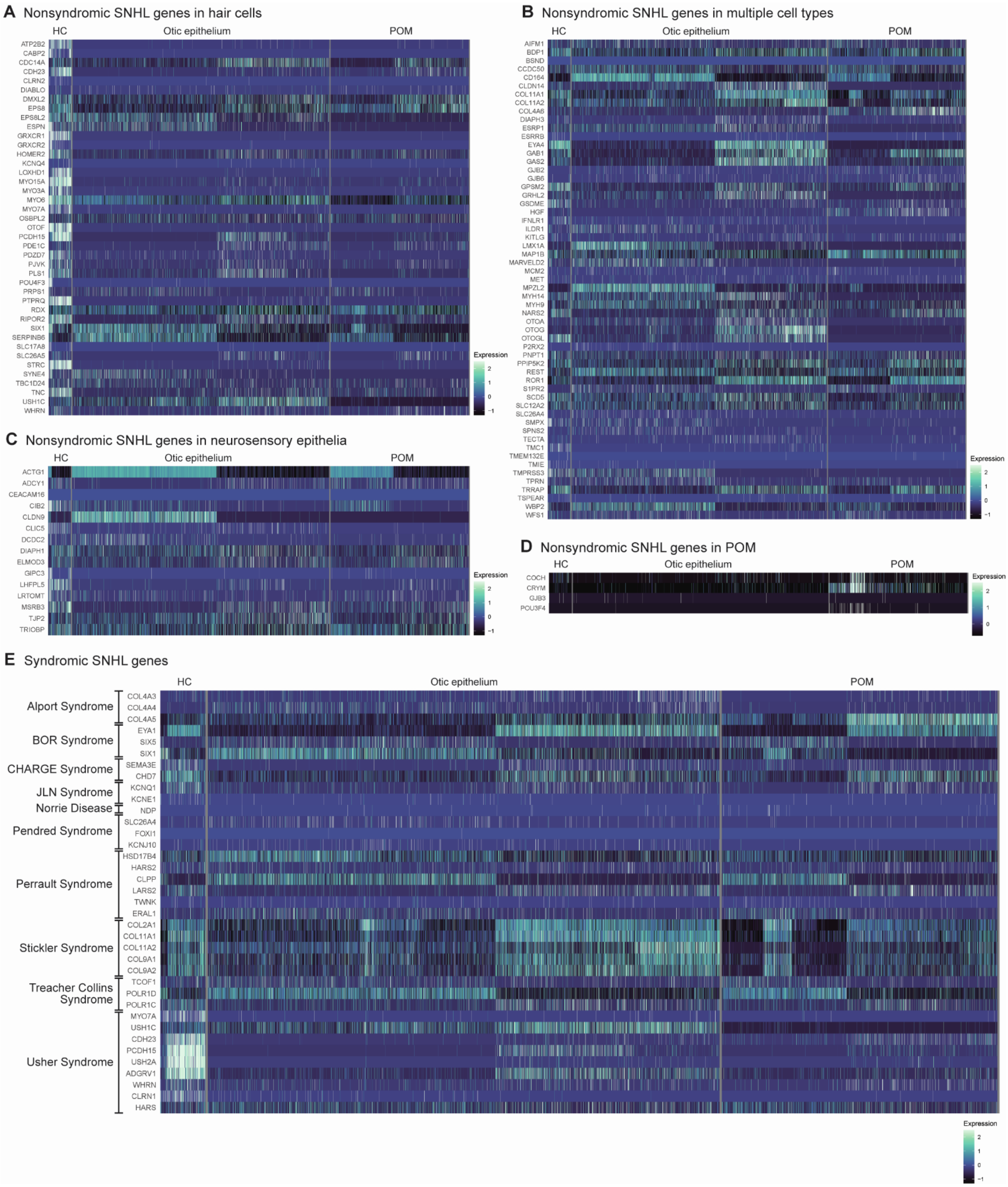

Figure S8. Gene expression of SNHL genes in the otic populations of the D75-D110 IEOs. Related to Figure 2 and Table S2. A. Heatmap showing expression patterns of nonsyndromic SNHL genes expressed in hair cells. **B.** Heatmap showing expression of nonsyndromic SNHL genes expressed in multiple inner ear cell types. **C.** Heatmap showing expression of nonsyndromic SNHL genes expressed in neurosensory epithelia. **D.** Heatmap showing expression of nonsyndromic SNHL genes expressed in POM. **E.** Heatmap showing expression of a selection of syndromic SNHL genes. BOR: Branchio-Oto-Renal syndrome; HC: hair cell; JLN: Jervell & Lange-Nielsen syndrome; POM: periotic mesenchyme.

References – Table S2

[S1] Zhu, M., Yang, T., Wei, S., DeWan, A.T., Morell, R.J., Elfenbein, J.L., Fisher, R.A., Leal, S.M., Smith, R.J., and Friderici, K.H. (2003). Mutations in the gamma-actin gene (ACTG1) are associated with dominant progressive deafness (DFNA20/26). Am J Hum Genet 73, 1082-1091. 10.1086/379286.

[S2] van Wijk, E., Krieger, E., Kemperman, M.H., De Leenheer, E.M., Huygen, P.L., Cremers, C.W., Cremers, F.P., and Kremer, H. (2003). A mutation in the gamma actin 1 (ACTG1) gene causes autosomal dominant hearing loss (DFNA20/26). J Med Genet 40, 879-884. 10.1136/jmg.40.12.879.

[S3] Perrin, B.J., Sonnemann, K.J., and Ervasti, J.M. (2010). β-actin and γ-actin are each dispensable for auditory hair cell development but required for Stereocilia maintenance. PLoS Genet 6, e1001158. 10.1371/journal.pgen.1001158.

[S4] Santos-Cortez, R.L., Lee, K., Giese, A.P., Ansar, M., Amin-Ud-Din, M., Rehn, K., Wang, X., Aziz, A., Chiu, I., Hussain Ali, R., et al. (2014). Adenylate cyclase 1 (ADCY1) mutations cause recessive hearing impairment in humans and defects in hair cell function and hearing in zebrafish. Hum Mol Genet 23, 3289-3298. 10.1093/hmg/ddu042.

[S5] Weston, M.D., Luijendijk, M.W., Humphrey, K.D., Möller, C., and Kimberling, W.J. (2004). Mutations in the VLGR1 gene implicate G-protein signaling in the pathogenesis of Usher syndrome type II. Am J Hum Genet 74, 357-366. 10.1086/381685.

[S6] Zong, L., Guan, J., Ealy, M., Zhang, Q., Wang, D., Wang, H., Zhao, Y., Shen, Z., Campbell, C.A., Wang, F., et al. (2015). Mutations in apoptosis-inducing factor cause X-linked recessive auditory neuropathy spectrum disorder. J Med Genet 52, 523-531. 10.1136/jmedgenet-2014-102961.

[S7] Wang, H., Bing, D., Li, J., Xie, L., Xiong, F., Lan, L., Wang, D., Guan, J., and Wang, Q. (2020). High Frequency of AIFM1 Variants and Phenotype Progression of Auditory Neuropathy in a Chinese Population. Neural Plast 2020, 5625768. 10.1155/2020/5625768.

[S8] Smits, J.J., Oostrik, J., Beynon, A.J., Kant, S.G., de Koning Gans, P.A.M., Rotteveel, L.J.C., Klein Wassink-Ruiter, J.S., Free, R.H., Maas, S.M., van de Kamp, J., et al. (2019). De novo and inherited loss-of-function variants of ATP2B2 are associated with rapidly progressive hearing impairment. Hum Genet 138, 61-72. 10.1007/s00439-018-1965-1.

[S9] Hill, J.K., Williams, D.E., LeMasurier, M., Dumont, R.A., Strehler, E.E., and Gillespie, P.G. (2006). Splice-site A choice targets plasma-membrane Ca2+-ATPase isoform 2 to hair bundles. J Neurosci 26, 6172-6180. 10.1523/jneurosci.0447-06.2006.

[S10] Girotto, G., Abdulhadi, K., Buniello, A., Vozzi, D., Licastro, D., d'Eustacchio, A., Vuckovic, D., Alkowari, M.K., Steel, K.P., Badii, R., and Gasparini, P. (2013). Linkage study and exome sequencing identify a BDP1 mutation associated with hereditary hearing loss. PLoS One 8, e80323. 10.1371/journal.pone.0080323.

[S11] Riazuddin, S., Anwar, S., Fischer, M., Ahmed, Z.M., Khan, S.Y., Janssen, A.G., Zafar, A.U., Scholl, U., Husnain, T., Belyantseva, I.A., et al. (2009). Molecular basis of DFNB73: mutations of BSND can cause nonsyndromic deafness or Bartter syndrome. Am J Hum Genet 85, 273-280. 10.1016/j.ajhg.2009.07.003.

[S12] Rickheit, G., Maier, H., Strenzke, N., Andreescu, C.E., De Zeeuw, C.I., Muenscher, A., Zdebik, A.A., and Jentsch, T.J. (2008). Endocochlear potential depends on Cl- channels: mechanism underlying deafness in Bartter syndrome IV. Embo j 27, 2907-2917. 10.1038/emboj.2008.203.

[S13] Schrauwen, I., Helfmann, S., Inagaki, A., Predoehl, F., Tabatabaiefar, M.A., Picher, M.M., Sommen, M., Zazo Seco, C., Oostrik, J., Kremer, H., et al. (2012). A mutation in CABP2, expressed in cochlear hair cells, causes autosomal-recessive hearing impairment. Am J Hum Genet 91, 636-645. 10.1016/j.ajhg.2012.08.018.

[S14] Oestreicher, D., Picher, M.M., Rankovic, V., Moser, T., and Pangrsic, T. (2021). Cabp2-Gene Therapy Restores Inner Hair Cell Calcium Currents and Improves Hearing in a DFNB93 Mouse Model. Front Mol Neurosci 14, 689415. 10.3389/fnmol.2021.689415.

[S15] Modamio-Hoybjor, S., Mencia, A., Goodyear, R., del Castillo, I., Richardson, G., Moreno, F., and Moreno-Pelayo, M.A. (2007). A mutation in CCDC50, a gene encoding an effector of epidermal growth factor-mediated cell signaling, causes progressive hearing loss. Am J Hum Genet 80, 1076-1089. 10.1086/518311.

[S16] Nyegaard, M., Rendtorff, N.D., Nielsen, M.S., Corydon, T.J., Demontis, D., Starnawska, A., Hedemand, A., Buniello, A., Niola, F., Overgaard, M.T., et al. (2015). A Novel Locus Harbouring a Functional CD164 Nonsense Mutation Identified in a Large Danish Family with Nonsyndromic Hearing Impairment. PLoS Genet 11, e1005386. 10.1371/journal.pgen.1005386.

[S17] Delmaghani, S., and El-Amraoui, A. (2020). Inner ear gene therapies take off: Current promises and future challenges. J Clin Med 9. 10.3390/jcm9072309.

[S18] Imtiaz, A., Belyantseva, I.A., Beirl, A.J., Fenollar-Ferrer, C., Bashir, R., Bukhari, I., Bouzid, A., Shaukat, U., Azaiez, H., Booth, K.T., et al. (2018). CDC14A phosphatase is essential for hearing and male fertility in mouse and human. Hum Mol Genet 27, 780-798. 10.1093/hmg/ddx440.

[S19] Bork, J.M., Peters, L.M., Riazuddin, S., Bernstein, S.L., Ahmed, Z.M., Ness, S.L., Polomeno, R., Ramesh, A., Schloss, M., Srisailpathy, C.R., et al. (2001). Usher syndrome 1D and nonsyndromic autosomal recessive deafness DFNB12 are caused by allelic mutations of the novel cadherin-like gene CDH23. Am J Hum Genet 68, 26-37. 10.1086/316954.

[S20] Bolz, H., von Brederlow, B., Ramírez, A., Bryda, E.C., Kutsche, K., Nothwang, H.G., Seeliger, M., del, C.S.C.M., Vila, M.C., Molina, O.P., et al. (2001). Mutation of CDH23, encoding a new member of the cadherin gene family, causes Usher syndrome type 1D. Nat Genet 27, 108-112. 10.1038/83667.

[S21] Wafa, T.T., Faridi, R., King, K.A., Zalewski, C., Yousaf, R., Schultz, J.M., Morell, R.J., Muskett, J., Turriff, A., Tsilou, E., et al. (2021). Vestibular phenotype-genotype correlation in a cohort of 90 patients with Usher syndrome. Clinical Genetics 99, 226-235. https://doi.org/10.1111/cge.13868.

[S22] Siemens, J., Lillo, C., Dumont, R.A., Reynolds, A., Williams, D.S., Gillespie, P.G., and Müller, U. (2004). Cadherin 23 is a component of the tip link in hair-cell stereocilia. Nature 428, 950-955. 10.1038/nature02483.

[S23] Zheng, J., Miller, K.K., Yang, T., Hildebrand, M.S., Shearer, A.E., DeLuca, A.P., Scheetz, T.E., Drummond, J., Scherer, S.E., Legan, P.K., et al. (2011). Carcinoembryonic antigen-related cell adhesion molecule 16 interacts with alpha-tectorin and is mutated in autosomal dominant hearing loss (DFNA4). Proc Natl Acad Sci U S A 108, 4218-4223. 10.1073/pnas.1005842108.
[S24] Booth, K.T., Kahrizi, K., Najmabadi, H., Azaiez, H., and Smith, R.J. (2018). Old gene, new phenotype: splice-altering variants in CEACAM16 cause recessive non-syndromic hearing impairment. J Med Genet 55, 555-560. 10.1136/jmedgenet-2018-105349.
[S25] Vissers, L.E., van Ravenswaaij, C.M., Admiraal, R., Hurst, J.A., de Vries, B.B., Janssen, I.M., van der Vliet, W.A., Huys, E.H., de Jong, P.J., Hamel, B.C., et al. (2004). Mutations in a new member of the chromodomain gene family cause CHARGE syndrome. Nat Genet 36, 955-957. 10.1038/ng1407.
[S26] Abadie, V., Wiener-Vacher, S., Morisseau-Durand, M.P., Porée, C., Amiel, J., Amanou, L., Peigné, C., Lyonnet, S., and Manac'h, Y. (2000). Vestibular anomalies in CHARGE syndrome: investigations on and consequences for postural development. Eur J Pediatr 159, 569-574. 10.1007/s004319900409.
[S27] Ahmed, M., Moon, R., Prajapati, R.S., James, E., Basson, M.A., and Streit, A. (2021). The chromatin remodelling factor Chd7 protects auditory neurons and sensory hair cells from stress-induced degeneration. Commun Biol 4, 1260. 10.1038/s42003-021-02788-6.
[S28] Riazuddin, S., Belyantseva, I.A., Giese, A.P., Lee, K., Indzhykulian, A.A., Nandamuri, S.P., Yousaf, R., Sinha, G.P., Lee, S., Terrell, D., et al. (2012). Alterations of the CIB2 calcium- and integrin-binding protein cause Usher syndrome type 1J and nonsyndromic deafness DFNB48. Nat Genet 44, 1265-1271. 10.1038/ng.2426.
[S29] Michel, V., Booth, K.T., Patni, P., Cortese, M., Azaiez, H., Bahloul, A., Kahrizi, K., Labbé, M., Emptoz, A., Lelli, A., et al. (2017). CIB2, defective in isolated deafness, is key for auditory hair cell mechanotransduction and survival. EMBO Mol Med 9, 1711-1731. 10.15252/emmm.201708087.
[S30] Wilcox, E.R., Burton, Q.L., Naz, S., Riazuddin, S., Smith, T.N., Ploplis, B., Belyantseva, I., Ben-Yosef, T., Liburd, N.A., Morell, R.J., et al. (2001). Mutations in the gene encoding tight junction claudin-14 cause autosomal recessive deafness DFNB29. Cell 104, 165-172. 10.1016/s0092-8674(01)00200-8.
[S31] Sineni, C.J., Yildirim-Baylan, M., Guo, S., Camarena, V., Wang, G., Tokgoz-Yilmaz, S., Duman, D., Bademci, G., and Tekin, M. (2019). A truncating CLDN9 variant is associated with autosomal recessive nonsyndromic hearing loss. Hum Genet 138, 1071-1075. 10.1007/s00439-019-02037-1.
[S32] Seco, C.Z., Oonk, A.M., Domínguez-Ruiz, M., Draaisma, J.M., Gandía, M., Oostrik, J., Neveling, K., Kunst, H.P., Hoefsloot, L.H., del Castillo, I., et al. (2015). Progressive hearing loss and vestibular dysfunction caused by a homozygous nonsense mutation in CLIC5. Eur J Hum Genet 23, 189-194. 10.1038/ejhg.2014.83.
[S33] Gagnon, L.H., Longo-Guess, C.M., Berryman, M., Shin, J.B., Saylor, K.W., Yu, H., Gillespie, P.G., and Johnson, K.R. (2006). The chloride intracellular channel protein CLIC5 is expressed at high levels in hair cell stereocilia and is essential for normal inner ear function. J Neurosci 26, 10188-10198. 10.1523/jneurosci.2166-06.2006.
[S34] Jenkinson, E.M., Rehman, A.U., Walsh, T., Clayton-Smith, J., Lee, K., Morell, R.J., Drummond, M.C., Khan, S.N., Naeem, M.A., Rauf, B., et al. (2013). Perrault syndrome is caused by recessive mutations in CLPP, encoding a mitochondrial ATP-dependent chambered protease. Am J Hum Genet 92, 605-613. 10.1016/j.ajhg.2013.02.013.
[S35] Forli, F., Bruschini, L., Franciosi, B., Battini, R., Marinella, G., Berrettini, S., and Lazzerini, F. (2021). A Rare Case of Perrault Syndrome with Auditory Neuropathy Spectrum Disorder: Cochlear Implantation Treatment and Literature Review. Audiol Res 11, 609-617. 10.3390/audiolres11040055.
[S36] Joensuu, T., Hämäläinen, R., Yuan, B., Johnson, C., Tegelberg, S., Gasparini, P., Zelante, L., Pirvola, U., Pakarinen, L., Lehesjoki, A.E., et al. (2001). Mutations in a novel gene with transmembrane domains underlie Usher syndrome type 3. Am J Hum Genet 69, 673-684. 10.1086/323610.
[S37] Zallocchi, M., Sisson, J.H., and Cosgrove, D. (2010). Biochemical characterization of native Usher protein complexes from a vesicular subfraction of tracheal epithelial cells. Biochemistry 49, 1236-1247. 10.1021/bi9020617.
[S38] Vona, B., Mazaheri, N., Lin, S.J., Dunbar, L.A., Maroofian, R., Azaiez, H., Booth, K.T., Vitry, S., Rad, A., Rüschendorf, F., et al. (2021). A biallelic variant in CLRN2 causes non-syndromic hearing loss in humans. Hum Genet 140, 915-931. 10.1007/s00439-020-02254-z.
[S39] Dunbar, L.A., Patni, P., Aguilar, C., Mburu, P., Corns, L., Wells, H.R., Delmaghani, S., Parker, A., Johnson, S., Williams, D., et al. (2019). Clarin-2 is essential for hearing by maintaining stereocilia integrity and function. EMBO Mol Med 11, e10288. 10.15252/emmm.201910288.
[S40] Robertson, N.G., Lu, L., Heller, S., Merchant, S.N., Eavey, R.D., McKenna, M., Nadol, J.B., Jr., Miyamoto, R.T., Linthicum, F.H., Jr., Lubianca Neto, J.F., et al. (1998). Mutations in a novel cochlear gene cause DFNA9, a human nonsyndromic deafness with vestibular dysfunction. Nat Genet 20, 299-303. 10.1038/3118.
[S41] Kim, B.J., Kim, A.R., Han, K.H., Rah, Y.C., Hyun, J., Ra, B.S., Koo, J.W., and Choi, B.Y. (2016). Distinct vestibular phenotypes in DFNA9 families with COCH variants. Eur Arch Otorhinolaryngol 273, 2993-3002. 10.1007/s00405-015-3885-1.
[S42] Richards, A.J., Yates, J.R., Williams, R., Payne, S.J., Pope, F.M., Scott, J.D., and Snead, M.P. (1996). A family with Stickler syndrome type 2 has a mutation in the COL11A1 gene resulting in the substitution of glycine 97 by valine in alpha 1 (XI) collagen. Hum Mol Genet 5, 1339-1343. 10.1093/hmg/5.9.1339.
[S43] Acke, F.R., Dhooge, I.J., Malfait, F., and De Leenheer, E.M. (2012). Hearing impairment in Stickler syndrome: a systematic review. Orphanet J Rare Dis 7, 84. 10.1186/1750-1172-7-84.
[S44] Shpargel, K.B., Makishima, T., and Griffith, A.J. (2004). Col11a1 and Col11a2 mRNA expression in the developing mouse cochlea: implications for the correlation of hearing loss phenotype with mutant type XI collagen genotype. Acta Otolaryngol 124, 242-248. 10.1080/00016480410016162.
[S45] McGuirt, W.T., Prasad, S.D., Griffith, A.J., Kunst, H.P., Green, G.E., Shpargel, K.B., Runge, C., Huybrechts, C., Mueller, R.F., Lynch, E., et al. (1999). Mutations in COL11A2 cause non-syndromic hearing loss (DFNA13). Nat Genet 23, 413-419. 10.1038/70516.

[S46] Chen, W., Kahrizi, K., Meyer, N.C., Riazalhosseini, Y., Van Camp, G., Najmabadi, H., and Smith, R.J. (2005). Mutation of COL11A2 causes autosomal recessive non-syndromic hearing loss at the DFNB53 locus. J Med Genet 42, e61. 10.1136/jmg.2005.032615.
[S47] Vikkula, M., Mariman, E.C., Lui, V.C., Zhidkova, N.I., Tiller, G.E., Goldring, M.B., van Beersum, S.E., de Waal Malefijt, M.C., van den Hoogen, F.H., Ropers, H.H., et al. (1995). Autosomal dominant and recessive osteochondrodysplasias associated with the COL11A2 locus. Cell 80, 431-437. 10.1016/0092-8674(95)90493-x.
[S48] Ahmad, N.N., Ala-Kokko, L., Knowlton, R.G., Jimenez, S.A., Weaver, E.J., Maguire, J.I., Tasman, W., and Prockop, D.J. (1991). Stop codon in the procollagen II gene (COL2A1) in a family with the Stickler syndrome (arthro-ophthalmopathy). Proc Natl Acad Sci U S A 88, 6624-6627. 10.1073/pnas.88.15.6624.
[S49] Khetarpal, U., Robertson, N.G., Yoo, T.J., and Morton, C.C. (1994). Expression and localization of COL2A1 mRNA and type II collagen in human fetal cochlea. Hear Res 79, 59-73. 10.1016/0378-5955(94)90127-9.
[S50] Barozzi, S., Soi, D., Intieri, E., Giani, M., Aldè, M., Tonon, E., Signorini, L., Renieri, A., Fallerini, C., Perin, P., et al. (2020). Vestibular and audiological findings in the Alport syndrome. Am J Med Genet A 182, 2345-2358. 10.1002/ajmg.a.61796.
[S51] Nagel, M., Nagorka, S., and Gross, O. (2005). Novel COL4A5, COL4A4, and COL4A3 mutations in Alport syndrome. Hum Mutat 26, 60. 10.1002/humu.9349.
[S52] Mochizuki, T., Lemmink, H.H., Mariyama, M., Antignac, C., Gubler, M.C., Pirson, Y., Verellen-Dumoulin, C., Chan, B., Schröder, C.H., Smeets, H.J., and et al. (1994). Identification of mutations in the alpha 3(IV) and alpha 4(IV) collagen genes in autosomal recessive Alport syndrome. Nat Genet 8, 77-81. 10.1038/ng0994-77.
[S53] Barker, D.F., Hostikka, S.L., Zhou, J., Chow, L.T., Oliphant, A.R., Gerken, S.C., Gregory, M.C., Skolnick, M.H., Atkin, C.L., and Tryggvason, K. (1990). Identification of mutations in the COL4A5 collagen gene in Alport syndrome. Science 248, 1224-1227. 10.1126/science.2349482.
[S54] Rost, S., Bach, E., Neuner, C., Nanda, I., Dysek, S., Bittner, R.E., Keller, A., Bartsch, O., Mlynski, R., Haaf, T., et al. (2014). Novel form of X-linked nonsyndromic hearing loss with cochlear malformation caused by a mutation in the type IV collagen gene COL4A6. Eur J Hum Genet 22, 208-215. 10.1038/ejhg.2013.108.
[S55] Van Camp, G., Snoeckx, R.L., Hilgert, N., van den Ende, J., Fukuoka, H., Wagatsuma, M., Suzuki, H., Smets, R.M., Vanhoenacker, F., Declau, F., et al. (2006). A new autosomal recessive form of Stickler syndrome is caused by a mutation in the COL9A1 gene. Am J Hum Genet 79, 449-457. 10.1086/506478.
[S56] Sivakumaran, T.A., Resendes, B.L., Robertson, N.G., Giersch, A.B., and Morton, C.C. (2006). Characterization of an abundant COL9A1 transcript in the cochlea with a novel 3' UTR: Expression studies and detection of miRNA target sequence. J Assoc Res Otolaryngol 7, 160-172. 10.1007/s10162-006-0032-0.
[S57] Johnson Chacko, L., Lahlou, H., Steinacher, C., Assou, S., Messat, Y., Dudás, J., Edge, A., Crespo, B., Crosier, M., Sergi, C., et al. (2021). Transcriptome-Wide Analysis Reveals a Role for Extracellular Matrix and Integrin Receptor Genes in Otic Neurosensory Differentiation from Human iPSCs. Int J Mol Sci 22. 10.3390/ijms221910849.
[S58] Abe, S., Katagiri, T., Saito-Hisaminato, A., Usami, S., Inoue, Y., Tsunoda, T., and Nakamura, Y. (2003). Identification of CRYM as a candidate responsible for nonsyndromic deafness, through cDNA microarray analysis of human cochlear and vestibular tissues. Am J Hum Genet 72, 73-82. 10.1086/345398.
[S59] Grati, M., Chakchouk, I., Ma, Q., Bensaid, M., Desmidt, A., Turki, N., Yan, D., Baanannou, A., Mittal, R., Driss, N., et al. (2015). A missense mutation in DCDC2 causes human recessive deafness DFNB66, likely by interfering with sensory hair cell and supporting cell cilia length regulation. Hum Mol Genet 24, 2482-2491. 10.1093/hmg/ddv009.
[S60] Cheng, J., Zhu, Y., He, S., Lu, Y., Chen, J., Han, B., Petrillo, M., Wrzeszczynski, K.O., Yang, S., Dai, P., et al. (2011). Functional mutation of SMAC/DIABLO, encoding a mitochondrial proapoptotic protein, causes human progressive hearing loss DFNA64. Am J Hum Genet 89, 56-66. 10.1016/j.ajhg.2011.05.027.
[S61] Lynch, E.D., Lee, M.K., Morrow, J.E., Welcsh, P.L., León, P.E., and King, M.C. (1997). Nonsyndromic deafness DFNA1 associated with mutation of a human homolog of the Drosophila gene diaphanous. Science 278, 1315-1318.
[S62] Ninoyu, Y., Sakaguchi, H., Lin, C., Suzuki, T., Hirano, S., Hisa, Y., Saito, N., and Ueyama, T. (2020). The integrity of cochlear hair cells is established and maintained through the localization of Dia1 at apical junctional complexes and stereocilia. Cell Death Dis 11, 536. 10.1038/s41419-020-02743-z.
[S63] Kim, T.B., Isaacson, B., Sivakumaran, T.A., Starr, A., Keats, B.J., and Lesperance, M.M. (2004). A gene responsible for autosomal dominant auditory neuropathy (AUNA1) maps to 13q14-21. J Med Genet 41, 872-876. 10.1136/jmg.2004.020628.
[S64] Schoen, C.J., Emery, S.B., Thorne, M.C., Ammana, H.R., Sliwerska, E., Arnett, J., Hortsch, M., Hannan, F., Burmeister, M., and Lesperance, M.M. (2010). Increased activity of Diaphanous homolog 3 (DIAPH3)/diaphanous causes hearing defects in humans with auditory neuropathy and in Drosophila. Proc Natl Acad Sci U S A 107, 13396-13401. 10.1073/pnas.1003027107.
[S65] Chen, D.Y., Liu, X.F., Lin, X.J., Zhang, D., Chai, Y.C., Yu, D.H., Sun, C.L., Wang, X.L., Zhu, W.D., Chen, Y., et al. (2017). A dominant variant in DMXL2 is linked to nonsyndromic hearing loss. Genet Med 19, 553-558. 10.1038/gim.2016.142.
[S66] Edery, P., Attié, T., Amiel, J., Pelet, A., Eng, C., Hofstra, R.M., Martelli, H., Bidaud, C., Munnich, A., and Lyonnet, S. (1996). Mutation of the endothelin-3 gene in the Waardenburg-Hirschsprung disease (Shah-Waardenburg syndrome). Nat Genet 12, 442-444. 10.1038/ng0496-442.
[S67] Attié, T., Till, M., Pelet, A., Amiel, J., Edery, P., Boutrand, L., Munnich, A., and Lyonnet, S. (1995). Mutation of the endothelin-receptor B gene in Waardenburg-Hirschsprung disease. Hum Mol Genet 4, 2407-2409. 10.1093/hmg/4.12.2407.
[S68] Jaworek, T.J., Richard, E.M., Ivanova, A.A., Giese, A.P., Choo, D.I., Khan, S.N., Riazuddin, S., Kahn, R.A., and Riazuddin, S. (2013). An alteration in ELMOD3, an Arl2 GTPase-activating protein, is associated with hearing impairment in humans. PLoS Genet 9, e1003774. 10.1371/journal.pgen.1003774.
[S69] Li, W., Feng, Y., Chen, A., Li, T., Huang, S., Liu, J., Liu, X., Liu, Y., Gao, J., Yan, D., et al. (2019). Elmod3 knockout leads to progressive hearing loss and abnormalities in cochlear hair cell stereocilia. Hum Mol Genet 28, 4103-4112. 10.1093/hmg/ddz240.

[S70] Behlouli, A., Bonnet, C., Abdi, S., Bouaita, A., Lelli, A., Hardelin, J.P., Schietroma, C., Rous, Y., Louha, M., Cheknane, A., et al. (2014). EPS8, encoding an actin-binding protein of cochlear hair cell stereocilia, is a new causal gene for autosomal recessive profound deafness. Orphanet J Rare Dis 9, 55. 10.1186/1750-1172-9-55.
[S71] Dahmani, M., Ammar-Khodja, F., Bonnet, C., Lefèvre, G.M., Hardelin, J.P., Ibrahim, H., Mallek, Z., and Petit, C. (2015). EPS8L2 is a new causal gene for childhood onset autosomal recessive progressive hearing loss. Orphanet J Rare Dis 10, 96. 10.1186/s13023-015-0316-8.
[S72] Furness, D.N., Johnson, S.L., Manor, U., Rüttiger, L., Tocchetti, A., Offenhauser, N., Olt, J., Goodyear, R.J., Vijayakumar, S., Dai, Y., et al. (2013). Progressive hearing loss and gradual deterioration of sensory hair bundles in the ears of mice lacking the actin-binding protein Eps8L2. Proc Natl Acad Sci U S A 110, 13898-13903. 10.1073/pnas.1304644110.
[S73] Chatzispyrou, I.A., Alders, M., Guerrero-Castillo, S., Zapata Perez, R., Haagmans, M.A., Mouchiroud, L., Koster, J., Ofman, R., Baas, F., Waterham, H.R., et al. (2017). A homozygous missense mutation in ERAL1, encoding a mitochondrial rRNA chaperone, causes Perrault syndrome. Hum Mol Genet 26, 2541-2550. 10.1093/hmg/ddx152.
[S74] Naz, S., Griffith, A.J., Riazuddin, S., Hampton, L.L., Battey, J.F., Jr., Khan, S.N., Riazuddin, S., Wilcox, E.R., and Friedman, T.B. (2004). Mutations of ESPN cause autosomal recessive deafness and vestibular dysfunction. J Med Genet 41, 591-595. 10.1136/jmg.2004.018523.
[S75] Rohacek, A.M., Bebee, T.W., Tilton, R.K., Radens, C.M., McDermott-Roe, C., Peart, N., Kaur, M., Zaykaner, M., Cieply, B., Musunuru, K., et al. (2017). ESRP1 Mutations Cause Hearing Loss due to Defects in Alternative Splicing that Disrupt Cochlear Development. Dev Cell 43, 318-331.e315. 10.1016/j.devcel.2017.09.026.
[S76] Collin, R.W., Kalay, E., Tariq, M., Peters, T., van der Zwaag, B., Venselaar, H., Oostrik, J., Lee, K., Ahmed, Z.M., Caylan, R., et al. (2008). Mutations of ESRRB encoding estrogen-related receptor beta cause autosomal-recessive nonsyndromic hearing impairment DFNB35. Am J Hum Genet 82, 125-138. 10.1016/j.ajhg.2007.09.008.
[S77] Abdelhak, S., Kalatzis, V., Heilig, R., Compain, S., Samson, D., Vincent, C., Weil, D., Cruaud, C., Sahly, I., Leibovici, M., et al. (1997). A human homologue of the Drosophila eyes absent gene underlies branchio-oto-renal (BOR) syndrome and identifies a novel gene family. Nat Genet 15, 157-164. 10.1038/ng0297-157.
[S78] Wayne, S., Robertson, N.G., DeClau, F., Chen, N., Verhoeven, K., Prasad, S., Tranebjärg, L., Morton, C.C., Ryan, A.F., Van Camp, G., and Smith, R.J. (2001). Mutations in the transcriptional activator EYA4 cause late-onset deafness at the DFNA10 locus. Hum Mol Genet 10, 195-200. 10.1093/hmg/10.3.195.
[S79] Matsuzaki, S., Hosoya, M., Okano, H., Fujioka, M., and Ogawa, K. (2018). Expression pattern of EYA4 in the common marmoset (Callithrix jacchus) cochlea. Neurosci Lett 662, 185-188. 10.1016/j.neulet.2017.10.030.
[S80] Yang, T., Vidarsson, H., Rodrigo-Blomqvist, S., Rosengren, S.S., Enerback, S., and Smith, R.J. (2007). Transcriptional control of SLC26A4 is involved in Pendred syndrome and nonsyndromic enlargement of vestibular aqueduct (DFNB4). Am J Hum Genet 80, 1055-1063. 10.1086/518314.
[S81] Vidarsson, H., Westergren, R., Heglind, M., Blomqvist, S.R., Breton, S., and Enerbäck, S. (2009). The forkhead transcription factor Foxi1 is a master regulator of vacuolar H-ATPase proton pump subunits in the inner ear, kidney and epididymis. PLoS One 4, e4471. 10.1371/journal.pone.0004471.
[S82] Yousaf, R., Ahmed, Z.M., Giese, A.P., Morell, R.J., Lagziel, A., Dabdoub, A., Wilcox, E.R., Riazuddin, S., Friedman, T.B., and Riazuddin, S. (2018). Modifier variant of METTL13 suppresses human GAB1-associated profound deafness. J Clin Invest 128, 1509-1522. 10.1172/jci97350.
[S83] Chen, T., Rohacek, A.M., Caporizzo, M., Nankali, A., Smits, J.J., Oostrik, J., Lanting, C.P., Kücük, E., Gilissen, C., van de Kamp, J.M., et al. (2021). Cochlear supporting cells require GAS2 for cytoskeletal architecture and hearing. Dev Cell 56, 1526-1540.e1527. 10.1016/j.devcel.2021.04.017.
[S84] Charizopoulou, N., Lelli, A., Schraders, M., Ray, K., Hildebrand, M.S., Ramesh, A., Srisailapathy, C.R., Oostrik, J., Admiraal, R.J., Neely, H.R., et al. (2011). Gipc3 mutations associated with audiogenic seizures and sensorineural hearing loss in mouse and human. Nat Commun 2, 201. 10.1038/ncomms1200.
[S85] Rehman, A.U., Gul, K., Morell, R.J., Lee, K., Ahmed, Z.M., Riazuddin, S., Ali, R.A., Shahzad, M., Jaleel, A.U., Andrade, P.B., et al. (2011). Mutations of GIPC3 cause nonsyndromic hearing loss DFNB72 but not DFNB81 that also maps to chromosome 19p. Hum Genet 130, 759-765. 10.1007/s00439-011-1018-5.
[S86] Kelsell, D.P., Dunlop, J., Stevens, H.P., Lench, N.J., Liang, J.N., Parry, G., Mueller, R.F., and Leigh, I.M. (1997). Connexin 26 mutations in hereditary non-syndromic sensorineural deafness. Nature 387, 80-83. 10.1038/387080a0.
[S87] Dodson, K.M., Blanton, S.H., Welch, K.O., Norris, V.W., Nuzzo, R.L., Wegelin, J.A., Marin, R.S., Nance, W.E., Pandya, A., and Arnos, K.S. (2011). Vestibular dysfunction in DFNB1 deafness. Am J Med Genet A 155a, 993-1000. 10.1002/ajmg.a.33828.
[S88] Xia, J.H., Liu, C.Y., Tang, B.S., Pan, Q., Huang, L., Dai, H.P., Zhang, B.R., Xie, W., Hu, D.X., Zheng, D., et al. (1998). Mutations in the gene encoding gap junction protein beta-3 associated with autosomal dominant hearing impairment. Nat Genet 20, 370-373. 10.1038/3845.
[S89] López-Bigas, N., Olivé, M., Rabionet, R., Ben-David, O., Martínez-Matos, J.A., Bravo, O., Banchs, I., Volpini, V., Gasparini, P., Avraham, K.B., et al. (2001). Connexin 31 (GJB3) is expressed in the peripheral and auditory nerves and causes neuropathy and hearing impairment. Hum Mol Genet 10, 947-952. 10.1093/hmg/10.9.947.
[S90] Grifa, A., Wagner, C.A., D'Ambrosio, L., Melchionda, S., Bernardi, F., Lopez-Bigas, N., Rabionet, R., Arbones, M., Monica, M.D., Estivill, X., et al. (1999). Mutations in GJB6 cause nonsyndromic autosomal dominant deafness at DFNA3 locus. Nat Genet 23, 16-18. 10.1038/12612.
[S91] Walsh, T., Shahin, H., Elkan-Miller, T., Lee, M.K., Thornton, A.M., Roeb, W., Abu Rayyan, A., Loulus, S., Avraham, K.B., King, M.C., and Kanaan, M. (2010). Whole exome sequencing and homozygosity mapping identify mutation in the cell polarity protein GPSM2 as the cause of nonsyndromic hearing loss DFNB82. Am J Hum Genet 87, 90-94. 10.1016/j.ajhg.2010.05.010.

[S92] Li, C., Bademci, G., Subasioglu, A., Diaz-Horta, O., Zhu, Y., Liu, J., Mitchell, T.G., Abad, C., Seyhan, S., Duman, D., et al. (2019). Dysfunction of GRAP, encoding the GRB2-related adaptor protein, is linked to sensorineural hearing loss. Proc Natl Acad Sci U S A 116, 1347-1352. 10.1073/pnas.1810951116.
[S93] Hosoya, M., Fujioka, M., Ogawa, K., and Okano, H. (2016). Distinct Expression Patterns Of Causative Genes Responsible For Hereditary Progressive Hearing Loss In Non-Human Primate Cochlea. Sci Rep 6, 22250. 10.1038/srep22250.
[S94] Peters, L.M., Anderson, D.W., Griffith, A.J., Grundfast, K.M., San Agustin, T.B., Madeo, A.C., Friedman, T.B., and Morell, R.J. (2002). Mutation of a transcription factor, TFCP2L3, causes progressive autosomal dominant hearing loss, DFNA28. Hum Mol Genet 11, 2877-2885. 10.1093/hmg/11.23.2877.
[S95] Schraders, M., Lee, K., Oostrik, J., Huygen, P.L., Ali, G., Hoefsloot, L.H., Veltman, J.A., Cremers, F.P., Basit, S., Ansar, M., et al. (2010). Homozygosity mapping reveals mutations of GRXCR1 as a cause of autosomal-recessive nonsyndromic hearing impairment. Am J Hum Genet 86, 138-147. 10.1016/j.ajhg.2009.12.017.
[S96] Imtiaz, A., Kohrman, D.C., and Naz, S. (2014). A frameshift mutation in GRXCR2 causes recessively inherited hearing loss. Hum Mutat 35, 618-624. 10.1002/humu.22545.
[S97] Avenarius, M.R., Jung, J.Y., Askew, C., Jones, S.M., Hunker, K.L., Azaiez, H., Rehman, A.U., Schraders, M., Najmabadi, H., Kremer, H., et al. (2018). Grxcr2 is required for stereocilia morphogenesis in the cochlea. PLoS One 13, e0201713. 10.1371/journal.pone.0201713.
[S98] Van Laer, L., Huizing, E.H., Verstreken, M., van Zuijlen, D., Wauters, J.G., Bossuyt, P.J., Van de Heyning, P., McGuirt, W.T., Smith, R.J., Willems, P.J., et al. (1998). Nonsyndromic hearing impairment is associated with a mutation in DFNA5. Nat Genet 20, 194-197. 10.1038/2503.
[S99] Puffenberger, E.G., Jinks, R.N., Sougnez, C., Cibulskis, K., Willert, R.A., Achilly, N.P., Cassidy, R.P., Fiorentini, C.J., Heiken, K.F., Lawrence, J.J., et al. (2012). Genetic mapping and exome sequencing identify variants associated with five novel diseases. PLoS One 7, e28936. 10.1371/journal.pone.0028936.
[S100] Castiglione, A., and Möller, C. (2022). Usher Syndrome. Audiol Res 12, 42-65. 10.3390/audiolres12010005.
[S101] Pierce, S.B., Chisholm, K.M., Lynch, E.D., Lee, M.K., Walsh, T., Opitz, J.M., Li, W., Klevit, R.E., and King, M.C. (2011). Mutations in mitochondrial histidyl tRNA synthetase HARS2 cause ovarian dysgenesis and sensorineural hearing loss of Perrault syndrome. Proc Natl Acad Sci U S A 108, 6543-6548. 10.1073/pnas.1103471108.
[S102] Schultz, J.M., Khan, S.N., Ahmed, Z.M., Riazuddin, S., Waryah, A.M., Chhatre, D., Starost, M.F., Ploplis, B., Buckley, S., Velásquez, D., et al. (2009). Noncoding mutations of HGF are associated with nonsyndromic hearing loss, DFNB39. Am J Hum Genet 85, 25-39. 10.1016/j.ajhg.2009.06.003.
[S103] Azaiez, H., Decker, A.R., Booth, K.T., Simpson, A.C., Shearer, A.E., Huygen, P.L., Bu, F., Hildebrand, M.S., Ranum, P.T., Shibata, S.B., et al. (2015). HOMER2, a stereociliary scaffolding protein, is essential for normal hearing in humans and mice. PLoS Genet 11, e1005137. 10.1371/journal.pgen.1005137.
[S104] Pierce, S.B., Walsh, T., Chisholm, K.M., Lee, M.K., Thornton, A.M., Fiumara, A., Opitz, J.M., Levy-Lahad, E., Klevit, R.E., and King, M.C. (2010). Mutations in the DBP-deficiency protein HSD17B4 cause ovarian dysgenesis, hearing loss, and ataxia of Perrault Syndrome. Am J Hum Genet 87, 282-288. 10.1016/j.ajhg.2010.07.007.
[S105] Gao, X., Yuan, Y.Y., Lin, Q.F., Xu, J.C., Wang, W.Q., Qiao, Y.H., Kang, D.Y., Bai, D., Xin, F., Huang, S.S., et al. (2018). Mutation of IFNLR1, an interferon lambda receptor 1, is associated with autosomal-dominant non-syndromic hearing loss. J Med Genet 55, 298-306. 10.1136/jmedgenet-2017-104954.
[S106] Borck, G., Ur Rehman, A., Lee, K., Pogoda, H.M., Kakar, N., von Ameln, S., Grillet, N., Hildebrand, M.S., Ahmed, Z.M., Nürnberg, G., et al. (2011). Loss-of-function mutations of ILDR1 cause autosomal-recessive hearing impairment DFNB42. Am J Hum Genet 88, 127-137. 10.1016/j.ajhg.2010.12.011.
[S107] Santos-Cortez, R.L., Lee, K., Azeem, Z., Antonellis, P.J., Pollock, L.M., Khan, S., Irfanullah, Andrade-Elizondo, P.B., Chiu, I., Adams, M.D., et al. (2013). Mutations in KARS, encoding lysyl-tRNA synthetase, cause autosomal-recessive nonsyndromic hearing impairment DFNB89. Am J Hum Genet 93, 132-140. 10.1016/j.ajhg.2013.05.018.
[S108] Tyson, J., Tranebjaerg, L., Bellman, S., Wren, C., Taylor, J.F., Bathen, J., Aslaksen, B., Sørland, S.J., Lund, O., Malcolm, S., et al. (1997). IsK and KvLQT1: mutation in either of the two subunits of the slow component of the delayed rectifier potassium channel can cause Jervell and Lange-Nielsen syndrome. Hum Mol Genet 6, 2179-2185. 10.1093/hmg/6.12.2179.
[S109] Schulze-Bahr, E., Wang, Q., Wedekind, H., Haverkamp, W., Chen, Q., Sun, Y., Rubie, C., Hördt, M., Towbin, J.A., Borggrefe, M., et al. (1997). KCNE1 mutations cause jervell and Lange-Nielsen syndrome. Nat Genet 17, 267-268. 10.1038/ng1197-267.
[S110] Yang, T., Gurrola, J.G., 2nd, Wu, H., Chiu, S.M., Wangemann, P., Snyder, P.M., and Smith, R.J. (2009). Mutations of KCNJ10 together with mutations of SLC26A4 cause digenic nonsyndromic hearing loss associated with enlarged vestibular aqueduct syndrome. Am J Hum Genet 84, 651-657. 10.1016/j.ajhg.2009.04.014.
[S111] Jin, Z., Wei, D., and Järlebark, L. (2006). Developmental expression and localization of KCNJ10 K+ channels in the guinea pig inner ear. Neuroreport 17, 475-479. 10.1097/01.wnr.0000208999.25234.91.
[S112] Locher, H., De Groot, J.C., Van Iperen, L., Huisman, M.A., Frijns, J.H., and Chuva de Sousa Lopes, S.M. (2015). Development of the stria vascularis and potassium regulation in the human fetal cochlea: Insights into hereditary sensorineural hearing loss. Dev Neurobiol 75, 1219-1240. 10.1002/dneu.22279.
[S113] Kubisch, C., Schroeder, B.C., Friedrich, T., Lütjohann, B., El-Amraoui, A., Marlin, S., Petit, C., and Jentsch, T.J. (1999). KCNQ4, a novel potassium channel expressed in sensory outer hair cells, is mutated in dominant deafness. Cell 96, 437-446. 10.1016/s0092-8674(00)80556-5.
[S114] Kharkovets, T., Dedek, K., Maier, H., Schweizer, M., Khimich, D., Nouvian, R., Vardanyan, V., Leuwer, R., Moser, T., and Jentsch, T.J. (2006). Mice with altered KCNQ4 K+ channels implicate sensory outer hair cells in human progressive deafness. Embo j 25, 642-652. 10.1038/sj.emboj.7600951.

[S115] Van Beelen, E.S.A., Van der Valk, W.H., De Groot, J., Hensen, E.F., Locher, H., and Van Benthem, P.P.G. (2020). Migration and fate of vestibular melanocytes during the development of the human inner ear. Dev Neurobiol 80, 411-432. 10.1002/dneu.22786.
[S116] Neyroud, N., Tesson, F., Denjoy, I., Leibovici, M., Donger, C., Barhanin, J., Fauré, S., Gary, F., Coumel, P., Petit, C., et al. (1997). A novel mutation in the potassium channel gene KVLQT1 causes the Jervell and Lange-Nielsen cardioauditory syndrome. Nat Genet 15, 186-189. 10.1038/ng0297-186.
[S117] Pierce, S.B., Gersak, K., Michaelson-Cohen, R., Walsh, T., Lee, M.K., Malach, D., Klevit, R.E., King, M.C., and Levy-Lahad, E. (2013). Mutations in LARS2, encoding mitochondrial leucyl-tRNA synthetase, lead to premature ovarian failure and hearing loss in Perrault syndrome. Am J Hum Genet 92, 614-620. 10.1016/j.ajhg.2013.03.007.
[S118] Tlili, A., Männikkö, M., Charfedine, I., Lahmar, I., Benzina, Z., Ben Amor, M., Driss, N., Ala-Kokko, L., Drira, M., Masmoudi, S., and Ayadi, H. (2005). A novel autosomal recessive non-syndromic deafness locus, DFNB66, maps to chromosome 6p21.2-22.3 in a large Tunisian consanguineous family. Hum Hered 60, 123-128. 10.1159/000088974.
[S119] Shabbir, M.I., Ahmed, Z.M., Khan, S.Y., Riazuddin, S., Waryah, A.M., Khan, S.N., Camps, R.D., Ghosh, M., Kabra, M., Belyantseva, I.A., et al. (2006). Mutations of human TMHS cause recessively inherited non-syndromic hearing loss. J Med Genet 43, 634-640. 10.1136/jmg.2005.039834.
[S120] Kalay, E., Li, Y., Uzumcu, A., Uyguner, O., Collin, R.W., Caylan, R., Ulubil-Emiroglu, M., Kersten, F.F., Hafiz, G., van Wijk, E., et al. (2006). Mutations in the lipoma HMGIC fusion partner-like 5 (LHFPL5) gene cause autosomal recessive nonsyndromic hearing loss. Hum Mutat 27, 633-639. 10.1002/humu.20368.
[S121] Wesdorp, M., de Koning Gans, P.A.M., Schraders, M., Oostrik, J., Huynen, M.A., Venselaar, H., Beynon, A.J., van Gaalen, J., Piai, V., Voermans, N., et al. (2018). Heterozygous missense variants of LMX1A lead to nonsyndromic hearing impairment and vestibular dysfunction. Hum Genet 137, 389-400. 10.1007/s00439-018-1880-5.
[S122] Chizhikov, V.V., Iskusnykh, I.Y., Fattakhov, N., and Fritzsch, B. (2021). Lmx1a and Lmx1b are Redundantly Required for the Development of Multiple Components of the Mammalian Auditory System. Neuroscience 452, 247-264. 10.1016/j.neuroscience.2020.11.013.
[S123] Grillet, N., Schwander, M., Hildebrand, M.S., Sczaniecka, A., Kolatkar, A., Velasco, J., Webster, J.A., Kahrizi, K., Najmabadi, H., Kimberling, W.J., et al. (2009). Mutations in LOXHD1, an evolutionarily conserved stereociliary protein, disrupt hair cell function in mice and cause progressive hearing loss in humans. Am J Hum Genet 85, 328-337. 10.1016/j.ajhg.2009.07.017.
[S124] Ahmed, Z.M., Masmoudi, S., Kalay, E., Belyantseva, I.A., Mosrati, M.A., Collin, R.W., Riazuddin, S., Hmani-Aifa, M., Venselaar, H., Kawar, M.N., et al. (2008). Mutations of LRTOMT, a fusion gene with alternative reading frames, cause nonsyndromic deafness in humans. Nat Genet 40, 1335-1340. 10.1038/ng.245.
[S125] Cui, L., Zheng, J., Zhao, Q., Chen, J.R., Liu, H., Peng, G., Wu, Y., Chen, C., He, Q., Shi, H., et al. (2020). Mutations of MAP1B encoding a microtubule-associated phosphoprotein cause sensorineural hearing loss. JCI Insight 5. 10.1172/jci.insight.136046.
[S126] Riazuddin, S., Ahmed, Z.M., Fanning, A.S., Lagziel, A., Kitajiri, S., Ramzan, K., Khan, S.N., Chattaraj, P., Friedman, P.L., Anderson, J.M., et al. (2006). Tricellulin is a tight-junction protein necessary for hearing. Am J Hum Genet 79, 1040-1051. 10.1086/510022.
[S127] Gao, J., Wang, Q., Dong, C., Chen, S., Qi, Y., and Liu, Y. (2015). Whole Exome Sequencing Identified MCM2 as a Novel Causative Gene for Autosomal Dominant Nonsyndromic Deafness in a Chinese Family. PLoS One 10, e0133522. 10.1371/journal.pone.0133522.
[S128] Mujtaba, G., Schultz, J.M., Imtiaz, A., Morell, R.J., Friedman, T.B., and Naz, S. (2015). A mutation of MET, encoding hepatocyte growth factor receptor, is associated with human DFNB97 hearing loss. J Med Genet 52, 548-552. 10.1136/jmedgenet-2015-103023.
[S129] Riazuddin, S., Castelein, C.M., Ahmed, Z.M., Lalwani, A.K., Mastroianni, M.A., Naz, S., Smith, T.N., Liburd, N.A., Friedman, T.B., Griffith, A.J., et al. (2000). Dominant modifier DFNM1 suppresses recessive deafness DFNB26. Nat Genet 26, 431-434. 10.1038/82558.
[S130] Mencía, A., Modamio-Høybjør, S., Redshaw, N., Morín, M., Mayo-Merino, F., Olavarrieta, L., Aguirre, L.A., del Castillo, I., Steel, K.P., Dalmay, T., et al. (2009). Mutations in the seed region of human miR-96 are responsible for nonsyndromic progressive hearing loss. Nat Genet 41, 609-613. 10.1038/ng.355.
[S131] Ushakov, K., Rudnicki, A., and Avraham, K.B. (2013). MicroRNAs in sensorineural diseases of the ear. Front Mol Neurosci 6, 52. 10.3389/fnmol.2013.00052.
[S132] Lewis, M.A., Quint, E., Glazier, A.M., Fuchs, H., De Angelis, M.H., Langford, C., van Dongen, S., Abreu-Goodger, C., Piipari, M., Redshaw, N., et al. (2009). An ENU-induced mutation of miR-96 associated with progressive hearing loss in mice. Nat Genet 41, 614-618. 10.1038/ng.369.
[S133] Tassabehji, M., Newton, V.E., and Read, A.P. (1994). Waardenburg syndrome type 2 caused by mutations in the human microphthalmia (MITF) gene. Nat Genet 8, 251-255. 10.1038/ng1194-251.
[S134] Wesdorp, M., Murillo-Cuesta, S., Peters, T., Celaya, A.M., Oonk, A., Schraders, M., Oostrik, J., Gomez-Rosas, E., Beynon, A.J., Hartel, B.P., et al. (2018). MPZL2, Encoding the Epithelial Junctional Protein Myelin Protein Zero-like 2, Is Essential for Hearing in Man and Mouse. Am J Hum Genet 103, 74-88. 10.1016/j.ajhg.2018.05.011.
[S135] Waryah, A.M., Rehman, A., Ahmed, Z.M., Bashir, Z.H., Khan, S.Y., Zafar, A.U., Riazuddin, S., Friedman, T.B., and Riazuddin, S. (2009). DFNB74, a novel autosomal recessive nonsyndromic hearing impairment locus on chromosome 12q14.2-q15. Clin Genet 76, 270-275. 10.1111/j.1399-0004.2009.01209.x.
[S136] Donaudy, F., Snoeckx, R., Pfister, M., Zenner, H.P., Blin, N., Di Stazio, M., Ferrara, A., Lanzara, C., Ficarella, R., Declau, F., et al. (2004). Nonmuscle myosin heavy-chain gene MYH14 is expressed in cochlea and mutated in patients affected by autosomal dominant hearing impairment (DFNA4). Am J Hum Genet 74, 770-776. 10.1086/383285.

[S137] Lalwani, A.K., Goldstein, J.A., Kelley, M.J., Luxford, W., Castelein, C.M., and Mhatre, A.N. (2000). Human nonsyndromic hereditary deafness DFNA17 is due to a mutation in nonmuscle myosin MYH9. Am J Hum Genet 67, 1121-1128. 10.1016/s0002-9297(07)62942-5.
[S138] Mhatre, A.N., Li, Y., Atkin, G., Maghnouj, A., and Lalwani, A.K. (2006). Expression of Myh9 in the mammalian cochlea: localization within the stereocilia. J Neurosci Res 84, 809-818. 10.1002/jnr.20993.
[S139] Wang, A., Liang, Y., Fridell, R.A., Probst, F.J., Wilcox, E.R., Touchman, J.W., Morton, C.C., Morell, R.J., Noben-Trauth, K., Camper, S.A., and Friedman, T.B. (1998). Association of unconventional myosin MYO15 mutations with human nonsyndromic deafness DFNB3. Science 280, 1447-1451. 10.1126/science.280.5368.1447.
[S140] Walsh, T., Walsh, V., Vreugde, S., Hertzano, R., Shahin, H., Haika, S., Lee, M.K., Kanaan, M., King, M.C., and Avraham, K.B. (2002). From flies' eyes to our ears: mutations in a human class III myosin cause progressive nonsyndromic hearing loss DFNB30. Proc Natl Acad Sci U S A 99, 7518-7523. 10.1073/pnas.102091699.
[S141] Grati, M., Yan, D., Raval, M.H., Walsh, T., Ma, Q., Chakchouk, I., Kannan-Sundhari, A., Mittal, R., Masmoudi, S., Blanton, S.H., et al. (2016). MYO3A Causes Human Dominant Deafness and Interacts with Protocadherin 15-CD2 Isoform. Hum Mutat 37, 481-487. 10.1002/humu.22961.
[S142] Ahmed, Z.M., Riazuddin, S., Ahmad, J., Bernstein, S.L., Guo, Y., Sabar, M.F., Sieving, P., Riazuddin, S., Griffith, A.J., Friedman, T.B., et al. (2003). PCDH15 is expressed in the neurosensory epithelium of the eye and ear and mutant alleles are responsible for both USH1F and DFNB23. Hum Mol Genet 12, 3215-3223. 10.1093/hmg/ddg358.
[S143] Liu, X.Z., Walsh, J., Mburu, P., Kendrick-Jones, J., Cope, M.J., Steel, K.P., and Brown, S.D. (1997). Mutations in the myosin VIIA gene cause non-syndromic recessive deafness. Nat Genet 16, 188-190. 10.1038/ng0697-188.
[S144] Weil, D., Küssel, P., Blanchard, S., Lévy, G., Levi-Acobas, F., Drira, M., Ayadi, H., and Petit, C. (1997). The autosomal recessive isolated deafness, DFNB2, and the Usher 1B syndrome are allelic defects of the myosin-VIIA gene. Nat Genet 16, 191-193. 10.1038/ng0697-191.
[S145] Wafa, T.T., Faridi, R., King, K.A., Zalewski, C., Yousaf, R., Schultz, J.M., Morell, R.J., Muskett, J., Turriff, A., Tsilou, E., et al. (2021). Vestibular phenotype-genotype correlation in a cohort of 90 patients with Usher syndrome. Clin Genet 99, 226-235. 10.1111/cge.13868.
[S146] Simon, M., Richard, E.M., Wang, X., Shahzad, M., Huang, V.H., Qaiser, T.A., Potluri, P., Mahl, S.E., Davila, A., Nazli, S., et al. (2015). Mutations of human NARS2, encoding the mitochondrial asparaginyl-tRNA synthetase, cause nonsyndromic deafness and Leigh syndrome. PLoS Genet 11, e1005097. 10.1371/journal.pgen.1005097.
[S147] Berger, W., Meindl, A., van de Pol, T.J., Cremers, F.P., Ropers, H.H., Döerner, C., Monaco, A., Bergen, A.A., Lebo, R., Warburg, M., and et al. (1992). Isolation of a candidate gene for Norrie disease by positional cloning. Nat Genet 1, 199-203. 10.1038/ng0692-199.
[S148] Chen, Z.Y., Hendriks, R.W., Jobling, M.A., Powell, J.F., Breakefield, X.O., Sims, K.B., and Craig, I.W. (1992). Isolation and characterization of a candidate gene for Norrie disease. Nat Genet 1, 204-208. 10.1038/ng0692-204.
[S149] Hayashi, Y., Chiang, H., Tian, C., Indzhykulian, A.A., and Edge, A.S.B. (2021). Norrie disease protein is essential for cochlear hair cell maturation. Proc Natl Acad Sci U S A 118. 10.1073/pnas.2106369118.
[S150] Nakanishi, H., Kawashima, Y., Kurima, K., Chae, J.J., Ross, A.M., Pinto-Patarroyo, G., Patel, S.K., Muskett, J.A., Ratay, J.S., Chattaraj, P., et al. (2017). NLRP3 mutation and cochlear autoinflammation cause syndromic and nonsyndromic hearing loss DFNA34 responsive to anakinra therapy. Proc Natl Acad Sci U S A 114, E7766-e7775. 10.1073/pnas.1702946114.
[S151] Xing, G., Yao, J., Wu, B., Liu, T., Wei, Q., Liu, C., Lu, Y., Chen, Z., Zheng, H., Yang, X., and Cao, X. (2015). Identification of OSBPL2 as a novel candidate gene for progressive nonsyndromic hearing loss by whole-exome sequencing. Genet Med 17, 210-218. 10.1038/gim.2014.90.
[S152] Thoenes, M., Zimmermann, U., Ebermann, I., Ptok, M., Lewis, M.A., Thiele, H., Morlot, S., Hess, M.M., Gal, A., Eisenberger, T., et al. (2015). OSBPL2 encodes a protein of inner and outer hair cell stereocilia and is mutated in autosomal dominant hearing loss (DFNA67). Orphanet J Rare Dis 10, 15. 10.1186/s13023-015-0238-5.
[S153] Zwaenepoel, I., Mustapha, M., Leibovici, M., Verpy, E., Goodyear, R., Liu, X.Z., Nouaille, S., Nance, W.E., Kanaan, M., Avraham, K.B., et al. (2002). Otoancorin, an inner ear protein restricted to the interface between the apical surface of sensory epithelia and their overlying acellular gels, is defective in autosomal recessive deafness DFNB22. Proc Natl Acad Sci U S A 99, 6240-6245. 10.1073/pnas.082515999.
[S154] Lukashkin, A.N., Legan, P.K., Weddell, T.D., Lukashkina, V.A., Goodyear, R.J., Welstead, L.J., Petit, C., Russell, I.J., and Richardson, G.P. (2012). A mouse model for human deafness DFNB22 reveals that hearing impairment is due to a loss of inner hair cell stimulation. Proc Natl Acad Sci U S A 109, 19351-19356. 10.1073/pnas.1210159109.
[S155] Yasunaga, S., Grati, M., Cohen-Salmon, M., El-Amraoui, A., Mustapha, M., Salem, N., El-Zir, E., Loiselet, J., and Petit, C. (1999). A mutation in OTOF, encoding otoferlin, a FER-1-like protein, causes DFNB9, a nonsyndromic form of deafness. Nat Genet 21, 363-369. 10.1038/7693.
[S156] Santarelli, R., Scimemi, P., Costantini, M., Domínguez-Ruiz, M., Rodríguez-Ballesteros, M., and Del Castillo, I. (2021). Cochlear Synaptopathy due to Mutations in OTOF Gene May Result in Stable Mild Hearing Loss and Severe Impairment of Speech Perception. Ear Hear 42, 1627-1639. 10.1097/aud.0000000000001052.
[S157] Schraders, M., Ruiz-Palmero, L., Kalay, E., Oostrik, J., del Castillo, F.J., Sezgin, O., Beynon, A.J., Strom, T.M., Pennings, R.J., Zazo Seco, C., et al. (2012). Mutations of the gene encoding otogelin are a cause of autosomal-recessive nonsyndromic moderate hearing impairment. Am J Hum Genet 91, 883-889. 10.1016/j.ajhg.2012.09.012.
[S158] Yariz, K.O., Duman, D., Zazo Seco, C., Dallman, J., Huang, M., Peters, T.A., Sirmaci, A., Lu, N., Schraders, M., Skromne, I., et al. (2012). Mutations in OTOGL, encoding the inner ear protein otogelin-like, cause moderate sensorineural hearing loss. Am J Hum Genet 91, 872-882. 10.1016/j.ajhg.2012.09.011.
[S159] Yan, D., Zhu, Y., Walsh, T., Xie, D., Yuan, H., Sirmaci, A., Fujikawa, T., Wong, A.C., Loh, T.L., Du, L., et al. (2013). Mutation of the ATP-gated P2X(2) receptor leads to progressive hearing loss and increased susceptibility to noise. Proc Natl Acad Sci U S A 110, 2228-2233. 10.1073/pnas.1222285110.

[S160] Järlebark, L.E., Housley, G.D., Raybould, N.P., Vlajkovic, S., and Thorne, P.R. (2002). ATP-gated ion channels assembled from P2X2 receptor subunits in the mouse cochlea. Neuroreport 13, 1979-1984. 10.1097/00001756-200210280-00030.
[S161] Tassabehji, M., Read, A.P., Newton, V.E., Harris, R., Balling, R., Gruss, P., and Strachan, T. (1992). Waardenburg's syndrome patients have mutations in the human homologue of the Pax-3 paired box gene. Nature 355, 635-636. 10.1038/355635a0.
[S162] Zlotogora, J. (1995). X-linked albinism-deafness syndrome and Waardenburg syndrome type II: a hypothesis. Am J Med Genet 59, 386-387. 10.1002/ajmg.1320590321.
[S163] Kim, H., Ankamreddy, H., Lee, D.J., Kong, K.A., Ko, H.W., Kim, M.H., and Bok, J. (2014). Pax3 function is required specifically for inner ear structures with melanogenic fates. Biochem Biophys Res Commun 445, 608-614. 10.1016/j.bbrc.2014.02.047.
[S164] Ahmed, Z.M., Riazuddin, S., Bernstein, S.L., Ahmed, Z., Khan, S., Griffith, A.J., Morell, R.J., Friedman, T.B., Riazuddin, S., and Wilcox, E.R. (2001). Mutations of the protocadherin gene PCDH15 cause Usher syndrome type 1F. Am J Hum Genet 69, 25-34. 10.1086/321277.
[S165] Alagramam, K.N., Yuan, H., Kuehn, M.H., Murcia, C.L., Wayne, S., Srisailpathy, C.R., Lowry, R.B., Knaus, R., Van Laer, L., Bernier, F.P., et al. (2001). Mutations in the novel protocadherin PCDH15 cause Usher syndrome type 1F. Hum Mol Genet 10, 1709-1718. 10.1093/hmg/10.16.1709.
[S166] Webb, S.W., Grillet, N., Andrade, L.R., Xiong, W., Swarthout, L., Della Santina, C.C., Kachar, B., and Müller, U. (2011). Regulation of PCDH15 function in mechanosensory hair cells by alternative splicing of the cytoplasmic domain. Development 138, 1607-1617. 10.1242/dev.060061.
[S167] Wang, L., Feng, Y., Yan, D., Qin, L., Grati, M., Mittal, R., Li, T., Sundhari, A.K., Liu, Y., Chapagain, P., et al. (2018). A dominant variant in the PDE1C gene is associated with nonsyndromic hearing loss. Hum Genet 137, 437-446. 10.1007/s00439-018-1895-y.
[S168] Booth, K.T., Azaiez, H., Kahrizi, K., Simpson, A.C., Tollefson, W.T., Sloan, C.M., Meyer, N.C., Babanejad, M., Ardalani, F., Arzhangi, S., et al. (2015). PDZD7 and hearing loss: More than just a modifier. Am J Med Genet A 167a, 2957-2965. 10.1002/ajmg.a.37274.
[S169] Chen, Q., Zou, J., Shen, Z., Zhang, W., and Yang, J. (2014). Whirlin and PDZ domain-containing 7 (PDZD7) proteins are both required to form the quaternary protein complex associated with Usher syndrome type 2. J Biol Chem 289, 36070-36088. 10.1074/jbc.M114.610535.
[S170] Delmaghani, S., del Castillo, F.J., Michel, V., Leibovici, M., Aghaie, A., Ron, U., Van Laer, L., Ben-Tal, N., Van Camp, G., Weil, D., et al. (2006). Mutations in the gene encoding pejvakin, a newly identified protein of the afferent auditory pathway, cause DFNB59 auditory neuropathy. Nat Genet 38, 770-778. 10.1038/ng1829.
[S171] Kazmierczak, M., Kazmierczak, P., Peng, A.W., Harris, S.L., Shah, P., Puel, J.L., Lenoir, M., Franco, S.J., and Schwander, M. (2017). Pejvakin, a Candidate Stereociliary Rootlet Protein, Regulates Hair Cell Function in a Cell-Autonomous Manner. J Neurosci 37, 3447-3464. 10.1523/jneurosci.2711-16.2017.
[S172] Morgan, A., Koboldt, D.C., Barrie, E.S., Crist, E.R., García García, G., Mezzavilla, M., Faletra, F., Mihalic Mosher, T., Wilson, R.K., Blanchet, C., et al. (2019). Mutations in PLS1, encoding fimbrin, cause autosomal dominant nonsyndromic hearing loss. Hum Mutat 40, 2286-2295. 10.1002/humu.23891.
[S173] Krey, J.F., Krystofiak, E.S., Dumont, R.A., Vijayakumar, S., Choi, D., Rivero, F., Kachar, B., Jones, S.M., and Barr-Gillespie, P.G. (2016). Plastin 1 widens stereocilia by transforming actin filament packing from hexagonal to liquid. J Cell Biol 215, 467-482. 10.1083/jcb.201606036.
[S174] von Ameln, S., Wang, G., Boulouiz, R., Rutherford, M.A., Smith, G.M., Li, Y., Pogoda, H.M., Nürnberg, G., Stiller, B., Volk, A.E., et al. (2012). A mutation in PNPT1, encoding mitochondrial-RNA-import protein PNPase, causes hereditary hearing loss. Am J Hum Genet 91, 919-927. 10.1016/j.ajhg.2012.09.002.
[S175] Dauwerse, J.G., Dixon, J., Seland, S., Ruivenkamp, C.A., van Haeringen, A., Hoefsloot, L.H., Peters, D.J., Boers, A.C., Daumer-Haas, C., Maiwald, R., et al. (2011). Mutations in genes encoding subunits of RNA polymerases I and III cause Treacher Collins syndrome. Nat Genet 43, 20-22. 10.1038/ng.724.
[S176] de Kok, Y.J., van der Maarel, S.M., Bitner-Glindzicz, M., Huber, I., Monaco, A.P., Malcolm, S., Pembrey, M.E., Ropers, H.H., and Cremers, F.P. (1995). Association between X-linked mixed deafness and mutations in the POU domain gene POU3F4. Science 267, 685-688. 10.1126/science.7839145.
[S177] Song, M.H., Lee, K.Y., Choi, J.Y., Bok, J., and Kim, U.K. (2012). Nonsyndromic X-linked hearing loss. Front Biosci (Elite Ed) 4, 924-933. 10.2741/e430.
[S178] Vore, A.P., Chang, E.H., Hoppe, J.E., Butler, M.G., Forrester, S., Schneider, M.C., Smith, L.L., Burke, D.W., Campbell, C.A., and Smith, R.J. (2005). Deletion of and novel missense mutation in POU3F4 in 2 families segregating X-linked nonsyndromic deafness. Arch Otolaryngol Head Neck Surg 131, 1057-1063. 10.1001/archotol.131.12.1057.
[S179] Vahava, O., Morell, R., Lynch, E.D., Weiss, S., Kagan, M.E., Ahituv, N., Morrow, J.E., Lee, M.K., Skvorak, A.B., Morton, C.C., et al. (1998). Mutation in transcription factor POU4F3 associated with inherited progressive hearing loss in humans. Science 279, 1950-1954. 10.1126/science.279.5358.1950.
[S180] van Drunen, F.J., Pauw, R.J., Collin, R.W., Kremer, H., Huygen, P.L., and Cremers, C.W. (2009). Vestibular impairment in a Dutch DFNA15 family with an L289F mutation in POU4F3. Audiol Neurootol 14, 303-307. 10.1159/000212109.
[S181] Yousaf, R., Gu, C., Ahmed, Z.M., Khan, S.N., Friedman, T.B., Riazuddin, S., Shears, S.B., and Riazuddin, S. (2018). Mutations in Diphosphoinositol-Pentakisphosphate Kinase PPIP5K2 are associated with hearing loss in human and mouse. PLoS Genet 14, e1007297. 10.1371/journal.pgen.1007297.
[S182] Liu, X., Han, D., Li, J., Han, B., Ouyang, X., Cheng, J., Li, X., Jin, Z., Wang, Y., Bitner-Glindzicz, M., et al. (2010). Loss-of-function mutations in the PRPS1 gene cause a type of nonsyndromic X-linked sensorineural deafness, DFN2. Am J Hum Genet 86, 65-71. 10.1016/j.ajhg.2009.11.015.

[S183] Eisenberger, T., Di Donato, N., Decker, C., Delle Vedove, A., Neuhaus, C., Nürnberg, G., Toliat, M., Nürnberg, P., Mürbe, D., and Bolz, H.J. (2018). A C-terminal nonsense mutation links PTPRQ with autosomal-dominant hearing loss, DFNA73. Genet Med 20, 614-621. 10.1038/gim.2017.155.
[S184] Schraders, M., Oostrik, J., Huygen, P.L., Strom, T.M., van Wijk, E., Kunst, H.P., Hoefsloot, L.H., Cremers, C.W., Admiraal, R.J., and Kremer, H. (2010). Mutations in PTPRQ are a cause of autosomal-recessive nonsyndromic hearing impairment DFNB84 and associated with vestibular dysfunction. Am J Hum Genet 86, 604-610. 10.1016/j.ajhg.2010.02.015.
[S185] Goodyear, R.J., Legan, P.K., Wright, M.B., Marcotti, W., Oganesian, A., Coats, S.A., Booth, C.J., Kros, C.J., Seifert, R.A., Bowen-Pope, D.F., and Richardson, G.P. (2003). A receptor-like inositol lipid phosphatase is required for the maturation of developing cochlear hair bundles. J Neurosci 23, 9208-9219. 10.1523/jneurosci.23-27-09208.2003.
[S186] Khan, S.Y., Ahmed, Z.M., Shabbir, M.I., Kitajiri, S., Kalsoom, S., Tasneem, S., Shayiq, S., Ramesh, A., Srisailpathy, S., Khan, S.N., et al. (2007). Mutations of the RDX gene cause nonsyndromic hearing loss at the DFNB24 locus. Hum Mutat 28, 417-423. 10.1002/humu.20469.
[S187] Kitajiri, S., Fukumoto, K., Hata, M., Sasaki, H., Katsuno, T., Nakagawa, T., Ito, J., Tsukita, S., and Tsukita, S. (2004). Radixin deficiency causes deafness associated with progressive degeneration of cochlear stereocilia. J Cell Biol 166, 559-570. 10.1083/jcb.200402007.
[S188] Nakano, Y., Kelly, M.C., Rehman, A.U., Boger, E.T., Morell, R.J., Kelley, M.W., Friedman, T.B., and Bánfi, B. (2018). Defects in the Alternative Splicing-Dependent Regulation of REST Cause Deafness. Cell 174, 536-548.e521. 10.1016/j.cell.2018.06.004.
[S189] de Bruijn, S.E., Smits, J.J., Liu, C., Lanting, C.P., Beynon, A.J., Blankevoort, J., Oostrik, J., Koole, W., de Vrieze, E., Cremers, C., et al. (2020). A RIPOR2 in-frame deletion is a frequent and highly penetrant cause of adult-onset hearing loss. J Med Genet. 10.1136/jmedgenet-2020-106863.
[S190] Diaz-Horta, O., Subasioglu-Uzak, A., Grati, M., DeSmidt, A., Foster, J., 2nd, Cao, L., Bademci, G., Tokgoz-Yilmaz, S., Duman, D., Cengiz, F.B., et al. (2014). FAM65B is a membrane-associated protein of hair cell stereocilia required for hearing. Proc Natl Acad Sci U S A 111, 9864-9868. 10.1073/pnas.1401950111.
[S191] Diaz-Horta, O., Abad, C., Cengiz, F.B., Bademci, G., Blackwelder, P., Walz, K., and Tekin, M. (2018). Ripor2 is involved in auditory hair cell stereociliary bundle structure and orientation. J Mol Med (Berl) 96, 1227-1238. 10.1007/s00109-018-1694-x.
[S192] Diaz-Horta, O., Abad, C., Sennaroglu, L., Foster, J., 2nd, DeSmidt, A., Bademci, G., Tokgoz-Yilmaz, S., Duman, D., Cengiz, F.B., Grati, M., et al. (2016). ROR1 is essential for proper innervation of auditory hair cells and hearing in humans and mice. Proc Natl Acad Sci U S A 113, 5993-5998. 10.1073/pnas.1522512113.
[S193] Santos-Cortez, R.L., Faridi, R., Rehman, A.U., Lee, K., Ansar, M., Wang, X., Morell, R.J., Isaacson, R., Belyantseva, I.A., Dai, H., et al. (2016). Autosomal-Recessive Hearing Impairment Due to Rare Missense Variants within S1PR2. Am J Hum Genet 98, 331-338. 10.1016/j.ajhg.2015.12.004.
[S194] Ingham, N.J., Carlisle, F., Pearson, S., Lewis, M.A., Buniello, A., Chen, J., Isaacson, R.L., Pass, J., White, J.K., Dawson, S.J., and Steel, K.P. (2016). S1PR2 variants associated with auditory function in humans and endocochlear potential decline in mouse. Sci Rep 6, 28964. 10.1038/srep28964.
[S195] Lu, X., Zhang, Y., Chen, L., Wang, Q., Zeng, Z., Dong, C., Qi, Y., and Liu, Y. (2020). Whole exome sequencing identifies SCD5 as a novel causative gene for autosomal dominant nonsyndromic deafness. Eur J Med Genet 63, 103855. 10.1016/j.ejmg.2020.103855.
[S196] Lalani, S.R., Safiullah, A.M., Molinari, L.M., Fernbach, S.D., Martin, D.M., and Belmont, J.W. (2004). SEMA3E mutation in a patient with CHARGE syndrome. J Med Genet 41, e94. 10.1136/jmg.2003.017640.
[S197] Sirmaci, A., Erbek, S., Price, J., Huang, M., Duman, D., Cengiz, F.B., Bademci, G., Tokgöz-Yilmaz, S., Hişmi, B., Ozdağ, H., et al. (2010). A truncating mutation in SERPINB6 is associated with autosomal-recessive nonsyndromic sensorineural hearing loss. Am J Hum Genet 86, 797-804. 10.1016/j.ajhg.2010.04.004.
[S198] Mosrati, M.A., Hammami, B., Rebeh, I.B., Ayadi, L., Dhouib, L., Ben Mahfoudh, K., Hakim, B., Charfeddine, I., Mnif, J., Ghorbel, A., and Masmoudi, S. (2011). A novel dominant mutation in SIX1, affecting a highly conserved residue, result in only auditory defects in humans. Eur J Med Genet 54, e484-488. 10.1016/j.ejmg.2011.06.001.
[S199] Ruf, R.G., Xu, P.X., Silvius, D., Otto, E.A., Beekmann, F., Muerb, U.T., Kumar, S., Neuhaus, T.J., Kemper, M.J., Raymond, R.M., Jr., et al. (2004). SIX1 mutations cause branchio-oto-renal syndrome by disruption of EYA1-SIX1-DNA complexes. Proc Natl Acad Sci U S A 101, 8090-8095. 10.1073/pnas.0308475101.
[S200] Hoskins, B.E., Cramer, C.H., Silvius, D., Zou, D., Raymond, R.M., Orten, D.J., Kimberling, W.J., Smith, R.J., Weil, D., Petit, C., et al. (2007). Transcription factor SIX5 is mutated in patients with branchio-oto-renal syndrome. Am J Hum Genet 80, 800-804. 10.1086/513322.
[S201] Mutai, H., Wasano, K., Momozawa, Y., Kamatani, Y., Miya, F., Masuda, S., Morimoto, N., Nara, K., Takahashi, S., Tsunoda, T., et al. (2020). Variants encoding a restricted carboxy-terminal domain of SLC12A2 cause hereditary hearing loss in humans. PLoS Genet 16, e1008643. 10.1371/journal.pgen.1008643.
[S202] Ruel, J., Emery, S., Nouvian, R., Bersot, T., Amilhon, B., Van Rybroek, J.M., Rebillard, G., Lenoir, M., Eybalin, M., Delprat, B., et al. (2008). Impairment of SLC17A8 encoding vesicular glutamate transporter-3, VGLUT3, underlies nonsyndromic deafness DFNA25 and inner hair cell dysfunction in null mice. Am J Hum Genet 83, 278-292. 10.1016/j.ajhg.2008.07.008.
[S203] Ben Said, M., Grati, M., Ishimoto, T., Zou, B., Chakchouk, I., Ma, Q., Yao, Q., Hammami, B., Yan, D., Mittal, R., et al. (2016). A mutation in SLC22A4 encoding an organic cation transporter expressed in the cochlea strial endothelium causes human recessive non-syndromic hearing loss DFNB60. Hum Genet 135, 513-524. 10.1007/s00439-016-1657-7.
[S204] Li, X.C., Everett, L.A., Lalwani, A.K., Desmukh, D., Friedman, T.B., Green, E.D., and Wilcox, E.R. (1998). A mutation in PDS causes non-syndromic recessive deafness. Nat Genet 18, 215-217. 10.1038/ng0398-215.

[S205] Everett, L.A., Glaser, B., Beck, J.C., Idol, J.R., Buchs, A., Heyman, M., Adawi, F., Hazani, E., Nassir, E., Baxevanis, A.D., et al. (1997). Pendred syndrome is caused by mutations in a putative sulphate transporter gene (PDS). Nat Genet 17, 411-422. 10.1038/ng1297-411.
[S206] Liu, X.Z., Ouyang, X.M., Xia, X.J., Zheng, J., Pandya, A., Li, F., Du, L.L., Welch, K.O., Petit, C., Smith, R.J., et al. (2003). Prestin, a cochlear motor protein, is defective in non-syndromic hearing loss. Hum Mol Genet 12, 1155-1162. 10.1093/hmg/ddg127.
[S207] Schraders, M., Haas, S.A., Weegerink, N.J., Oostrik, J., Hu, H., Hoefsloot, L.H., Kannan, S., Huygen, P.L., Pennings, R.J., Admiraal, R.J., et al. (2011). Next-generation sequencing identifies mutations of SMPX, which encodes the small muscle protein, X-linked, as a cause of progressive hearing impairment. Am J Hum Genet 88, 628-634. 10.1016/j.ajhg.2011.04.012.
[S208] Huebner, A.K., Gandia, M., Frommolt, P., Maak, A., Wicklein, E.M., Thiele, H., Altmüller, J., Wagner, F., Viñuela, A., Aguirre, L.A., et al. (2011). Nonsense mutations in SMPX, encoding a protein responsive to physical force, result in X-chromosomal hearing loss. Am J Hum Genet 88, 621-627. 10.1016/j.ajhg.2011.04.007.
[S209] Tu, H., Zhang, A., Fu, X., Xu, S., Bai, X., Wang, H., and Gao, J. (2021). SMPX Deficiency Causes Stereocilia Degeneration and Progressive Hearing Loss in CBA/CaJ Mice. Front Cell Dev Biol 9, 750023. 10.3389/fcell.2021.750023.
[S210] Sánchez-Martín, M., Rodríguez-García, A., Pérez-Losada, J., Sagrera, A., Read, A.P., and Sánchez-García, I. (2002). SLUG (SNAI2) deletions in patients with Waardenburg disease. Hum Mol Genet 11, 3231-3236. 10.1093/hmg/11.25.3231.
[S211] Bondurand, N., Dastot-Le Moal, F., Stanchina, L., Collot, N., Baral, V., Marlin, S., Attie-Bitach, T., Giurgea, I., Skopinski, L., Reardon, W., et al. (2007). Deletions at the SOX10 gene locus cause Waardenburg syndrome types 2 and 4. Am J Hum Genet 81, 1169-1185. 10.1086/522090.
[S212] Pingault, V., Bondurand, N., Kuhlbrodt, K., Goerich, D.E., Préhu, M.O., Puliti, A., Herbarth, B., Hermans-Borgmeyer, I., Legius, E., Matthijs, G., et al. (1998). SOX10 mutations in patients with Waardenburg-Hirschsprung disease. Nat Genet 18, 171-173. 10.1038/ng0298-171.
[S213] Chen, J., Ingham, N., Kelly, J., Jadeja, S., Goulding, D., Pass, J., Mahajan, V.B., Tsang, S.H., Nijnik, A., Jackson, I.J., et al. (2014). Spinster homolog 2 (spns2) deficiency causes early onset progressive hearing loss. PLoS Genet 10, e1004688. 10.1371/journal.pgen.1004688.
[S214] Ingham, N.J., Pearson, S.A., Vancollie, V.E., Rook, V., Lewis, M.A., Chen, J., Buniello, A., Martelletti, E., Preite, L., Lam, C.C., et al. (2019). Mouse screen reveals multiple new genes underlying mouse and human hearing loss. PLoS Biol 17, e3000194. 10.1371/journal.pbio.3000194.
[S215] Verpy, E., Masmoudi, S., Zwaenepoel, I., Leibovici, M., Hutchin, T.P., Del Castillo, I., Nouaille, S., Blanchard, S., Lainé, S., Popot, J.L., et al. (2001). Mutations in a new gene encoding a protein of the hair bundle cause non-syndromic deafness at the DFNB16 locus. Nat Genet 29, 345-349. 10.1038/ng726.
[S216] Horn, H.F., Brownstein, Z., Lenz, D.R., Shivatzki, S., Dror, A.A., Dagan-Rosenfeld, O., Friedman, L.M., Roux, K.J., Kozlov, S., Jeang, K.T., et al. (2013). The LINC complex is essential for hearing. J Clin Invest 123, 740-750. 10.1172/jci66911.
[S217] Taiber, S., Cohen, R., Yizhar-Barnea, O., Sprinzak, D., Holt, J.R., and Avraham, K.B. (2021). Neonatal AAV gene therapy rescues hearing in a mouse model of SYNE4 deafness. EMBO Mol Med 13, e13259. 10.15252/emmm.202013259.
[S218] Azaiez, H., Booth, K.T., Bu, F., Huygen, P., Shibata, S.B., Shearer, A.E., Kolbe, D., Meyer, N., Black-Ziegelbein, E.A., and Smith, R.J. (2014). TBC1D24 mutation causes autosomal-dominant nonsyndromic hearing loss. Hum Mutat 35, 819-823. 10.1002/humu.22557.
[S219] Zhang, L., Hu, L., Chai, Y., Pang, X., Yang, T., and Wu, H. (2014). A dominant mutation in the stereocilia-expressing gene TBC1D24 is a probable cause for nonsyndromic hearing impairment. Hum Mutat 35, 814-818. 10.1002/humu.22558.
[S220] Rehman, A.U., Santos-Cortez, R.L., Morell, R.J., Drummond, M.C., Ito, T., Lee, K., Khan, A.A., Basra, M.A., Wasif, N., Ayub, M., et al. (2014). Mutations in TBC1D24, a gene associated with epilepsy, also cause nonsyndromic deafness DFNB86. Am J Hum Genet 94, 144-152. 10.1016/j.ajhg.2013.12.004.
[S221] Dixon, M.J. (1996). Isolation of a deafness gene. Br J Hosp Med 55, 672-673.
[S222] Verhoeven, K., Van Laer, L., Kirschhofer, K., Legan, P.K., Hughes, D.C., Schatteman, I., Verstreken, M., Van Hauwe, P., Coucke, P., Chen, A., et al. (1998). Mutations in the human alpha-tectorin gene cause autosomal dominant non-syndromic hearing impairment. Nat Genet 19, 60-62. 10.1038/ng0598-60.
[S223] Mustapha, M., Weil, D., Chardenoux, S., Elias, S., El-Zir, E., Beckmann, J.S., Loiselet, J., and Petit, C. (1999). An alpha-tectorin gene defect causes a newly identified autosomal recessive form of sensorineural pre-lingual non-syndromic deafness, DFNB21. Hum Mol Genet 8, 409-412. 10.1093/hmg/8.3.409.
[S224] Walsh, T., Pierce, S.B., Lenz, D.R., Brownstein, Z., Dagan-Rosenfeld, O., Shahin, H., Roeb, W., McCarthy, S., Nord, A.S., Gordon, C.R., et al. (2010). Genomic duplication and overexpression of TJP2/ZO-2 leads to altered expression of apoptosis genes in progressive nonsyndromic hearing loss DFNA51. Am J Hum Genet 87, 101-109. 10.1016/j.ajhg.2010.05.011.
[S225] Kurima, K., Peters, L.M., Yang, Y., Riazuddin, S., Ahmed, Z.M., Naz, S., Arnaud, D., Drury, S., Mo, J., Makishima, T., et al. (2002). Dominant and recessive deafness caused by mutations of a novel gene, TMC1, required for cochlear hair-cell function. Nat Genet 30, 277-284. 10.1038/ng842.
[S226] Li, J., Zhao, X., Xin, Q., Shan, S., Jiang, B., Jin, Y., Yuan, H., Dai, P., Xiao, R., Zhang, Q., et al. (2015). Whole-exome sequencing identifies a variant in TMEM132E causing autosomal-recessive nonsyndromic hearing loss DFNB99. Hum Mutat 36, 98-105. 10.1002/humu.22712.
[S227] Liaqat, K., Hussain, S., Bilal, M., Nasir, A., Acharya, A., Ali, R.H., Nawaz, S., Umair, M., Schrauwen, I., Ahmad, W., and Leal, S.M. (2020). Further evidence of involvement of TMEM132E in autosomal recessive nonsyndromic hearing impairment. J Hum Genet 65, 187-192. 10.1038/s10038-019-0691-4.
[S228] Naz, S., Giguere, C.M., Kohrman, D.C., Mitchem, K.L., Riazuddin, S., Morell, R.J., Ramesh, A., Srisailpathy, S., Deshmukh, D., Riazuddin, S., et al. (2002). Mutations in a novel gene, TMIE, are associated with hearing loss linked to the DFNB6 locus. Am J Hum Genet 71, 632-636. 10.1086/342193.

[S229] Zhao, B., Wu, Z., Grillet, N., Yan, L., Xiong, W., Harkins-Perry, S., and Müller, U. (2014). TMIE is an essential component of the mechanotransduction machinery of cochlear hair cells. Neuron 84, 954-967. 10.1016/j.neuron.2014.10.041.
[S230] Scott, H.S., Kudoh, J., Wattenhofer, M., Shibuya, K., Berry, A., Chrast, R., Guipponi, M., Wang, J., Kawasaki, K., Asakawa, S., et al. (2001). Insertion of beta-satellite repeats identifies a transmembrane protease causing both congenital and childhood onset autosomal recessive deafness. Nat Genet 27, 59-63. 10.1038/83768.
[S231] Fasquelle, L., Scott, H.S., Lenoir, M., Wang, J., Rebillard, G., Gaboyard, S., Venteo, S., François, F., Mausset-Bonnefont, A.L., Antonarakis, S.E., et al. (2011). Tmprss3, a transmembrane serine protease deficient in human DFNB8/10 deafness, is critical for cochlear hair cell survival at the onset of hearing. J Biol Chem 286, 17383-17397. 10.1074/jbc.M110.190652.
[S232] Zhao, Y., Zhao, F., Zong, L., Zhang, P., Guan, L., Zhang, J., Wang, D., Wang, J., Chai, W., Lan, L., et al. (2013). Exome sequencing and linkage analysis identified tenascin-C (TNC) as a novel causative gene in nonsyndromic hearing loss. PLoS One 8, e69549. 10.1371/journal.pone.0069549.
[S233] Son, E.J., Wu, L., Yoon, H., Kim, S., Choi, J.Y., and Bok, J. (2012). Developmental gene expression profiling along the tonotopic axis of the mouse cochlea. PLoS One 7, e40735. 10.1371/journal.pone.0040735.
[S234] Rehman, A.U., Morell, R.J., Belyantseva, I.A., Khan, S.Y., Boger, E.T., Shahzad, M., Ahmed, Z.M., Riazuddin, S., Khan, S.N., Riazuddin, S., and Friedman, T.B. (2010). Targeted capture and next-generation sequencing identifies C9orf75, encoding taperin, as the mutated gene in nonsyndromic deafness DFNB79. Am J Hum Genet 86, 378-388. 10.1016/j.ajhg.2010.01.030.
[S235] Li, Y., Pohl, E., Boulouiz, R., Schraders, M., Nürnberg, G., Charif, M., Admiraal, R.J., von Ameln, S., Baessmann, I., Kandil, M., et al. (2010). Mutations in TPRN cause a progressive form of autosomal-recessive nonsyndromic hearing loss. Am J Hum Genet 86, 479-484. 10.1016/j.ajhg.2010.02.003.
[S236] Riazuddin, S., Khan, S.N., Ahmed, Z.M., Ghosh, M., Caution, K., Nazli, S., Kabra, M., Zafar, A.U., Chen, K., Naz, S., et al. (2006). Mutations in TRIOBP, which encodes a putative cytoskeletal-organizing protein, are associated with nonsyndromic recessive deafness. Am J Hum Genet 78, 137-143. 10.1086/499164.
[S237] Babahosseini, H., Belyantseva, I.A., Yousaf, R., Tona, R., Hadi, S., Inagaki, S., Wilson, E., Kitajiri, S.I., Frolenkov, G.I., Friedman, T.B., and Cartagena-Rivera, A.X. (2022). Unbalanced bidirectional radial stiffness gradients within the organ of Corti promoted by TRIOBP. Proc Natl Acad Sci U S A 119, e2115190119. 10.1073/pnas.2115190119.
[S238] Shahin, H., Walsh, T., Sobe, T., Abu Sa'ed, J., Abu Rayan, A., Lynch, E.D., Lee, M.K., Avraham, K.B., King, M.C., and Kanaan, M. (2006). Mutations in a novel isoform of TRIOBP that encodes a filamentous-actin binding protein are responsible for DFNB28 recessive nonsyndromic hearing loss. Am J Hum Genet 78, 144-152. 10.1086/499495.
[S239] Xia, W., Hu, J., Ma, J., Huang, J., Wang, X., Jiang, N., Zhang, J., Ma, Z., and Ma, D. (2019). Novel TRRAP mutation causes autosomal dominant non-syndromic hearing loss. Clin Genet 96, 300-308. 10.1111/cge.13590.
[S240] Delmaghani, S., Aghaie, A., Michalski, N., Bonnet, C., Weil, D., and Petit, C. (2012). Defect in the gene encoding the EAR/EPTP domain-containing protein TSPEAR causes DFNB98 profound deafness. Hum Mol Genet 21, 3835-3844. 10.1093/hmg/dds212.
[S241] Morino, H., Pierce, S.B., Matsuda, Y., Walsh, T., Ohsawa, R., Newby, M., Hiraki-Kamon, K., Kuramochi, M., Lee, M.K., Klevit, R.E., et al. (2014). Mutations in Twinkle primase-helicase cause Perrault syndrome with neurologic features. Neurology 83, 2054-2061. 10.1212/wnl.0000000000001036.
[S242] Verpy, E., Leibovici, M., Zwaenepoel, I., Liu, X.Z., Gal, A., Salem, N., Mansour, A., Blanchard, S., Kobayashi, I., Keats, B.J., et al. (2000). A defect in harmonin, a PDZ domain-containing protein expressed in the inner ear sensory hair cells, underlies Usher syndrome type 1C. Nat Genet 26, 51-55. 10.1038/79171.
[S243] Ahmed, Z.M., Smith, T.N., Riazuddin, S., Makishima, T., Ghosh, M., Bokhari, S., Menon, P.S., Deshmukh, D., Griffith, A.J., Riazuddin, S., et al. (2002). Nonsyndromic recessive deafness DFNB18 and Usher syndrome type IC are allelic mutations of USHIC. Hum Genet 110, 527-531. 10.1007/s00439-002-0732-4.
[S244] Weil, D., El-Amraoui, A., Masmoudi, S., Mustapha, M., Kikkawa, Y., Lainé, S., Delmaghani, S., Adato, A., Nadifi, S., Zina, Z.B., et al. (2003). Usher syndrome type I G (USH1G) is caused by mutations in the gene encoding SANS, a protein that associates with the USH1C protein, harmonin. Hum Mol Genet 12, 463-471. 10.1093/hmg/ddg051.
[S245] Eudy, J.D., Weston, M.D., Yao, S., Hoover, D.M., Rehm, H.L., Ma-Edmonds, M., Yan, D., Ahmad, I., Cheng, J.J., Ayuso, C., et al. (1998). Mutation of a gene encoding a protein with extracellular matrix motifs in Usher syndrome type IIa. Science 280, 1753-1757. 10.1126/science.280.5370.1753.
[S246] Buniello, A., Ingham, N.J., Lewis, M.A., Huma, A.C., Martinez-Vega, R., Varela-Nieto, I., Vizcay-Barrena, G., Fleck, R.A., Houston, O., Bardhan, T., et al. (2016). Wbp2 is required for normal glutamatergic synapses in the cochlea and is crucial for hearing. EMBO Mol Med 8, 191-207. 10.15252/emmm.201505523.
[S247] Bespalova, I.N., Van Camp, G., Bom, S.J., Brown, D.J., Cryns, K., DeWan, A.T., Erson, A.E., Flothmann, K., Kunst, H.P., Kurnool, P., et al. (2001). Mutations in the Wolfram syndrome 1 gene (WFS1) are a common cause of low frequency sensorineural hearing loss. Hum Mol Genet 10, 2501-2508. 10.1093/hmg/10.22.2501.
[S248] Young, T.L., Ives, E., Lynch, E., Person, R., Snook, S., MacLaren, L., Cater, T., Griffin, A., Fernandez, B., Lee, M.K., and King, M.C. (2001). Non-syndromic progressive hearing loss DFNA38 is caused by heterozygous missense mutation in the Wolfram syndrome gene WFS1. Hum Mol Genet 10, 2509-2514. 10.1093/hmg/10.22.2509.
[S249] Cryns, K., Thys, S., Van Laer, L., Oka, Y., Pfister, M., Van Nassauw, L., Smith, R.J., Timmermans, J.P., and Van Camp, G. (2003). The WFS1 gene, responsible for low frequency sensorineural hearing loss and Wolfram syndrome, is expressed in a variety of inner ear cells. Histochem Cell Biol 119, 247-256. 10.1007/s00418-003-0495-6.
[S250] Bramhall, N.F., Kallman, J.C., Verrall, A.M., and Street, V.A. (2008). A novel WFS1 mutation in a family with dominant low frequency sensorineural hearing loss with normal VEMP and EcochG findings. BMC Med Genet 9, 48. 10.1186/1471-2350-9-48.

[S251] Mburu, P., Mustapha, M., Varela, A., Weil, D., El-Amraoui, A., Holme, R.H., Rump, A., Hardisty, R.E., Blanchard, S., Coimbra, R.S., et al. (2003). Defects in whirlin, a PDZ domain molecule involved in stereocilia elongation, cause deafness in the whirler mouse and families with DFNB31. Nat Genet 34, 421-428. 10.1038/ng1208.
[S252] Ebermann, I., Scholl, H.P., Charbel Issa, P., Becirovic, E., Lamprecht, J., Jurklies, B., Millán, J.M., Aller, E., Mitter, D., and Bolz, H. (2007). A novel gene for Usher syndrome type 2: mutations in the long isoform of whirlin are associated with retinitis pigmentosa and sensorineural hearing loss. Hum Genet 121, 203-211. 10.1007/s00439-006-0304-0.
[S253] Mathur, P.D., and Yang, J. (2019). Usher syndrome and non-syndromic deafness: Functions of different whirlin isoforms in the cochlea, vestibular organs, and retina. Hear Res 375, 14-24. 10.1016/j.heares.2019.02.007.
[S254] Stemerdink, M., García-Bohórquez, B., Schellens, R., Garcia-Garcia, G., Van Wijk, E., and Millan, J.M. (2022). Genetics, pathogenesis and therapeutic developments for Usher syndrome type 2. Hum Genet 141, 737-758. 10.1007/s00439-021-02324-w.
